# Synthesis, Characterization, and Evaluation of the Cobalt–Carbon Bond Strength of (PNNP)Co^III^-R Complexes

**DOI:** 10.1021/acs.organomet.6c00008

**Published:** 2026-04-02

**Authors:** Justin D. Miller, Mitchell M. Walsh, Curtis E. Moore, Christine M. Thomas

**Affiliations:** Department of Chemistry and Biochemistry, 2647 The Ohio State University, Columbus, Ohio 43210, United States

## Abstract

Square pyramidal cobalt­(III)-alkyl complexes ((PNCH_2_CH_2_NP)­Co^III^-R (**1-R**) and (PNCHCHNP)­Co^III^-R (**2-R**), R = CH_3_, ^*n*^Bu, or Bn) were synthesized via oxidative addition of alkyl halides to [(PNCH_2_CH_2_NP)­Co^I^]­[Na­(THF)_n_] (**1-Na**) or [(PNCHCHNP)­Co^I^]­[Na­(THF)_n_] (**2-Na**). The redox-active ligand of (PNCHCHNP)­Co^II^ (**2**) provides access to the one-electron oxidized species [(PNCHCHNP)­Co­(THF)]­[PF_6_] (**2-PF**_**6**_), allowing synthesis of **2-R** compounds through alkylation of **2-PF**_**6**_ with carbanions. Radical trapping experiments with TEMPO and analysis of the thermal decomposition products suggest cobalt–carbon bond homolysis as the primary decomposition pathway for these molecules, although the homolysis products are dependent on the equatorial ligand framework and the identity of the alkyl substituent. The stability of the cobalt–carbon bond was evaluated through kinetic and computational methods. We suggest a more distorted equatorial ligand destabilizes the cobalt–carbon bond, while the electron-rich phosphine substituents stabilize the cobalt–carbon bond compared to previously reported cobalamin model compounds. Catalytic investigations into radical Heck-type cross-coupling found (PNCH_2_CH_2_NP)­Co^II^ (**1**) to be a more active catalyst than **2**, although unwanted side products resulted in reduced yields for the desired cross-coupled product.

## Graphical abstract

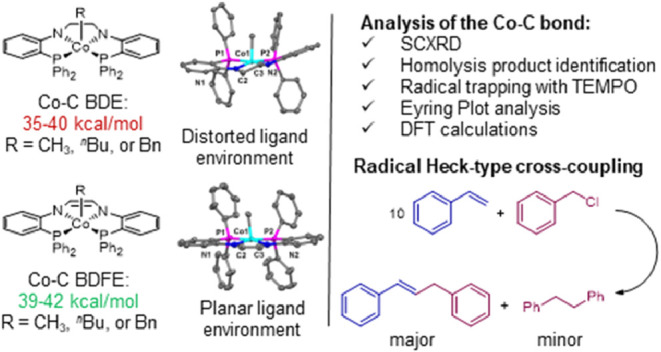

## Introduction

The discovery and structural determination of the cobalamin enzymes has inspired the synthesis of numerous cobalt­(III)-alkyl complexes. These cobalt­(III)-alkyl complexes are generally five- or six-coordinate and often ligated by a planar tetradentate ligand that occupies the equatorial coordination sites of the cobalt center. Common equatorial ligand scaffolds include porphyrin,^1–4^ phthalocyanine,^5,6^ cobaloxime,^7–10^ Costa,^11,12^ TIM,^13–16^ salen,^17^ salophen,^18,19^ tropocoronand,^20^ and derivatives thereof (Figure 1). A shared reactivity pattern of these cobalt­(III)-alkyl complexes is the facile homolysis of the cobalt–carbon bond, which is often reversible, giving rise to the persistent radical effect (PRE).^21,22^ Cobalt-mediated radical polymerization takes advantage of the PRE by using a cobalt catalyst as a reversible radical trap to control the concentration of radicals in solution, and by extension, the properties of the synthesized polymer.^23–26^ The facile homolysis of the cobalt–carbon bond in cobalt­(III)-alkyl complexes makes them attractive catalysts for radical-type catalysis including cyclization,^27^ cross-coupling,^27–30^ hydrofunctionalization,^27^ perfluoroalkylation,^31^ dehydrohalogenation,^32^ and isomerization.^27^

**Figure 1:**
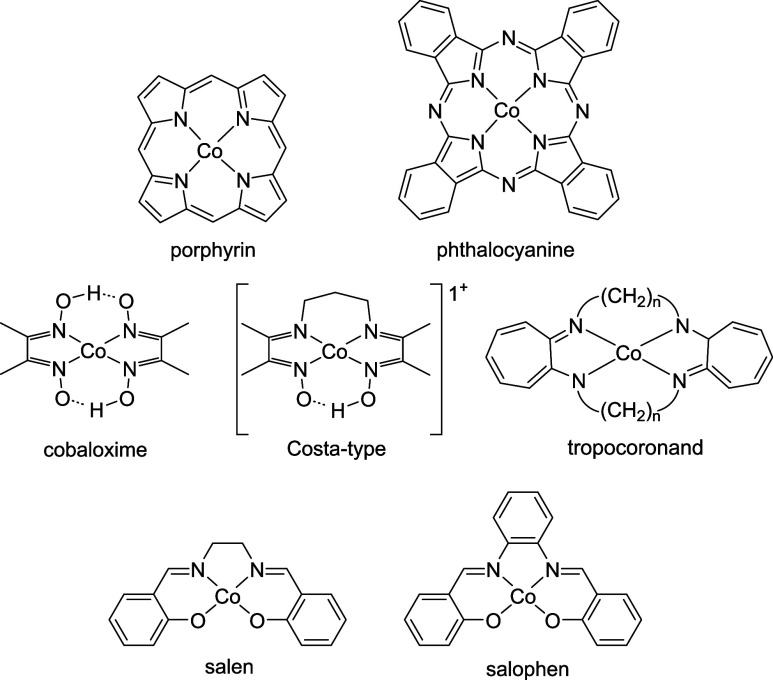
Common ligand scaffolds for cobalamin model compounds.

Whether used as a catalyst or a biomimetic analogue of Vitamin B_12_, the factors influencing the strength of the cobalt–carbon bond in cobalt­(III)-alkyl complexes are of interest. Substantial research has investigated the effects of the axially coordinated ligands on the cobalt–carbon bond strength and have concluded that increasing steric bulk of the alkyl substituent or a coordinated axial base significantly weakens the cobalt–carbon bond.^9,33–38^ To a lesser degree, increasing the basicity of the axial base was shown to stabilize the cobalt–carbon bond by favoring the cobalt­(III) oxidation state,^9,34,36^ although cobalt­(III)-alkyl complexes without a coordinated axial base were found to have stronger cobalt–carbon bonds than complexes with an axial base.^3,36^

There is a general consensus regarding the effects of the alkyl substituent and axial base on the cobalt–carbon bond strength, however, the role of the equatorial ligand is more ambiguous. Liu and co-workers computed the cobalt–carbon bond dissociation enthalpies for a variety of square-planar four-coordinate equatorial ligand frameworks and concluded that the cobalt–carbon bond dissociation enthalpy increases as the electronegativity of the equatorial ligand increases.^39^ They attribute this to the *anti*-spin-delocalization effect, in which the more electronegative equatorial ligands create a more polarized cobalt–carbon bond, increasing cobalt–carbon bond stability to homolysis. Alternatively, Galezowski and Sawyer provided experimental evidence suggesting the stability of the cobalt–carbon bond increases as the basicity of the equatorial ligand increases,^5,40^ likely stabilizing the cobalt­(III) oxidation state. Steric influences also impact cobalt–carbon bond strength, specifically the flexibility of the equatorial ligand. Lippard and co-workers investigated cobalt­(III)-methyl complexes in the tropocoronand ligand system where the more flexible CoMe­(TC-4,4) complex underwent cobalt–carbon bond homolysis 13 times faster than the more rigid CoMe­(TC-3,3) analogue.^20^ Similarly, Halpern and co-workers showed that the cobalt–carbon bond dissociation energy in the more rigid porphyrin ligand framework was insensitive to the bulkiness of an axially coordinated phosphine, while the cobalt–carbon bond dissociation energy in the more flexible dimethylglyoxime analogues decreased as the steric bulk of the phosphine increased.^33^

Herein, we report a new set of square pyramidal cobalt­(III)-alkyl compounds with phosphine ligands incorporated into the equatorial framework. We compare the cobalt–carbon bond strength of six cobalt­(III)-alkyl complexes, three of which are derived from (PNCH_2_CH_2_NP)­Co^II^ (**1**), which has a redox-inert ligand with a saturated diamide backbone, and three are derived from (PNCHCHNP)­Co^II^ (**2**), featuring a more rigid unsaturated ene-diamide ligand backbone. Ultimately, we find that the more flexible ligand backbone renders alkyl compounds derived from **1** to be more prone to cobalt–carbon bond homolysis and this enhanced reactivity is showcased through a catalytic radical Heck-type cross-coupling reaction.

## Results

### Synthesis and Characterization

The previously reported reduction of **1** and **2** using KC_8_ required 18-crown-6 for purification via recrystallization.^41^ An alternate route of reduction without 18-crown-6 was pursued to generate a cobalt­(I) starting material to facilitate purification of subsequent oxidative addition products. Reduction of **1** proceeded through addition of a THF solution of **1** to 1.5 equiv of 0.5% Na/Hg amalgam, resulting in an immediate color change from brown to emerald green and affording [(PNCH_2_CH_2_NP)­Co^I^]­[Na­(THF)*_n_*] (**1-Na**, *n* = 1–3) in 91.4% yield (Scheme 1). Similarly, a THF solution of **2** was added to 1.5 equiv of 0.5% Na/Hg amalgam, producing the one-electron reduced product [(PNCHCHNP)­Co^I^]­[Na­(THF)*_n_*] (**2-Na**, *n* = 0–3) as a brown solid in 89.7% yield (Scheme 1). A single resonance is observed in the ^31^P­{^1^H} NMR spectra of **1-Na** (59.30 ppm) and **2-Na** (60.32 ppm), consistent with the previously reported potassium salts (Figures S3 and S6).^41^ Single crystals suitable for X-ray diffraction were obtained for **1-Na** and **2-Na** to confirm the connectivity of the proposed structures (Figure 2). In both cases, the THF-bound Na^+^ counterion loosely associates with one of the two amido nitrogen atoms of the ligand.

**Figure 2:**
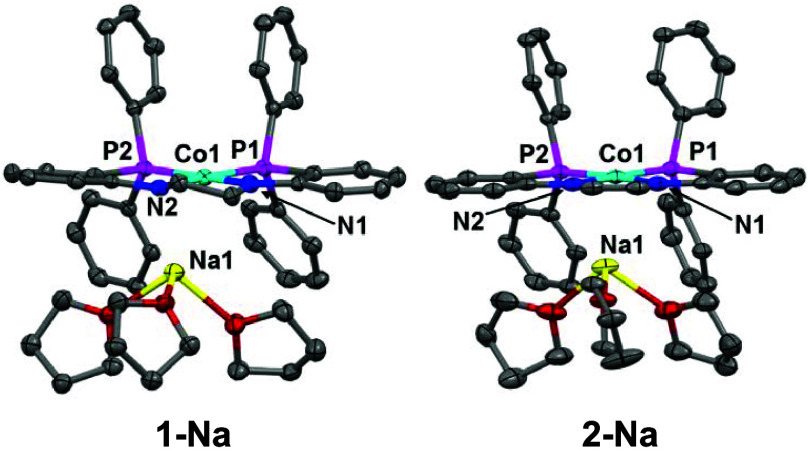
Displacement ellipsoid (50%) representations of **1-Na** (left) and **2-Na** (right). All H atoms and solvate molecules were omitted for clarity. Disorder was observed on some of the THF molecules bound to sodium, only one representation is shown here for clarity.

**Scheme 1:**
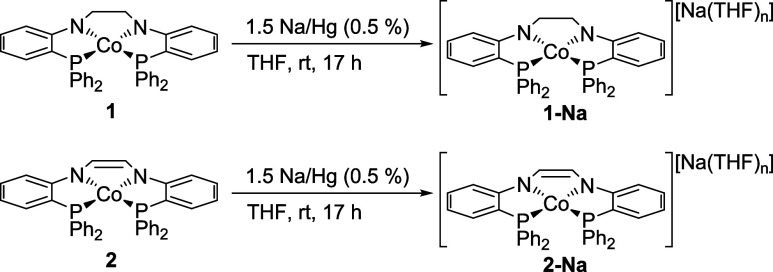
Synthesis of **1-Na** and **2-Na**

Addition of CH_3_I, ^*n*^BuBr, or BnBr to a THF solution of **1-Na**, afforded the corresponding cobalt­(III)-alkyl complexes **1-CH**_**3**_, **1-Bu**, and **1-Bn**, respectively (Scheme 2a). In each reaction, a color change from emerald-green to orange (**1-CH**_**3**_), brown (**1-Bu**), or purple (**1-Bn**) was observed. Similarly, CH_3_I, ^*n*^BuBr, or BnBr were added to a THF solution of **2-Na**, producing cobalt­(III)-alkyl complexes **2-CH**_**3**_, **2-Bu**, or **2-Bn**, respectively (Scheme 2a). Alternatively, addition of CH_3_Li, ^*n*^BuLi, or BnMgCl to the one-electron oxidation product of **2**, **2-PF**_**6**_,^41^ provides the alkylation products **2-CH**_**3**_, **2-Bu**, or **2-Bn** (Scheme 2b). In all cases, a color change from brown-orange to green was observed for the oxidative addition of alkyl halides to **2-Na**, while a color change from purple to green was observed for the alkylation reactions starting with **2-PF**_**6**_.^41^

**Scheme 2:**
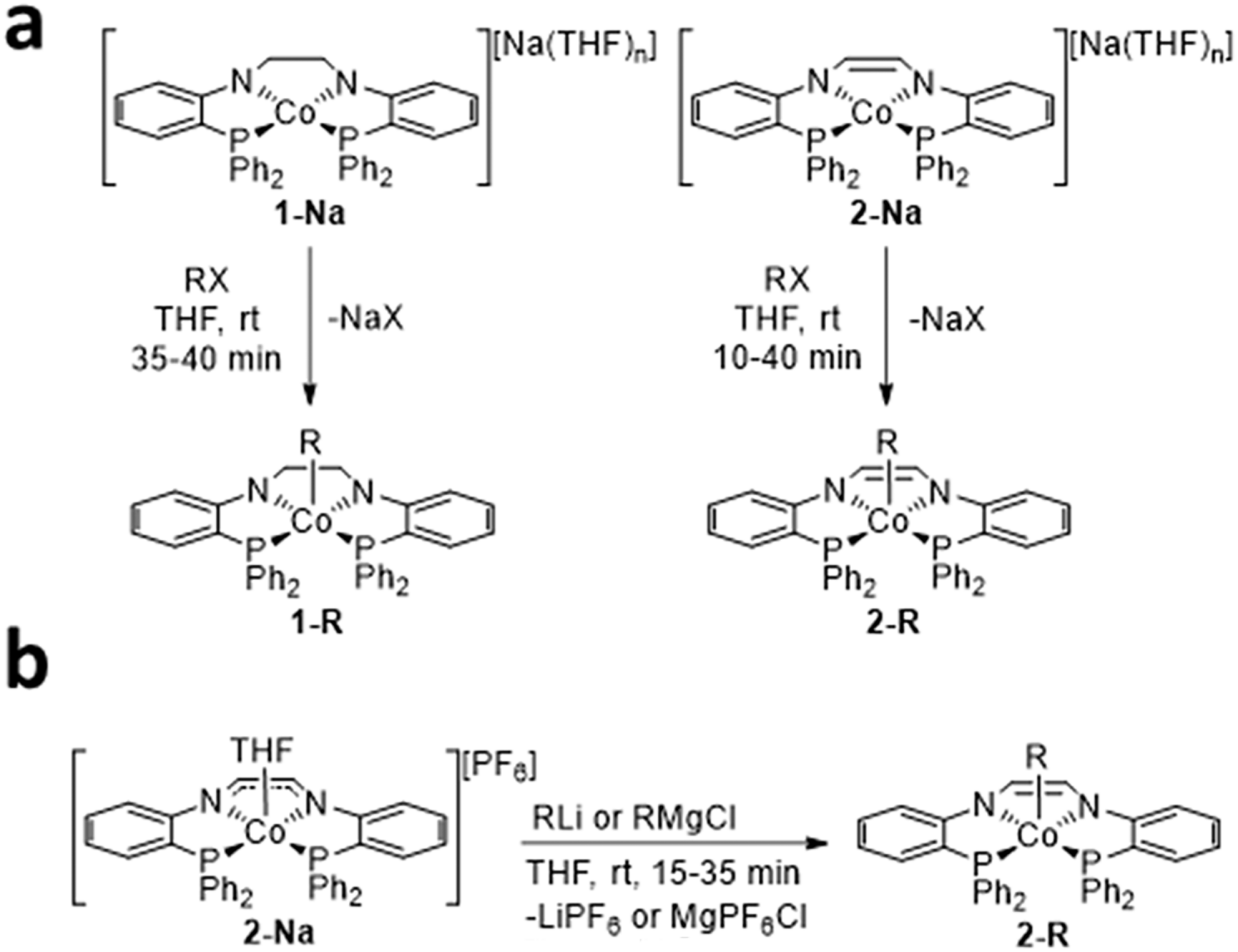
(a) Synthesis of **1-R** and **2-R** (R = CH_3_, ^*n*^Bu, Bn) via Oxidative Addition of RX (RX = CH_3_I, ^*n*^BuBr, or BnBr) to **1-Na** and **2-Na** (b) Synthesis of 2-R (R = CH_3_, ^*n*^Bu, Bn) via Alkylation of 2-PF_6_

The ^1^H and ^31^P­{^1^H} NMR spectra of **1-CH**_**3**_, **1-Bu** and **1-Bn** are consistent with low-spin diamagnetic cobalt­(III) complexes (see Table 1 for a list of relevant resonances). A triplet at 0.46 ppm integrating to three protons is observed in the ^1^H NMR spectrum of **1-CH**_**3**_, corresponding to the CH_3_ group bound to the cobalt­(III) center (Figure S8). Two multiplets integrating to two protons each were detected at 3.55 and 3.83 ppm, corresponding to the backbone protons of **1-CH**_**3**_, indicating a loss of *C*_2_ symmetry, while a single peak in the ^31^P­{^1^H} NMR spectrum at 51.15 ppm signifies retention of the vertical mirror plane (Figures S8 and S10).

**Table 1:** ^1^H and ^31^P­{^1^H} NMR Spectroscopy Data for Derivatives **1-R** and **2-R** ^a^

Cmpd	^31^P{^1^H} (ppm)	^1^H, alkyl group (ppm)	^1^H, backbone (ppm)
**1-CH**_**3**_	51.15	0.46	3.55, 3.83
**2-CH**_**3**_	55.07	–0.05	7.50
**1-Bu**	52.33	0.42, 0.65, 1.15, 1.24	3.65, 3.88
**2-Bu**	55.53	0.46, 0.65, 0.84	7.46
**1-Bn**	50.26	2.28	3.02, 3.15
**2-Bn**	54.14	1.97	7.14

a Chemical shifts reported in C_6_D_6_.

The ^1^H NMR spectrum of **1-Bu** displays four resonances (0.42, 0.65, 1.15, and 1.24 ppm) integrating 2:2:2:3, consistent with an *n*-butyl group bound to the cobalt­(III) center (Figure S11). Similar to **1-CH**_**3**_, the protons corresponding to the backbone of **1-Bu** were identified as multiplets at 3.65 and 3.88 ppm, with each resonance integrating to two protons (Figure S11). A single resonance is observed in the ^31^P­{^1^H} NMR spectrum of **1-Bu** at 52.33 ppm (Figure S12).

A triplet was detected in the ^1^H NMR spectrum of **1-Bn** at 2.28 ppm integrating to two protons, representing the benzylic protons of the benzyl group bound to the cobalt­(III) center (Figure S15). The resonances for the backbone protons of **1-Bn** were identified as multiplets at 3.02 and 3.15 ppm, integrating to two protons each (Figure S15). A single resonance in the ^31^P­{^1^H} NMR spectrum of **1-Bn** was observed at 50.26 ppm (Figure S16).

Similar to the **1-R** analogues, the ^1^H and ^31^P­{^1^H} NMR spectra of **2-CH**_**3**_, **2-Bu** and **2-Bn** indicate low-spin diamagnetic cobalt­(III) complexes (see Table 1 for a list of relevant resonances). The ^1^H NMR spectrum of **2-CH**_**3**_ displays a triplet at −0.05 ppm integrating to three protons, identified as the cobalt-bound CH_3_ group (Figure S19) that is shifted upfield compared to **1-CH**_**3**_ (0.46 ppm). A singlet at 7.50 ppm corresponding to two protons was also observed and assigned as the two backbone protons of **2-CH**_**3**_. The downfield shift, change in splitting pattern, and reduced number of backbone protons when compared to **1-CH**_**3**_ are consistent with maintenance of the unsaturated ligand backbone in **2-CH**_**3**_. The single resonance in the ^31^P­{^1^H} NMR spectrum of **2-CH**_**3**_ (55.07 ppm, Figure S20) is shifted downfield compared to **1-CH**_**3**_ (51.15 ppm).

The resonances corresponding to the butyl group bound to cobalt in **2-Bu** are observed in the ^1^H NMR spectrum at 0.84, 0.65, and 0.46 ppm integrating 4:2:3, respectively, while a single resonance was detected in the ^31^P­{^1^H} NMR spectrum at 55.53 ppm (Figures S22 and 23). The two protons for the backbone of **2-Bu** were identified as a singlet at 7.46 ppm in the ^1^H NMR spectrum (Figure S22). As described for the methyl derivatives, an upfield shift is observed in the ^1^H NMR spectrum for the resonances corresponding to the protons on the cobalt-bound alkyl when comparing **2-Bu** and **1-Bu**, while the ^31^P­{^1^H} resonance of **2-Bu** (55.53 ppm) is shifted downfield compared to **1-Bu** (52.33 ppm).

The trends in the ^1^H and ^31^P­{^1^H} NMR spectra are preserved when comparing **2-Bn** and **1-Bn**. The ^1^H NMR spectrum of **2-Bn** features a triplet integrating to two protons at 1.97 ppm (2.28 ppm for **1-Bn**) assigned to the benzylic protons of the cobalt-bound benzyl group (Figure S26). A resonance for the backbone protons of **2-Bn** was assigned to a singlet at 7.14 ppm (Figure S26), similar to the **2-CH**_**3**_ and **2-Bu** derivatives (*vide supra*). The ^31^P­{^1^H} NMR spectrum of **2-Bn** (Figure S27) exhibits a single resonance at 54.14 ppm, shifted almost 4 ppm downfield compared to **1-Bn** (50.26 ppm).

Single crystals suitable for X-ray diffraction were obtained for **1-CH**_**3**_, **1-Bn**, **2-CH**_**3**_, **2-Bu**, and **2-Bn** (Figure 3). All five cobalt­(III)-alkyl complexes crystallized in the expected square pyramidal geometry (τ_5_ ^42^ = 0.10, 0.04, 0.01, 0.02, and 0.0, respectively) for five-coordinate cobalt­(III)-alkyl complexes coordinated to a planar, κ^4^ equatorial ligand. The equatorial ligand also influences the cobalt–carbon bond distances, with the **1-R** molecules displaying slightly shorter cobalt–carbon bonds when compared to the unsaturated **2-R** derivatives (Table 2).

**Figure 3:**
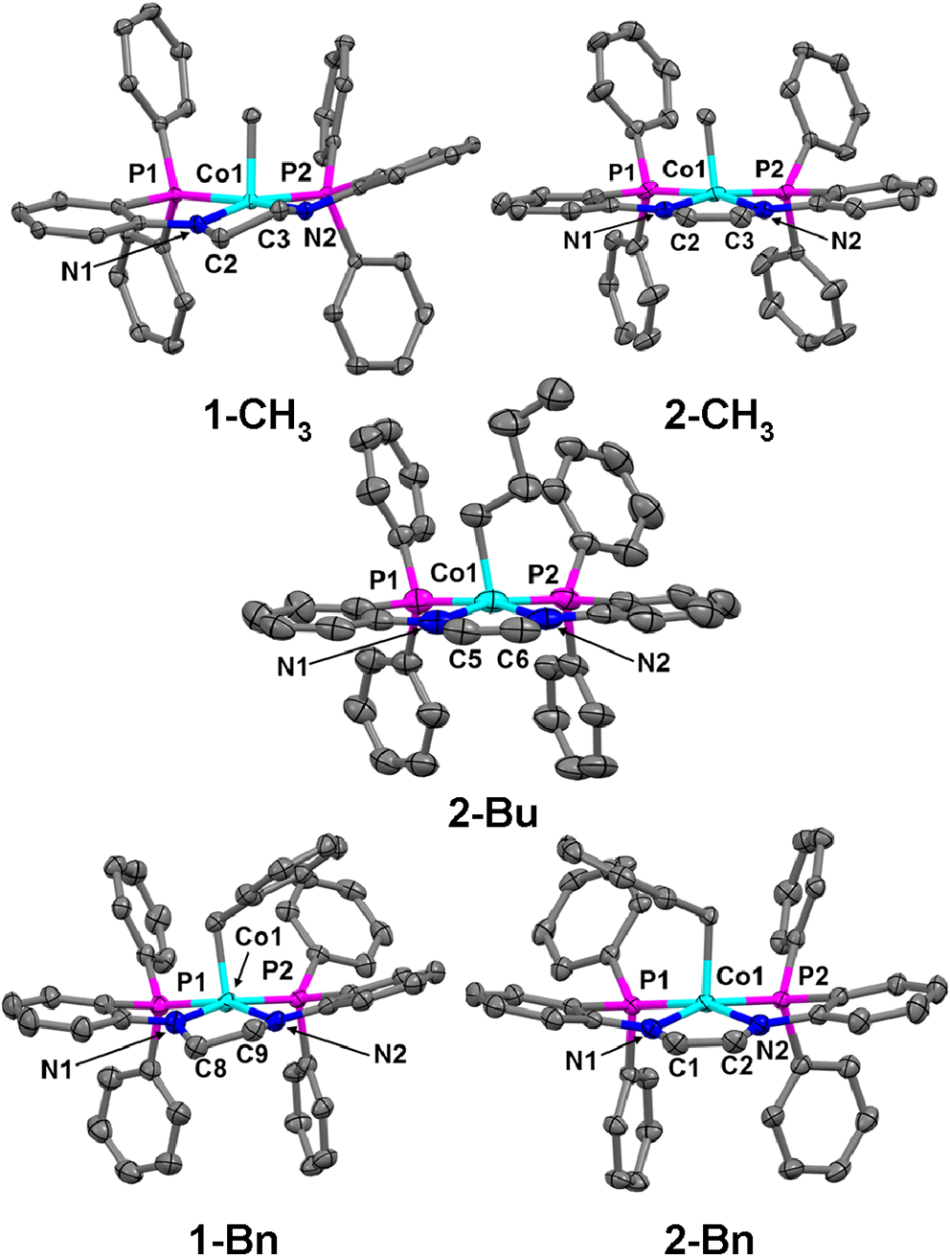
Displacement ellipsoid (50%) representations of **1-CH**_**3**_, **2-CH**_**3**_, **2-Bu**, **1-Bn**, and **2-Bn**. All H atoms and solvate molecules were removed for clarity. Disorder was observed on the methyl group and one of the aromatic rings bound to a phosphine ligand in the structure of **2-CH**_**3**_. Disorder was also observed on the butyl group of **2-Bu**. Only one representation of **2-CH**_**3**_ and **2-Bu** are shown for clarity.

**Table 2:** Selected Geometric Parameters for Compounds **1-R** and **2-R**

Cmpd	Co–C (Å)	Co–N_avg_ (Å)	Co–P_avg_ (Å)	NCCN (°)
**1-CH**_**3**_	1.9897(15)	1.8843(18)	2.1954(6)	–32.9(2)
**1-Bn**	2.0245(19)	1.8860(23)	2.1856(8)	30.6(2)
**2-CH**_**3**_	2.0005(16)^a^	1.8797(23)	2.1844(8)	–0.1(3)
**2-Bu**	2.067(7)^b^	1.872(4)	2.1715(14)	0.3(5)
**2-Bn**	2.044(2)	1.8836(19)	2.1731(6)	0.9(2)

a Average value of two distances due to disorder.

b Distance reported is from the representation with the highest occupancy.

The unsaturated [PNCHCHNP]^2–^ ligand was determined to be redox-active in our previous report.^41^ It is, therefore, conceivable that **2-CH**_**3**_, **2-Bu**, and **2-Bn** could be described as cobalt­(II) complexes with a one-electron oxidized ligand, instead of cobalt­(III) complexes. However, there is little discrepancy in the crystallographically determined Co–N bond distances when comparing the **1-R** and **2-R** derivatives (Table 2), suggesting that the saturated and unsaturated ligands are in the same redox state. Additionally, the C–N_avg_ bond distances in **2-R** are more similar to the distances reported for **2** than the one-electron oxidized complex **2-PF**_**6**_ (Table S5).^41^ The C–C backbone bond distances in the **2-R** derivatives indicate a C–C double bond, consistent with retention of the ene-diamide ligand state following alkylation and assignment of **2-R** as bona fide cobalt­(III) alkyls.

### Electrochemical Analysis of **1-R** and **2-R**

Cyclic voltammograms (CVs) were collected for the **1-R** and **2-R** derivatives (Figure 4). In all cases a reduction at the cobalt­(III) center was observed, with the reversibility and onset of the reduction dependent on the identity of the alkyl substituent. For molecules with the same equatorial ligand, decreasing the electron donating properties of the alkyl (^*n*^Bu > CH_3_ > Bn) shifts the reduction potential anodically as reduction of the cobalt­(III) center becomes more facile with less electron-rich ligands. The identity of the alkyl substituent also impacts the reversibility of the reduction: The methyl compounds **1-CH**_**3**_ and **2-CH**_**3**_ display reversible redox events, while the butyl and benzyl derivatives display irreversible reductions. Shifts in the potentials of the Co^III/II^ reduction are also observed when comparing the two equatorial ligand frameworks. The unsaturated **2-R** derivatives were found to have cathodically shifted reductions compared to the **1-R** derivatives with the same alkyl group. This indicates that the cobalt­(III) center in the **2-R** derivatives is more electron rich than in their **1-R** counterparts.

**Figure 4:**
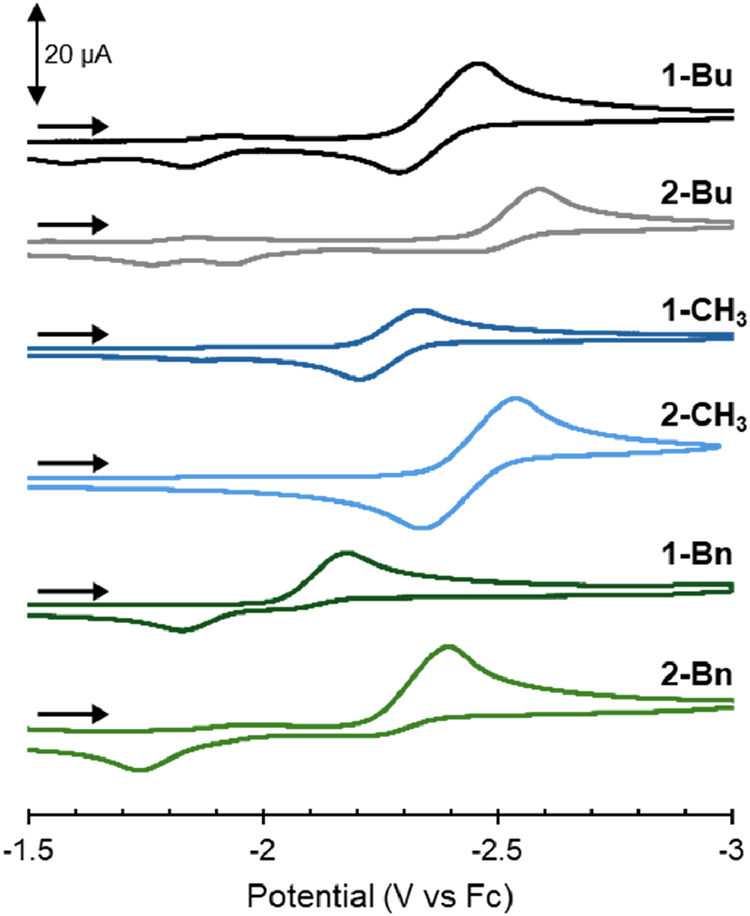
Cyclic voltammograms of the **1-R** and **2-R** derivatives in a 0.1 M [^*n*^Bu_4_N]­[PF_6_] THF solution (scan rate = 100 mV s^–1^). All potentials are referenced to Fc/Fc^+^.

### Thermolysis Products and Reactivity with TEMPO

We hypothesized that the primary decomposition pathway of **1-R** and **2-R** analogues would proceed through cobalt–carbon bond homolysis, similar to previously reported cobalt­(III)-alkyl complexes.^43^ Derivatives of **1-R** and **2-R** were dissolved in C_6_D_6_, sealed in an NMR tube, and the resulting decomposition products monitored by ^1^H NMR spectroscopy over time, applying heat if degradation was not observed at room temperature (Figures S29–39). In all cases, homolysis of the cobalt–carbon bond was the primary decomposition pathway, although the observed decomposition products and cobalt–carbon bond stability varied depending on the alkyl substituent and ligand framework (Scheme 3).

**Scheme 3:**
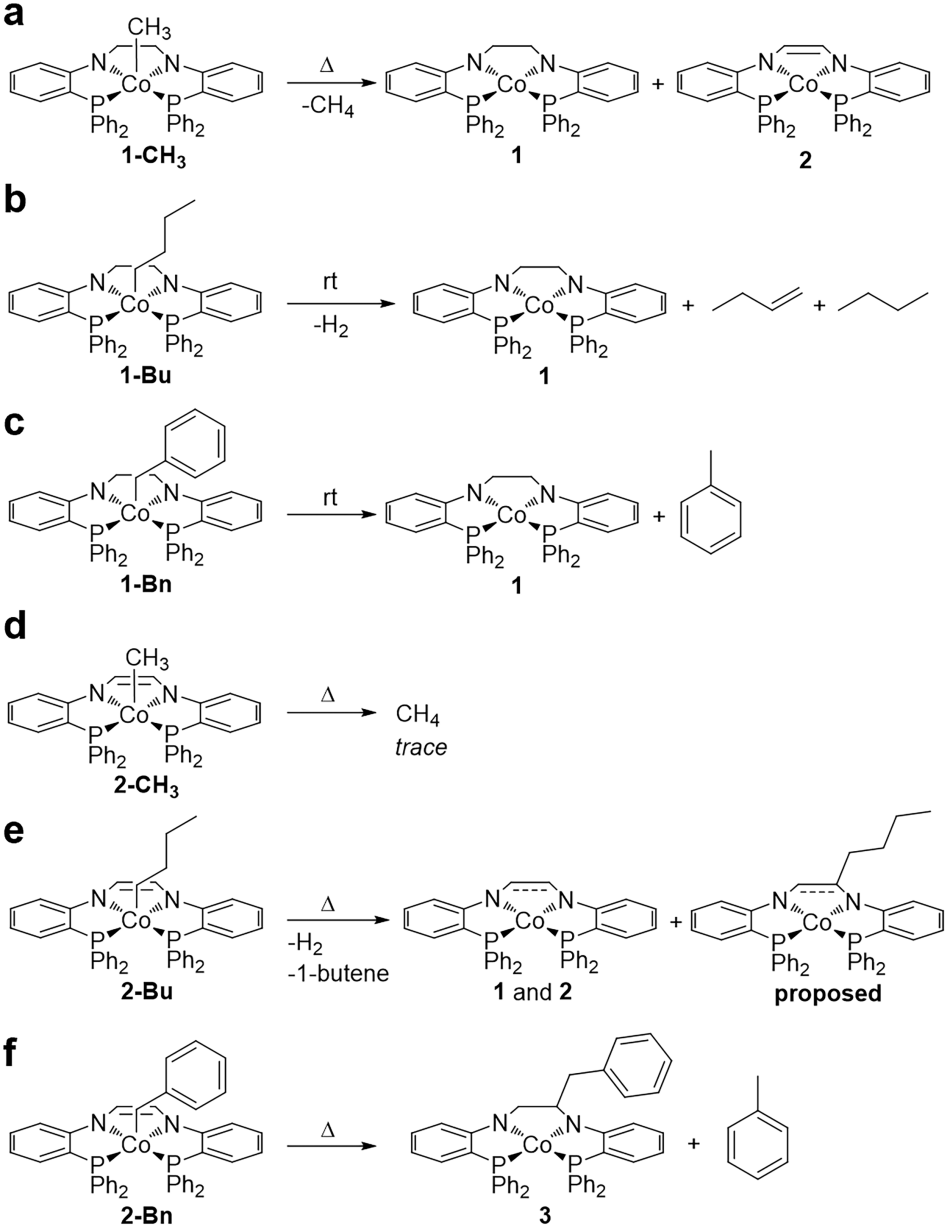
Thermolysis Products of Cobalt­(III)-Alkyl Complexes

Thermal decomposition of **1-CH**_**3**_ was observed at temperatures ranging from 40–60 °C, with **1**, CH_4_, and **2** as the primary products (Scheme 3a and Figure S29). Formation of **2** and CH_4_ suggests the methyl radicals formed during homolysis abstracted hydrogen atoms from the backbone of **1** to produce **2**, a process similar to our previously reported reaction of **1** with TEMPO.^41^ The homocoupling product of methyl radicals (ethane) was not observed in the ^1^H NMR spectrum, further supporting abstraction of hydrogen atoms from the backbone of **1-CH**_**3**_ following cobalt-carbon homolysis. Since two methyl radicals are required for backbone dehydrogenation, full conversion of **1** to **2** was not observed and the products were instead formed in a roughly 2:0.8 ratio. Although carbon-centered radicals produced through cobalt–carbon bond homolysis have been shown to abstract hydrogen atoms,^7,44^ they have not been observed to abstract hydrogen atoms from the metal complex itself.

While following the decomposition of **1-Bu** at room temperature, butene, butane, H_2_, and **1** were detected (Scheme 3b and Figure S30). We posit these products were formed through homolysis of the cobalt–carbon bond, followed by abstraction of the β-hydrogen atom from the butyl radical by **1**, forming butene and a transient cobalt­(III)-hydride species that readily releases 1/2 equiv H_2_, thereby reforming **1**.^9,34,35,45^ Although β-hydride elimination is a possible mechanism to produce the observed products, the rigidity of the (PNNP) ligand framework makes this pathway unlikely. Free butyl radicals are known to react to form butane and butene; during homolysis of the cobalt–carbon bond some of the butyl radicals likely escaped the radical cage resulting in the formation of butane during this reaction. Additionally, radical trapping experiments with TEMPO produced the expected TEMPO-R product (*vide infra*), supporting a radical mechanism invoking cobalt–carbon bond homolysis.

The primary decomposition products of **1-Bn** at room temperature were toluene and **1** (Scheme 3c and Figure S31). Hydrogen atom abstraction from the backbone was not observed in the case of **1-Bn** and the source of hydrogen atoms is currently unknown but may be attributed to adventitious water. Additionally, the homocoupling product, bibenzyl, was not observed by ^1^H NMR spectroscopy.

Thermal treatment of **2-R** derivatives suggests a stronger cobalt–carbon bond in these molecules compared to their **1-R** analogues. When heated to 84 °C, **2-CH**_**3**_ showed minimal decomposition; trace CH_4_ was detected after 14 days, with **2-CH**_**3**_ remaining as the primary metal complex in solution (Scheme 3d and Figure S32).

Decomposition of **2-Bu** was observed at temperatures ranging from 45–80 °C (Figure S33). **2-Bu** did not show signs of decomposition at room temperature. The primary decomposition products were butene, **1** and **2** (in a roughly 1:1 ratio), and an unidentified paramagnetic product (Scheme 3e). It is likely that butene was formed through a similar radical mechanism as described for **1-Bu**. The formation of **1** is observed due to hydrogenation of the backbone of **2** by the H_2_ formed during the decomposition of **2-Bu**, a process observed in our previous work.^41^ The asymmetry and paramagnetism of the unidentified product leads us to propose a complex in which the butyl radical has attacked the unsaturated ligand backbone, similar to the product observed in the thermolysis of **2-Bn** (*vide infra*).

Unlike the previously discussed analogues, **2-Bn** displayed unique products of thermal decomposition. After heating at 50 °C for 2 days, a small amount of toluene and a paramagnetic product were observed as the major species in solution. The peak pattern for the paramagnetic product was inconsistent with the formation of **1** or **2** (Scheme 3f and Figures S38 and 39). Single crystals grown from the isolated crude solid from the reaction produced dark brown blocks suitable for single crystal X-ray diffraction. The refined structure revealed a cobalt­(II) complex with a benzyl group bound to the now-saturated backbone, **3** (Figure 5).

**Figure 5:**
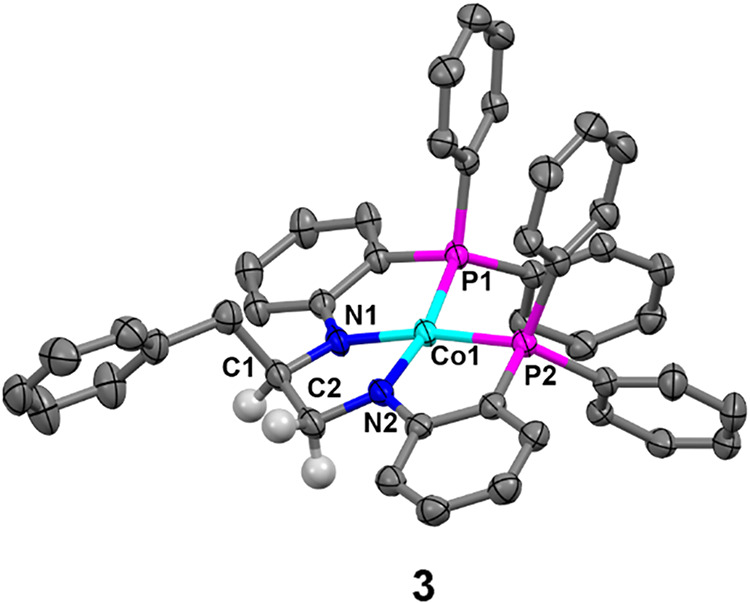
Displacement ellipsoid (50%) representation of **3**. All solvate molecules and H atoms, except those on the ligand backbone, were removed for clarity.

Based on the products observed in the thermolysis experiments discussed previously, we sought to trap the alkyl radicals formed during cobalt–carbon bond homolysis with TEMPO. The radical trapping experiments proceeded through addition of ∼2 equiv TEMPO at room temperature to each cobalt­(III)-alkyl complex (Scheme 4). The reaction between **1-CH**_**3**_ and TEMPO resulted in **2-CH**_**3**_, TEMPO–CH_3_, TEMPO-H, and **1** as the major products (Scheme 4a and Figure S40). We suggest TEMPO is abstracting hydrogen atoms from the backbone of **1-CH**_**3**_ to produce **2-CH**_**3**_, which did not show reactivity with TEMPO at room temperature (*vide infra*). Although **1** is not observed in the final time point for the reaction between **1-CH**_**3**_ and TEMPO, it is observed in the intermediate time points, likely precipitating from solution over the course of the experiment (Figure S40). Addition of TEMPO to *in situ* generated **1-Bu** produced **2** and TEMPO-Bu as the major products in solution (Scheme 4b and Figure S41). Complex **1-Bn** was found to be the most reactive with complete conversion to **1**, **2** and TEMPO-Bn within hours (Scheme 4c and Figure S42). Formation of **2** is observed in the latter two reactions due to TEMPO abstracting hydrogen atoms from the backbone of **1**, a reaction we previously reported to occur at room temperature.^41^

**Scheme 4:**
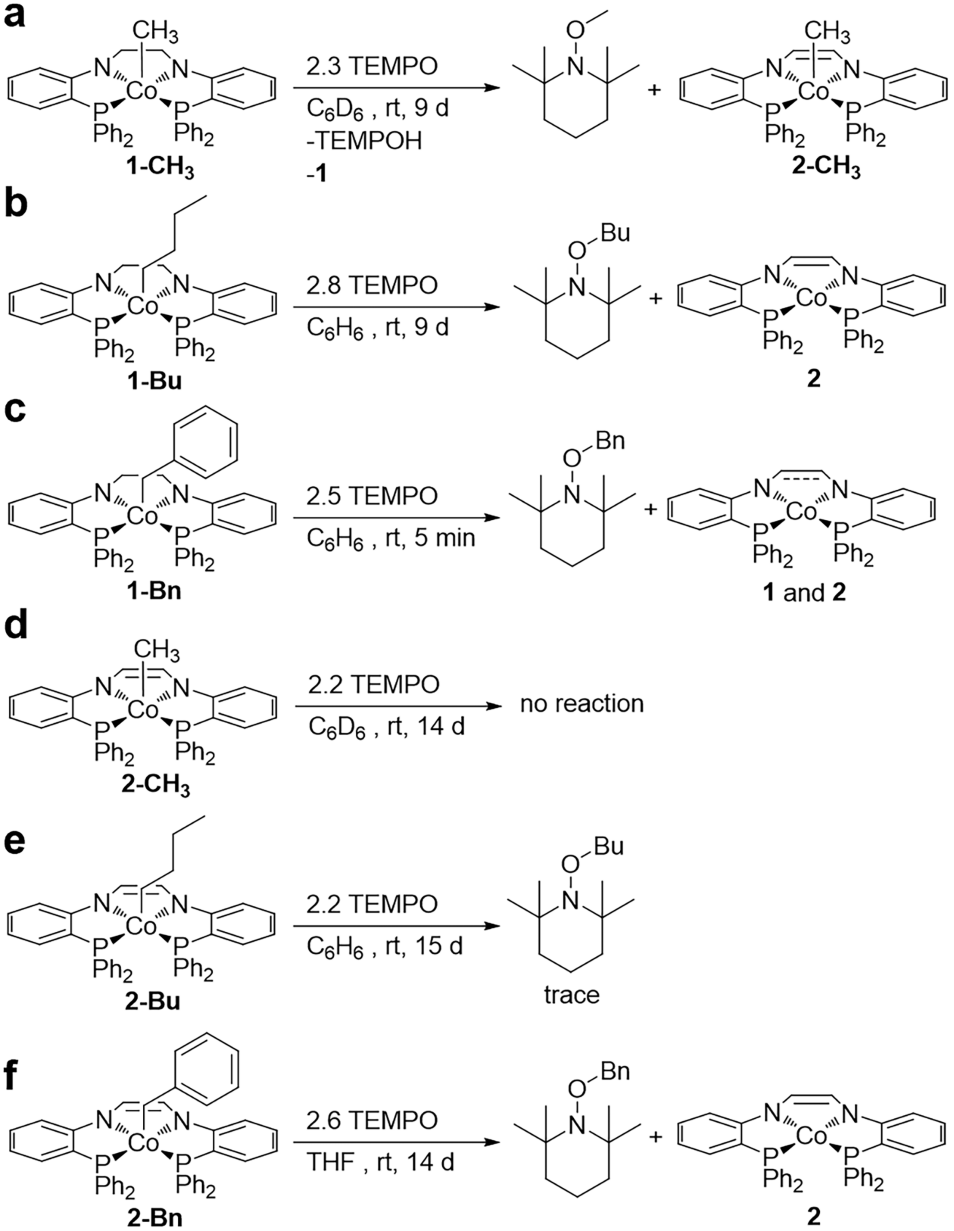
Reactions of Cobalt­(III)-Alkyl Complexes with TEMPO

Contrary to the **1-R** derivatives, the **2-R** cobalt­(III)-alkyl complexes displayed sluggish reactivity with TEMPO at room temperature. Addition of TEMPO to **2-CH**_**3**_ showed no signs of reactivity at room temperature over 14 days, while very minimal conversion (<5%) of **2-Bu** to TEMPO-Bu was observed after 15 days (Scheme 4d,4e, Figures S43 and S44). **2-Bn** was the most reactive of the **2-R** derivatives, with partial conversion to **2** and TEMPO-Bn observed after 14 days (Scheme 4f and Figure S45).

### DFT Calculations

Computational methods were used to compare the Co–C bond dissociation enthalpies (BDEs) of **1-CH**_**3**_, **1-Bu**, **1-Bn**, **2-CH**_**3**_, **2-Bu**, **2-Bn** and a (Salen)­Co-Bu complex at the TPSS-D3­(BJ)/def2-TZVPP level of theory using the conductor-like polarizable continuum model (CPCM) to model solvation in benzene. Geometries for **1-R**, **2-R**, **1**, **2**, (salen)­Co-Bu, ^•^CH_3_, ^•^CH_2_(CH_2_)_2_CH_3_, and ^•^CH_2_(C_6_H_6_) were optimized followed by numerical frequency calculations (see Experimental Section for details). BDE values were determined by taking the difference in enthalpy between the cobalt­(III)-R complex and the theoretical products of cobalt–carbon bond homolysis (Scheme S2 and Table S6). The **1-R** derivatives were found to have lower cobalt–carbon BDE values than the **2-R** analogues (Table 3). These results are in line with the thermolysis and radical trapping experiments (*vide supra*), where the **1-R** derivatives were found to be more reactive, and therefore have weaker cobalt–carbon bonds than their **2-R** counterparts. Similarly, the DFT results also corroborate the experimental differences observed when changing the alkyl substituent and keeping the equatorial ligand the same. For example, the cobalt methyl derivatives have higher cobalt–carbon BDE values than the cobalt benzyl derivatives with the same equatorial ligand (Table 3). The calculated BDE for a (Salen)­Co-Bu complex was lower than the **2-R**, **1-CH**_**3**_, and **1-Bu** derivatives and roughly equal to the calculated BDE for the **1-Bn** molecule (Table 3). This suggests the cobalt–carbon bonds for the complexes in this manuscript are stronger than previously reported cobalt­(III)-alkyl complexes (see Discussion section for a complete explanation).

**Table 3:** DFT Calculated Cobalt–Carbon Bond Dissociation Enthalpy (BDE) Values

Cmpd	Calculated BDE (kcal/mol)	Cmpd	Calculated BDE (kcal/mol)
**1-CH**_**3**_	39.3	**2-CH**_**3**_	41.5
**1-Bu**	40.0	**2-Bu**	42.7
**1-Bn**	34.7	**2-Bn**	39.4
**(Salen)**Co-Bu	34.4		

### Eyring Plot

Radical trapping experiments with TEMPO at multiple temperatures have been shown to be an effective method for determining cobalt–carbon bond dissociation enthalpy (*D*_Co‑R_).^1,45^ Complex **2-Bn** was selected as a representative molecule and the pseudo first-order rate constant for the reaction between **2-Bn** and 20 equiv of TEMPO was determined at temperatures ranging from 34.4–58 °C (Figure S47). An Eyring plot was constructed from the observed rate constants (Figure 6) and used to calculate Δ*H*_1_^‡^ = 41.3 kcal/mol, Δ*S* ^‡^ = 51.1 cal/mol·K, and Δ*G* ^‡^ = 26.0 kcal/mol (at 298 K). The recombination of cobalt­(II) and R^•^ can be assumed to be diffusion controlled (Δ*H*_–1_^‡^ ∼ 2 kcal/mol), therefore, *D*_Co‑R_ ∼ Δ*H*_1_^‡^ – 2 kcal/mol.^44^ Using this assumption, the *D*_Co‑R_ for **2-Bn** is 39.3 kcal/mol, which is larger than previously reported cobalt­(III)-benzyl complexes.^33,38,45^ This is in agreement with the calculated cobalt–carbon BDE of 39.4 kcal/mol for **2-Bn** (Table 3).

**Figure 6:**
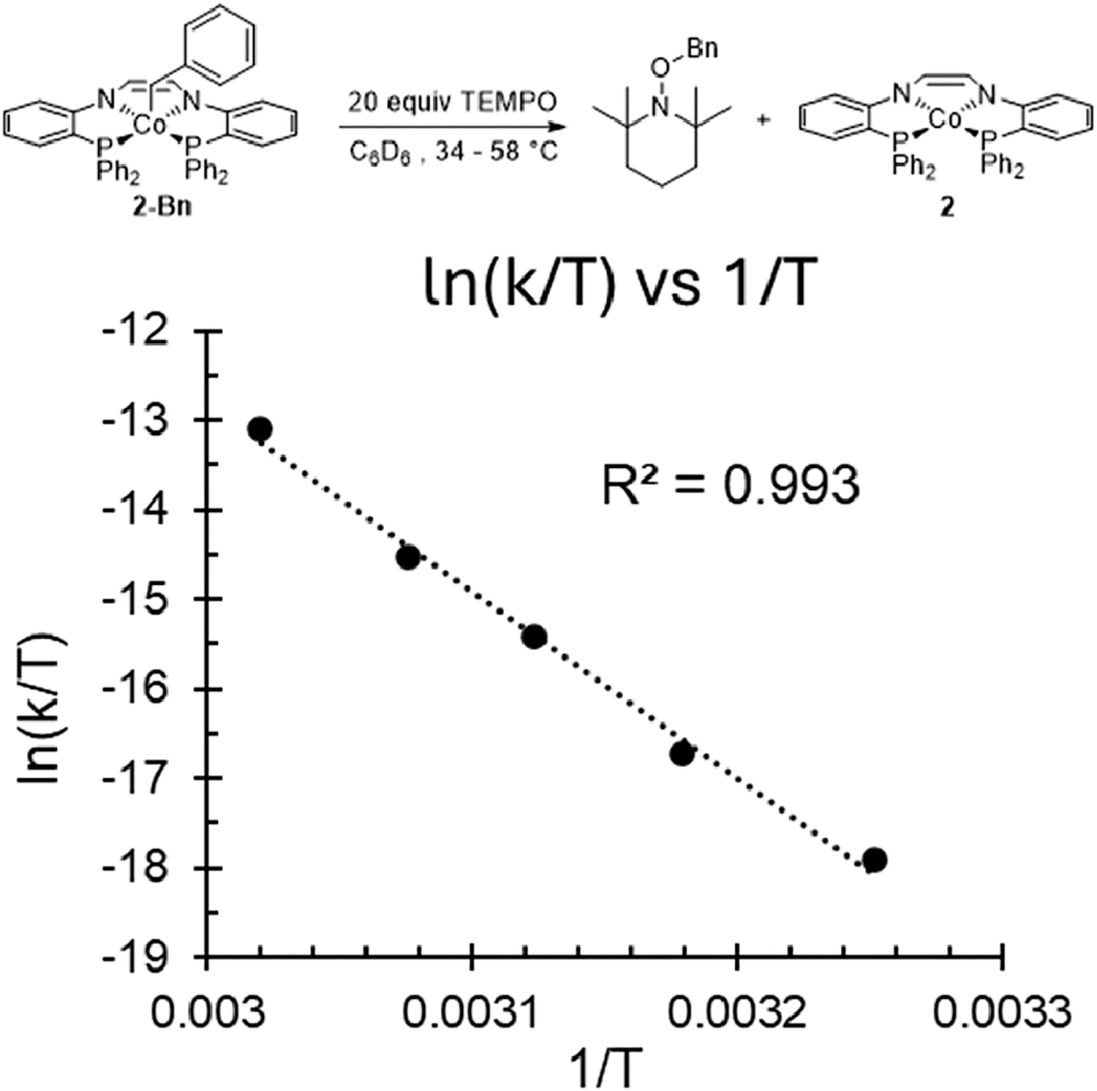
Eyring plot for the reaction between **2-Bn** and TEMPO.

### Catalytic Investigations

Cobalt­(III)-alkyl complexes have been invoked as key sources of alkyl radicals in a variety of catalytic transformations, including radical hydrofunctionalization reactions, radical polar crossover catalysis, and radical polymerization reactions.^27,43,46–48^ Cobalt-catalyzed radical Heck-type cross-coupling reactions of alkyl halides and olefins also typically require the homolysis of cobalt–carbon bonds as part of their mechanism.^28,49^ With this in mind, we sought to investigate the radical Heck-type cross-coupling reactivity of **1** and **2**. Our investigations into the cobalt- carbon bond strength for **1-R** and **2-R** derivatives found **1-Bn** to have the weakest cobalt–carbon bond. Therefore, we investigated the radical Heck-type cross-coupling of BnCl with styrene using **1** as a catalyst.

Initial catalytic investigations began with the combination of styrene and BnCl at 50 °C in THF for 2 h with 2 equiv Zn as a stoichiometric reductant and 5 mol % catalyst loading of **1** (with respect to BnCl) at a concentration of 5 mM (Table 4). We first optimized the equivalents of styrene for the reaction and found that increasing the concentration of styrene increases both the yield and selectivity of the reaction for the cross-coupled product (**A**) over the radical homocoupling product (**B**) (Table 4, entries 1–4). We note an absence of bibenzyl (product **B**) during the homolysis experiment of **1-Bn** and attribute the discrepancy in products to the different instantaneous concentrations of benzyl radicals in solution (see Supporting Information for a detailed explanation). Only slight increases in yield were observed when comparing 10 and 20 equiv of styrene, therefore, we used 10 equiv of styrene for the remaining optimization reactions.

**Table 4:**
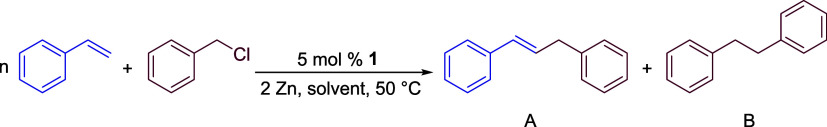
Catalyst Optimization for Radical Heck-Type Cross-Coupling Using **1** as a Catalyst

Entry	Conc. (mM)	Styrene (equiv)	Time	Solvent	Yield, A (%)^a^	Yield, B (%)^a^
**1**	5	1	2 h	THF	13.3	15.5
**2**	5	5	2 h	THF	21.7	4.0
**3**	5	10	2 h	THF	38.9	0.9
**4**	5	20	2 h	THF	40.4	0.5
**5** ^b^	1	10	24 h	THF	42.8	2.9
**6**	9	10	2 h	THF	35.2	1.3
**7** ^b^	5	10	24 h	Toluene	11.2	6.6
**8**	5	10	24 h	MeCN	6.5	6.0
**9**	5	10	4 h	Dioxane	23.4	0.8
**10** ^c^	5	10	2 h	THF	20.1	1.2
**11** ^d^	5	10	2 h	THF	34.9	1.9

a Yields were determined via GC/MS and referenced to an internal standard (trimethoxybenzene).

b No major product detected after 2 h.

c Reaction at 40 °C.

d Reaction at 60 °C.

Increasing the concentration of the catalyst (and the substrates) results in a lower yield of the desired products, while decreasing the concentration improves the yield but requires a longer reaction time (Table 4, entries 5 and 6). The solvent also impacts the reaction, with toluene and MeCN giving lower yields and decreased selectivity for the cross-coupled product (Table 4, entries 7 and 8). Performing the catalytic reaction in dioxane resulted in a lower yield than THF, but with similar selectivity (Table 4, entry 9). Increasing or decreasing the temperature of the reaction also resulted in lower yields (Table 4, entries 10 and 11). When Mg was used as an additive instead of Zn, the selectivity for the desired cross-coupled product eroded significantly (Table 5, entry 1). Increasing the equivalents of Zn or adding one equivalent NaCl as an additive did not improve the yield of the reaction (Table 5, entries 2 and 3). Importantly, control reactions revealed that both Zn and **1** are required for the catalytic reaction to turn over (Table 5, entries 4 and 5).

**Table 5:**
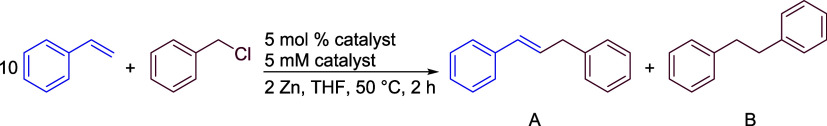
Screening of Additives, Catalyst Identity, and Control Reactions for Radical Heck-Type Cross-Coupling

Entry	Deviation from optimized conditions	Yield, A (%)^a^	Yield, B (%)^a^
**1**	2 equiv Mg instead of Zn	9.5	29.5
**2**	5 equiv Zn	31.0	3.9
**3**	1 equiv NaCl as additive	32.1	1.8
**4**	No Zn	<5	n.d.
**5** ^b^	No catalyst	n.d.	n.d.
**6**	**2** as catalyst	5.1	5.2

a Yields were determined via GC/MS and referenced to an internal standard (trimethoxybenzene).

b Reaction ran for 7 d.

Throughout the catalyst optimization experiments, we observed overall yields of less than 50%, which are attributed to the competitive radical-catalyzed polymerization of styrene. Resonances corresponding to polystyrene were detected in the ^1^H NMR spectra post-catalysis (Figure S56). Similarly, polystyrene was observed in the ^1^H NMR spectrum following the reaction of **1-Bn** and 10 equiv of styrene in the absence of BnCl (Figure S55).

Following evaluation of the catalytic activity of **1**, we sought to compare the catalytic activity of **1** and **2** under the optimized conditions (Table 5, entry 6). We observed a significant change in product distribution and yields, with **2** affording much lower overall product yields and showing greater selectivity for the homocoupled product, **B**. The difference in selectivity could be due to the weaker cobalt–carbon bond in **1-Bn** vs **2-Bn** or the reactivity of the generated benzyl radicals with the ligand backbone observed during the thermolysis of the cobalt–carbon bond in **2-Bn**. The different catalytic activity observed for **1** and **2** shows how small changes in the equatorial ligand framework can significantly impact catalytic performance. Investigations of catalytic activity are still ongoing, though these preliminary data suggest our catalyst, **1**, possesses similar reactivity to other cobalt­(III)-alkyl complexes incorporating a tetradentate square planar ligand.^29,30^

The optimized catalytic conditions for our catalyst are similar to previously reported cobalt complexes that catalyze radical Heck-type cross-coupling of styrene with alkyl halides. Typical catalyst loadings for these complexes are between 1–5 mol % cobalt and reaction temperatures ranging from 20–35 °C.^28,49–51^ Although our optimized conditions require higher temperatures, our reaction is complete within 2 h rather than the typical 14–24 h often reported.^28,50,51^ Our use of excess styrene to more efficiently trap alkyl-radicals formed during the reaction is a strategy commonly used by cobalt catalysts for radical Heck-type cross-coupling.^28,29,50^ The primary advantage of our catalyst is the ability to activate an alkyl-chloride for this reaction, as the more labile RBr or RI reagents are more commonly used.^28,29,50,51^ The disadvantages of our catalyst compared to previously reported cobalt complexes are the product distribution and overall yields of the desired product (**A**). Optimized conditions for our catalyst, **1**, provided roughly 40% yield for the desired product (Table 4); while this is comparable to a previously reported CoI_2_(pyr)­(NHC)^51^ catalyst, many catalysts report yields >70% for the desired product.^28,30,49,50^ We also observe undesired polymerization of styrene in our catalytic reaction. Similar undesired polymerization has been observed in a previous report and was successfully suppressed by adding an exogenous proton source.^30^

## Discussion

Our results have established that the identity of the alkyl substituent and equatorial ligand influence the cobalt–carbon bond stability in **1-R** and **2-R** derivatives. The stronger cobalt–carbon bond observed for the Co-CH_3_ molecules compared to the Co-Bu adducts is primarily governed by steric factors and alkyl radical stability. The *E*_p,c_ observed in the CVs of **1-CH**_**3**_ and **1-Bu** (−2.33 V and −2.45 V, respectively) and **2-CH**_**3**_ and **2-Bu** (−2.54 V and −2.58 V, respectively) indicate relatively minor differences in the charge on cobalt for the Co-CH_3_ and Co-Bu derivatives (Figure 4). Although there are minor electronic differences when comparing the Co-CH_3_ and Co-Bu adducts, a pendant butyl group will have a larger steric impact compared to a methyl group. These steric differences are observed when comparing the distance between the cobalt­(III) center and the (P,N,N,P) plane for **2-CH**_**3**_ and **2-Bu** (1.70 Å and 2.08 Å, respectively). Additionally, the butyl radical transiently formed during homolysis of the cobalt–carbon bond is more stable than a methyl radical, thus favoring cobalt–carbon bond homolysis for the Co-Bu adducts compared to the Co-CH_3_ complexes. Therefore, we attribute the decreased cobalt–carbon bond stability observed for the Co-Bu adducts compared to the Co-CH_3_ derivatives to be caused by the increased steric influence and increased stability of the *n-*butyl radical.

Furthermore, even weaker cobalt–carbon bonds are observed for the Co-Bn adducts when compared to the Co-Bu derivatives. While steric differences between butyl and benzyl groups may still play a role, the impacts of their electronic differences are expected to be more significant. The reductive features observed in the CVs of the Co-Bu adducts occur at more reducing potentials than the Co-Bn compounds (*E*_p,c_ = −2.45 V (**1-Bu**), −2.17 V (**1-Bn**); −2.58 V (**2-Bu**), −2.39 V (**2-Bn**), Figure 4). These data suggest the cobalt center in the Co-Bu adducts is more electron rich than the Co-Bn adducts with the same equatorial ligand. Since cobalt–carbon bond homolysis formally results in a one-electron reduction of the Co^III^ center, the increased electron density provided to the cobalt center by the butyl groups results in a more stable cobalt–carbon bond than the more electron-deficient benzyl derivatives; however, the comparative stability of benzyl radicals compared to other alkyl radicals likely contributes to the propensity of the **1-Bn** and **2-Bn** compounds to undergo bond homolysis as well.

The influence of the equatorial ligand on cobalt–carbon bond strength was evaluated by comparing **1-R** and **2-R** analogues with the same alkyl substituent. The cobalt–carbon bonds in the **1-R** molecules were determined to be weaker than their **2-R** counterparts. Computationally, the cobalt–carbon BDEs of the **1-R** analogues were on average 3.2 kcal/mol lower than the unsaturated **2-R** derivatives with the same R group (Table 3). Experimentally, the **1-R** derivatives required milder conditions for thermolytic bond homolysis and displayed faster reactivity toward TEMPO compared to the **2-R** analogues (*vide supra*). We suggest the differences in cobalt–carbon bond stability are a result of the electronic differences between the **1-R** and **2-R** analogues. Comparison of the CVs (Figure 4) indicates the **2-R** complexes are reduced at more negative potentials than **1-R** derivatives with the same alkyl substituent, suggesting the cobalt center in the **2-R** analogues is more electron rich than the **1-R** adducts. More electron density on the cobalt center in the **2-R** cobalt­(III)-alkyl complexes likely stabilizes the cobalt­(III) oxidation state and disfavors Co–C bond homolysis.

The cobalt–carbon bonds in the **1-R** derivatives are shorter than the **2-R** analogues, despite the **2-R** molecules having stronger cobalt–carbon bonds. This observation is due to the flexibility of the **1-R** ligand, which allows further perturbation of the cobalt center out of the PNNP ligand plane, resulting in a shorter cobalt–carbon bond. This perturbation has been described in previously reported five- and six-coordinate cobalt­(III)-alkyl complexes.^5,20^ Additionally, a shorter cobalt–carbon bond is not indicative of a stronger cobalt–carbon bond. Galezowski and Kubicki discussed a cobalt­(III)–CH_3_ phthalocyanine complex with a shorter yet weaker cobalt–carbon bond compared to a cobalt­(III)–CH_3_ octaethylporphinato complex.^5^

The remaining question, however, is why the Co centers in **2-R** are more electron-rich than their saturated backbone analogues **1-R**, a trend that initially seemed counterintuitive. We posit that this phenomenon can be attributed to geometrical differences imparted by the two ligand backbones, which influence the electron density on the cobalt center of these molecules. The saturated ligand increases torsional strain within the backbone compared to the unsaturated ligand framework (see N–C–C-N torsional angles in Table 2). This distortion is apparent when comparing the crystal structures of **1-CH**_**3**_ and **2-CH**_**3**_ or **1-Bn** and **2-Bn** (Figure 3) and results in weaker π-overlap and π-donation from the amide ligands to the cobalt centers in **1-R** compared to their unsaturated analogues. The resulting decrease in electron density destabilizes the Co–C bond.

The phosphine substituents incorporated into the equatorial ligands make **1-R** and **2-R** unique when compared to the equatorial ligands commonly used in cobalamin model compounds (Figure 1). Although the **[PNNP]** ^**2**–^ ligand framework is more electron-rich, the observed properties of **1-R** and **2-R** are consistent with cobalamin model compounds. For example, the primary decomposition pathway of **1-R** and **2-R** proceeded through cobalt–carbon bond homolysis to release alkyl radicals (R^•^) rather than heterolytic bond cleavage pathways that would release alkyl anions (R^–^) or carbocations (R^+^). Regarding cobalt–carbon bond strength, the calculated Δ*H*_1_^‡^ and *D*_Co‑R_ from the radical trapping experiments with TEMPO and **2-Bn**, provides a point of comparison to other cobalamin model compounds. The *D*_Co‑R_ for **2-Bn** is considerably larger than the experimentally determined *D*_Co‑R_ for previously reported cobalt­(III)-benzyl complexes (Table 6).^33,38,45^ Additionally, the calculated cobalt–carbon BDE for **2-Bn** is larger than a (Salen)­Co-Bu derivative (Table 3), in support of the experimental evidence. We suggest this stark contrast is due to stabilization of the cobalt­(III) oxidation state by the electron rich equatorial ligand coordinated to **2-Bn**. Reports by Galezowski^5^ and Sawyer^40^ suggested more basic equatorial ligands increase cobalt–carbon bond stability, in support of our hypothesis.

**Table 6:** Comparison of *D*_Co‑R_ for **2-Bn** to Previously Reported Cobalamin Model Compounds

Molecule	Δ*H*_1_^‡^ (kcal/mol)	*D*_Co‑R_ (kcal/mol)
(pyr)Co(DH)_2_-Bn	—	27.0
(pyr)(salen)Co-Bn	23.6	22.0
(PR_3_)(DH)_2_Co-Bn	24.8 – 32.4	22.8–30.4
(PR_3_)(OEP)Co-Bn	25.8 – 31.6	23.8 – 29.6
**2-Bn** (this work)	41.3	39.3

We have shown that steric factors, electron density on the cobalt, and alkyl-radical stability influence the cobalt–carbon bond strength of derivatives **1-R** and **2-R**. In cases where minimal electronic differences are observed (e.g., Co-CH_3_ vs Co-Bu), steric influence and alkyl-radical stability govern the strength of the cobalt–carbon bond (Co-CH_3_ stronger than Co-Bu). When steric differences are minimized (e.g., Co-Bu vs Co-Bn) the cobalt–carbon bond strength is primarily influenced by the electron density on the cobalt and alkyl-radical stability (Co-Bu stronger than Co-Bn). Since the Co-Bn adducts impart greater steric influence, reduced electron density on the cobalt center, and a more stable alkyl-radical following homolysis compared to the Co-CH_3_ adducts, all three factors contribute to the weaker cobalt–carbon bonds in the Co-Bn derivatives compared to the Co-CH_3_ analogues. When comparing the cobalt–carbon bond stability for the different equatorial ligand frameworks with the same alkyl group (**1-R** vs **2-R**), the torsional strain of the equatorial ligand in the **1-R** derivatives influenced orbital overlap with the amide moieties, reducing electron density on the cobalt, and resulting in a weaker cobalt–carbon bond.

## Conclusion

In conclusion, we report the synthesis of six new cobalt­(III)-alkyl cobalamin model compounds bearing a unique tetradentate equatorial ligand, **[PNNP]** ^**2**–^. Complexes **1-R** and **2-R** were synthesized via oxidative addition of alkyl halides to cobalt­(I) precursors **1-Na** and **2-Na**, respectively. The redox-active ligand of **2** granted access to the one-electron oxidized species **2-PF**_**6**_,^41^ providing an alternate synthetic route to the **2-R** molecules through alkylation with carbanions. All of the **1-R** and **2-R** derivatives crystallize in a square pyramidal geometry, largely dictated by constraints of the tetradentate ligand and consistent with previously reported cobalamin model compounds. Initial investigations into the reactivity of **1-R** and **2-R** with TEMPO and identification of their decomposition products, determined that **1-R** and **2-R** decomposed through cobalt–carbon bond homolysis but with rates that varied as a function of ligand backbone as well as the identity of the alkyl group. The experimental and computational investigations into the cobalt–carbon bond strength of these molecules determined that the more distorted saturated equatorial ligand framework weakens the cobalt–carbon bond by decreasing electron density at the cobalt­(III) center. The benzyl adducts were found to have the weakest cobalt–carbon bonds, and investigations into the radical Heck-type cross-coupling of BnX (X = Cl or Br) with styrene determined **1** to be a more active catalyst compared to **2**.

These investigations provide fundamental insight into the subtle interplay between steric and electronic factors in the design of cobalt catalysts for reactions requiring the generation of alkyl radicals.

## Experimental Section

### General Considerations

Unless otherwise noted, all manipulations were carried out under an inert atmosphere using a nitrogen-filled glovebox or standard Schlenk techniques. Glassware was oven-dried before use. Solvents were degassed by sparging with ultrahigh purity argon and dried via passage through columns of drying agents using a Glass Contours solvent purification system from Pure Process Technologies. Benzene-*d*_*6*_ was degassed via repeated freeze–pump–thaw cycles and dried over 3 Å molecular sieves before use. Tetrahydrofuran-*d*_*8*_ was dried over calcium hydride overnight then vacuum transferred into a sealed Schlenk tube and stored over 3 Å molecular sieves in a nitrogen filled glovebox. (PNCH_2_CH_2_NP)Co (**1**), (PNCHCHNP)Co (**2**), and [(PNCHCHNP)­Co]­[PF_6_] (**2-PF**_**6**_) were prepared according to literature methods.^41^ 2,2,6,6-tetramethyl-1-piperidinyloxy (TEMPO) was purchased commercially then purified by sublimation and stored in a nitrogen-filled glovebox. All other chemicals were purchased from commercial vendors and used without purification. NMR spectra were recorded at ambient temperature on an AVANCE NEO 400 MHz, Bruker AVIII 600 MHz, or Bruker AVANCE III HD Ascend 700 MHz NMR spectrometer. VT NMR experiments employed a Bruker Smart Variable Temperature system with a Bruker Chilling Unit. ^1^H and ^13^C NMR chemical shifts were referenced to residual solvent resonances and are reported in ppm. ^31^P NMR chemical shifts (in ppm) were referenced to 85% H_3_PO_4_ (0 ppm) using an external standard.

### Synthesis of [(PNCH_2_CH_2_NP)­Co]­[Na­(THF)*_n_*] (**1-Na**, *n* = 1–3)

Na (12.1 mg, 0.526 mmol), Hg (2.36 g), a stir bar, and THF (2 mL) were added to a 20 mL scintillation vial with stirring. **1** was dissolved in THF (12 mL) then added to the Na/Hg amalgam resulting in a color change from brown to emerald green. After stirring for 17 h, the reaction solution was filtered through Celite, extracting with additional THF (2 mL). The volatile components were removed from the filtrate in vacuo. The remaining solids were partially dissolved/suspended in C_6_H_6_, placed in the freezer (−35 °C) until frozen then lyophilized. The remaining solids were washed with hexanes (4 × 5 mL) then dried in vacuo producing **1-Na** as a green solid (0.2402 g, 91.4%). Single crystals suitable for X-ray diffraction were grown via the diffusion of pentane vapor into a saturated THF solution of **1-Na** at – 35 °C. The number of THF molecules bound to sodium for **1-Na** varies depending on the sample preparation method. The crystal structure obtained from a crystal grown from a saturated THF solution incorporates three THF molecules bound to the sodium cation and the ^13^C­{^1^H} NMR spectrum displays resonance corresponding to protio-THF that are adjacent to the THF-*d*_*8*_ resonances (Figure S2). Alternatively, elemental analysis suggests that after purification and drying in vacuo only one THF molecule remains (*vide infra*). Therefore, we conclude that samples of **1-Na** crystallized from THF solution incorporate three THF molecules per cobalt complex, while solid-state samples obtained after washing with hydrocarbon solvents and thoroughly drying under vacuum contain only one THF molecule bound to the sodium counterion. ^1^H NMR (700 MHz, THF-*d*_*8*_): δ 8.48 (br), 7.67 (br), 7.21 (s), 6.67 (br), 6.45 (br s), 5.10 (br). ^31^P­{^1^H} NMR (162 MHz, dioxane): δ 59.30 (s). ^13^C­{^1^H} NMR (176 MHz, THF-*d*_*8*_): δ 135.80 (s), 128.77 (s), 128.32 (s), 126.74 (s). Elemental analysis calculated for C_42_H_40_CoN_2_P_2_ONa: 68.85% C, 5.50% H, 3.82% N. Found: 67.06% C, 6.08% H, 4.07% N. It is possible during shipping or handling of the compound water was introduced to the sample. When considering one equivalent of water was introduced to the sample the new calculated EA for C_42_H_42_CoN_2_P_2_O_2_Na is 67.20% C, 5.64% H, 3.73% N.

### Synthesis of [(PNCHCHNP)­Co]­[Na­(THF)*_n_*] (**2-Na**, *n* = 0–3)

Na (5.6 mg, 0.24 mmol), Hg (1.09 g), a stir bar, and THF (2 mL) were added to a 20 mL scintillation vial with stirring. **2** was dissolved in THF (8 mL) then added to the Na/Hg amalgam resulting in a color change from orange to red-brown. After stirring for 17 h, the reaction solution was filtered through Celite, extracting with additional THF (2 mL). The solvent volume of the filtrate was reduced by half (6 mL) in vacuo. Pentane (10 mL) was added to the concentrated solution and the resulting solution was placed in the freezer (−35 °C). After 20 h the suspension was filtered, collecting the solids on a frit and washing with hexanes (3 × 5 mL). The solids were collected then dried in vacuo to afford **2-Na** as a reddish-brown solid (0.0899 g, 89.7%). Single crystals suitable for X-ray diffraction were grown via the diffusion of pentane vapor into a saturated THF solution of **2-Na** at −35 °C. The number of THF molecules incorporated into the structure of **2-Na** varies depending on the sample preparation method. The crystal structure obtained from a crystal grown from a saturated THF solution incorporates three THF molecules bound to the sodium cation. Alternatively, elemental analysis suggests that after purification and drying in vacuo no THF molecules remain (*vide infra*). Therefore, we conclude that samples of **2-Na** crystallized from THF solution incorporate three THF molecules per cobalt complex, while solid-state samples obtained after washing with hydrocarbon solvents and thoroughly drying under vacuum do not contain any THF molecules bound to the sodium countercation. ^1^H NMR (400 MHz, THF-*d*_*8*_): δ 6.93 (t), 6.66 (br), 6.57 (br). ^31^P­{^1^H} NMR (162 MHz, dioxane): δ 60.32 (br s). ^13^C­{^1^H} NMR (176 MHz, THF-*d*_*8*_): δ 132.41 (s), 128.83 (s), 128.27 (s), 127.73 (s), 124.66 (s). Elemental analysis calculated for C_38_H_30_CoN_2_P_2_Na: 69.31% C, 4.59% H, 4.25% N. Found: 69.10% C, 4.84% H, 4.09% N.

### Synthesis of (PNCH_2_CH_2_NP)­Co­(CH_3_) (**1-CH**_**3**_)

**1-Na** (0.0249 g, 0.0340 mmol), a stir bar, and THF (10 mL) were added to a 20 mL scintillation vial. CH_3_I (2.9 μL, 0.047 mmol) was added via glass syringe to THF (2 mL), then the CH_3_I solution was added dropwise to the **1-Na** solution with stirring, resulting in an immediate color change from green to orange. After 35 min, the volatile components were removed in vacuo and the resulting solids were extracted into C_6_H_6_ (5 mL) then filtered through Celite. The filtrate was placed in the freezer (−35 °C) until frozen then lyophilized in vacuo to afford **1-CH**_**3**_ as a pale-orange solid (0.0161 g, 72.5%). Single crystals suitable for X-ray analysis were grown via the diffusion of pentane vapor into a saturated toluene solution of **1-CH**_**3**_ at −35 °C. ^1^H NMR (700 MHz, C_6_D_6_): δ 7.53 (m, *ortho*-PPh_2_, 4H), 7.27 (t, *J* = 7.4 Hz, Ar-*H*, 2H), 7.10 (m, Ar-*H*, 2H), 7.06 (m, *ortho*-PPh_2_, 4H), 6.98–6.92 (m, Ar-*H*, 4H), 6.88 (t, *J* = 7.4 Hz, Ar-*H*, 2H), 6.77 (t, *J* = 7.0 Hz, *meta*-PPh_2_, 4H), 6.73 (t, *J* = 7.0 Hz, *meta*-PPh_2_, 4H), 6.60 (t, *J* = 7.2 Hz, Ar-*H*, 2H), 3.83 (m, NC*H*_2_C*H*_2_N, 2H), 3.55 (m, NC*H*_2_C*H*_2_N, 2H), 0.46 (t, *J* = 7.1 Hz, C*H*_3_, 3H). ^31^P­{^1^H} NMR (162 MHz, C_6_D_6_): δ 51.15 (s). ^13^C­{^1^H} NMR (176 MHz, C_6_D_6_): δ 166.66 (t, *ipso*), 134.27 (s, *ipso*), 133.46 (s), 133.18 (s), 133.07 (s), 132.91 (s), 130.45 (m, *ipso*), 129.75 (s), 129.49 (s), 128.50 (s), 128.35 (s), 121.97 (m, *ipso*), 115.22 (s), 112.74 (s), 54.99 (s, N*C*H_2_ *C*H_2_N), −12.97 (m, Co-*C*H_3_) Repeated attempts to collect elemental analysis data for this compound were unsuccessful.

### Synthesis of (PNCH_2_CH_2_NP)­Co­(*n*C_4_H_9_) (**1-Bu**)

Due to the instability of **1-Bu** all manipulations were performed in a reduced light environment. Additionally, limiting the time **1-Bu** is at room temperature in solution is necessary to obtain analytically pure product due to the facile homolysis of the Co–C bond in this complex. **1-Na** (0.0458 g, 0.0625 mmol), a stir bar, and C_6_H_6_ (8 mL) were added to a 20 mL scintillation vial. With stirring, ^*n*^BuBr (12.8 μL, 0.119 mmol) was added via glass syringe resulting in a color change from green to brown. After 40 min, the reaction solution was filtered through Celite, extracting with additional C_6_H_6_ (6 mL). The filtrate was placed in the freezer (−35 °C) until frozen then lyophilized. The remaining solids were washed with cold (−35 °C) pentane (3 × 5 mL), collecting via filtration over Celite, then the solids were extracted with C_6_H_6_ (8 mL). The filtrate was placed in the freezer (−35 °C) until frozen then lyophilized to afford **1-Bu** as a brown solid (0.0341 g, 78.6%). ^1^H NMR (700 MHz, C_6_D_6_): δ 7.50 (m, *ortho*-PPh_2_, 4H), 7.28 (t, *J* = 7.9 Hz, Ar-*H*, 2H), 7.14 (m, Ar-*H*, 2H), 7.09 (m, *ortho*-PPh_2_, 4H), 7.01 (t, *J* = 7.6 Hz, Ar-*H*, 2H), 6.90 (d, *J* = 8.7 Hz, Ar-*H*, 2H), 6.87 (t, *J* = 7.7 Hz, *meta*-PPh_2_), 6.84 (t, *J* = 7.6 Hz, Ar-*H*, 2H*)*, 6.70 (t, *J* = 7.8 Hz, *meta*-PPh_2_, 4H), 6.61 (t, *J* = 7.4 Hz, Ar-*H*, 2H), 3.88 (m, NC*H*_2_C*H*_2_N, 2H), 3.65 (m, NC*H*_2_C*H*_2_N, 2H), 1.24 (m, Co-CH_2_C*H*_2_CH_2_CH_3_, 2H), 1.15 (m, Co–C*H*_2_CH_2_CH_2_CH_3_, 2H), 0.65 (m, Co-CH_2_CH_2_C*H*_2_CH_3_, 2H), 0.42 (t, *J* = 7.4 Hz, Co-CH_2_CH_2_CH_2_C*H*_3_, 3H). ^31^P­{^1^H} NMR (162 MHz, C_6_D_6_): δ 52.33 (s). ^13^C­{^1^H} NMR (176 MHz, C_6_D_6_): δ 166.32 (t, *J* = 12.8 Hz, *ipso*), 133.58 (t, *J* = 4.2 Hz, *ortho*-PPh_2_), 133.22 (s), 133.18 (m, *ipso*), 132.94–132.88 (m, overlapping), 130.40 (m, *ipso*), 129.63 (s), 129.60 (s), 128.35 (s), 128.32 (s), 121.51 (m, *ipso*), 114.97 (m), 112.51 (t, *J* = 7.2 Hz), 55.16 (s, N*C*H_2_ *C*H_2_N), 37.60 (s, Co-CH_2_ *C*H_2_CH_2_CH_3_), 22.76 (s, Co-CH_2_CH_2_ *C*H_2_CH_3_), 15.44 (t, *J* = 15.6 Hz, Co-*C*H_2_CH_2_CH_2_CH_3_), 13.30 (s, Co-CH_2_CH_2_CH_2_ *C*H_3_). Due to the instability of **1-Bu** in light and at room temperature, elemental analysis data could not be obtained.

### Synthesis of (PNCH_2_CH_2_NP)­Co­(CH_2_(C_6_H_5_)) (**1-Bn**)

Due to the instability of **1-Bn** all manipulations were performed in a reduced light environment. Additionally, limiting the time **1-Bn** is at room temperature in solution is necessary to obtain analytically pure product due to the facile homolysis of the Co–C bond in this complex. **1-Na** (0.0383 g, 0.0523 mmol), a stir bar, and C_6_H_6_ (5 mL) were added to a 20 mL scintillation vail. With stirring, ^*n*^BuBr (10.3 μL, 0.0870 mmol) was added via glass syringe, resulting in a color change from green to purple. After 35 min the reaction solution was filtered through Celite, extracting with additional C_6_H_6_ (2 mL). The filtrate was placed in the freezer (−35 °C) until frozen then lyophilized in vacuo. The residual solids were washed with cold (−35 °C) pentane (3 × 5 mL), collecting the solids via filtration over Celite, then the solids were extracted with C_6_H_6_ (15 mL). The filtrate was placed in the freezer (−35 °C) until frozen, then lyophilized to afford **1-Bn** as a green solid (36.8 mg, 96.6%). Single crystals suitable for X-ray diffraction were grown from a saturated pentane solution of **1-Bn** at −35 °C. ^1^H NMR (400 MHz, C_6_D_6_): δ 7.35–7.21 (m, Ar-*H*, 14H), 7.02 (t, *J* = 7.3 Hz, Ar-*H*, 2H), 6.93–6.83 (m, Ar-*H*, 6H), 6.83–6.72 (m, Ar-*H*, 4H) 6.68–6.60 (m, Ar-*H*, 6H), 3.15 (m, NC*H*_2_C*H*_2_N, 2H), 3.02 (m, NC*H*_2_C*H*_2_N, 2H), 2.28 (t, *J* = 7.8 Hz, C*H*_2_(C_6_H_5_), 2H). ^31^P­{^1^H} NMR (162 MHz, C_6_D_6_): δ 50.26 (s). ^13^C­{^1^H} NMR (176 MHz, C_6_D_6_): δ 166.35 (t, *J* = 13.0 Hz, *ipso*), 153.01 (s, *ipso*), 134.13 (s), 133.25 (s), 133.20 (m, *ipso*), 132.68 (m), 132.52 (s), 130.49 (m, *ipso*), 129.93 (s), 129.41 (s), 128.62 (m), 128.59 (s), 128.36 (s), 124.64 (s), 122.36 (m, *ipso*), 115.18 (s), 113.28 (t, *J* = 7.2 Hz), 54.44 (s, N*C*H_2_ *C*H_2_N), 4.35 (t, *J* = 11.7 Hz, *C*H_2_(C_6_H_5_)). *Note:* Only 32/33 protons are accounted for in the ^1^H NMR spectrum. The missing proton was detected under the C_6_D_5_H resonance, determined by the ^13^C–^1^H HSQC spectrum. Similarly, only 19/20 resonances for the carbon atoms of **1-Bn** were observed in the ^13^C­{^1^H} spectrum (Figure S17). The missing carbon was detected under the C_6_D_6_ resonance, determined by the ^13^C–^1^H HSQC NMR spectrum (Figure S18). Due to the instability of **1-Bn** in light and at room temperature, elemental analysis was not attempted.

### Synthesis of (PNCHCHNP)­Co­(CH_3_) (**2-CH**_**3**_)

**2-Na** (0.0456 g, 0.0692 mmol), a stir bar, and THF (5 mL) were added to a 20 mL scintillation vial. With stirring, CH_3_I (6.0 μL, 0.096 mmol) was added via glass syringe, resulting in a color change from orange to green. After 10 min the volatile components were removed in vacuo and the resulting solid was extracted into C_6_H_6_ (6 mL) then filtered through Celite. The filtrate was placed in the freezer (−35 °C) until frozen then lyophilized in vacuo to afford **2-CH**_**3**_ as a brown solid (0.0408 g, 90.7%). *Alternative synthesis:* **2-PF**_**6**_ (41.0 mg, 0.0432 mmol), a stir bar, and THF (4 mL) were added to a 20 mL scintillation vial. With stirring, a 1.6 M solution of CH_3_Li in Et_2_O (24.3 μL, 0.039 mmol) was added via glass syringe, resulting in a color change from purple to green. After 15 min the volatile components were removed in vacuo and the resulting solid was extracted with C_6_H_6_ (4 mL) then filtered through Celite. The volatile components were removed from the filtrate in vacuo and the resulting solid was again extracted with C_6_H_6_ (5 mL), then filtered through Celite. The filtrate was placed in the freezer (−35 °C) until frozen, then lyophilized to afford **2-CH**_**3**_ as a green solid (20.5 mg, 81.0%). Single crystals suitable for X-ray diffraction were grown from the diffusion of pentane vapor into a C_6_D_6_ solution of **2-CH**_**3**_ at room temperature. ^1^H NMR (700 MHz, C_6_D_6_): δ 7.51 (m, Ar-*H*, 2H), 7.50 (s, 2H, NC*H*C*H*N), 7.42 (m, *ortho*-PPh_2_, 4H), 7.14–7.11 (m, Ar-*H*, 8H), 6.96 (t, *J* = 7.5 Hz, Ar-*H*, 2H), 6.85 (t, *J* = 7.4 Hz, Ar-*H*, 2H), 6.79 (t, *J* = 7.5 Hz, *meta*-PPh_2_, 4H), 6.75 (t, *J* = 7.3 Hz, Ar-*H*, 2H), 6.67 (t, *J* = 7.5 Hz, *meta*-PPh_2_, 4H), −0.05 (t, *J* = 5.7 Hz, Co–C*H*_3_, 3H). ^31^P­{^1^H} NMR (162 MHz, C_6_D_6_): δ 55.07 (s). ^13^C­{^1^H} NMR (176 MHz, C_6_D_6_): δ 160.84 (t, *J* = 12.6 Hz, *ipso*), 134.47 (m, *ipso*), 133.60 (t, *J* = 4.2 Hz), 133.09 (t, *J* = 5.9 Hz), 132.79 (s), 132.63 (s), 131.15 (m, *ipso*), 129.57 (s), 129.43 (s), 129.27 (m, *ipso*), 128.38 (s), 128.35 (s), 125.05 (m, N*C*H*C*HN), 120.21 (m), 113.41 (t, *J* = 6.5 Hz), −8.67 (m, Co-*C*H_3_). Elemental analysis calculated for C_39_H_33_CoN_2_P_2_^:^ 72.00% C, 5.11% H, 4.31% N. Found: 72.30% C, 5.01% H, 4.47% N.

### Synthesis of (PNCHCHNP)­Co­(*n*C_4_H_9_) (**2-Bu**)

**2-Na** (0.0605 g, 0.0919 mmol), a stir bar, and THF (5 mL) were added to a 20 mL scintillation vial. With stirring ^*n*^BuBr (8.1 μL, 0.075 mmol) was added via glass syringe, resulting in a color change from orange to green. After 35 min the volatile components were removed in vacuo and the resulting solid was extracted into C_6_H_6_ (5 mL) then filtered through Celite. The filtrate was placed in the freezer (−35 °C) until frozen then lyophilized in vacuo to afford **2-Bu** as a green solid (0.0433 g, 68.1%). *Alternative synthesis:* **2-PF**_**6**_ (37.6 mg, 0.0396 mmol), a stir bar, and THF (3 mL) were added to a 20 mL scintillation vial. With stirring, a 1.6 M solution of ^*n*^BuLi in hexanes (22.3 μL, 0.036 mmol) was added via glass syringe resulting in a color change from purple to green. After 20 min, the solvent was removed in vacuo and the residual solids were extracted with C_6_H_6_ (6 mL), then filtered through Celite. The volatile components were removed from the filtrate and the residual solids were again extracted with C_6_H_6_ (6 mL), then filtered through Celite. The filtrate was placed in the freezer (−35 °C) until frozen then lyophilized to afford **2-Bu** as a green solid (17.9 mg, 72.5%). Single crystals suitable for X-ray diffraction were grown from the diffusion of pentane vapor into a C_6_H_6_ solution of **2-Bu** at room temperature. ^1^H NMR (400 MHz, C_6_D_6_): δ 7.51–7.42 (m, Ar-*H*, 8H), 7.21–7.17 (m, Ar-*H*, 2H), 7.14 (m, Ar-*H*, 2H), 7.10 (m, Ar-*H*, 4H), 7.03 (t, *J* = 7.3 Hz, Ar-*H*, 2H), 6.89 (t, *J* = 7.6 Hz, *meta*-PPh_2_, 4H), 6.83 (t, *J* = 7.4 Hz, Ar-*H*, 2H), 6.75 (t, *J* = 7.4 Hz, Ar-*H*, 2H), 6.67 (t, *J* = 7.2 Hz, *meta*-PPh_2_, 4H), 0.84 (m, Co-CH_2_C*H*_2_C*H*_2_CH_3_, 4H), 0.65 (m, Co–C*H*_2_CH_2_CH_2_CH_3_, 2H), 0.46 (m, Co-CH_2_CH_2_CH_2_C*H*_3_, 3H). ^31^P­{^1^H} NMR (162 MHz, C_6_D_6_): δ 55.53 (s). ^13^C­{^1^H} NMR (176 MHz, C_6_D_6_): δ 160.74 (t, *J* = 12.4 Hz), 134.46 (m, *ipso*), 133.38 (m), 133.10 (t, *J* = 5.9 Hz), 132.77 (s). 132.66 (s), 131.03 (m, *ipso*), 129.64 (s), 129.46 (s), 128.56 (m, *ipso*), 128.35 (s), 124.00 (s, N*C*H*C*HN), 119.95 (s), 113.41 (t, *J* = 6.5 Hz), 32.90 (s, Co-CH_2_ *C*H_2_ *C*H_2_CH_3_), 25.27 (s, Co-CH_2_ *C*H_2_ *C*H_2_CH_3_), 14.76 (m, Co-*C*H_2_CH_2_CH_2_CH_3_), 13.40 (s, Co-CH_2_CH_2_CH_2_ *C*H_3_). *Note:* Only 18/19 ^13^C­{^1^H} resonances were observed; the missing resonance was detected under the C_6_D_6_ signal, determined by the ^13^C–^1^H HSQC NMR spectrum (Figure S25). Elemental analysis calculated for C_42_H_39_CoN_2_P_2_: 72.83% C, 5.68% H, 4.04% N. Found: 69.15% C, 5.35% H, 3.69% N. Repeated attempts to collect more satisfactory elemental analysis data for this compound were unsuccessful.

### Synthesis of (PNCHCHNP)­Co­(CH_2_(C_6_H_5_)) (**2-Bn**)

**2-Na** (0.0360 g, 0.0546 mmol), a stir bar, and C_6_H_6_ (5 mL) were added to a 20 mL scintillation vial. With stirring BnBr (9.7 μL, 0.082 mmol) was added via glass syringe resulting in a color change from orange to green. After 40 min, the reaction solution was filtered through Celite, extracting with additional C_6_H_6_ (2 mL). The filtrate was placed in the freezer (−35 °C) until frozen, then lyophilized. The solids were dissolved in PhF (2 mL) and pentane vapor was diffused into the PhF solution in the freezer (−35 °C). After 6 days the mother liquor was filtered and the volatile components were removed from the filtrate affording **2-Bn** as a green solid (0.0292 g, 73.6%). *Alternative synthesis:* **2-PF**_**6**_ (37.1 mg, 0.0391 mmol), a stir bar, and THF (3 mL) were added to a 20 mL scintillation vial. With stirring, a 2.0 M solution of BnMgCl in THF (17.6 μL, 0.035 mmol) was added via glass syringe, resulting in a color change from purple to green. After 20 min, the solvent was removed in vacuo and the residual solids were extracted with C_6_H_6_ (5 mL), then filtered through Celite. The volatile components were removed from the filtrate in vacuo and the residual solids were extracted with dioxane (10 mL), then filtered through Celite. The volatile components were removed from the filtrate in vacuo and the residual solids were extracted with C_6_H_6_ (5 mL), then filtered through Celite. The filtrate was placed in the freezer (−35 °C) until frozen, then lyophilized to afford **2-Bn** as a green solid (17.6 mg, 69.0%). Single crystals suitable for X-ray diffraction were grown from the diffusion of pentane vapor into a saturated PhF solution of **2-Bn** at −35 °C. ^1^H NMR (700 MHz, C_6_D_6_): δ 7.30–7.24 (m, Ar-*H*, 8H), 7.22 (m, *ortho*-PPh_2_, 4H), 7.14 (s, NC*H*C*H*N, 2H), 7.10 (t, *J* = 7.7 Hz, Ar-*H*, 2H), 7.05 (t, *J* = 7.5 Hz, Ar-*H*, 2H), 7.00 (t, *J* = 7.5 Hz, Ar-*H*, 2H), 6.93–6.87 (m, Ar-*H*, 5H), 6.84 (t, *J* = 7.3 Hz, Ar-*H*, 2H), 6.77 (t, *J* = 7.4 Hz, Ar-*H*, 2H), 6.62–6.55 (m, Ar-*H*, 6H), 1.97 (t, *J* = 5.8 Hz, Co–C*H*_2_(C_6_H_5_), 2H). ^31^P­{^1^H} NMR (162 MHz, C_6_D_6_): δ 54.13 (s). ^13^C­{^1^H} NMR (176 MHz, C_6_D_6_): δ 160.68 (t, *J* = 12.7 Hz, *ipso*), 149.28 (s, *ipso*), 134.96 (m, *ipso*), 134.10 (t, *J* = 4.2 Hz), 132.75 (m), 132.73 (s), 132.35 (s), 131.19 (m, *ipso*), 130.05 (m, *ipso*), 129.84 (s), 129.26 (s), 128.56 (t, *J* = 3.9 Hz), 128.35 (s), 126.85 (s), 126.00 (m), 125.63 (s), 122.02 (s), 120.74 (m), 114.45 (t, *J* = 6.2 Hz), 11.02 (m, Co-*C*H_2_(C_6_H_5_)). Elemental analysis calculated for C_45_H_37_CoN_2_P_2_: 74.38% C, 5.13% H, 3.86% N. Found: 73.30% C, 5.00% H, 3.66% N.

### Thermolysis Experiments General Procedure

2–4 mg of the cobalt-alkyl complex (**1-CH**_**3**_, **1-Bu**, **1-Bn**, **2-CH**_**3**_, **2-Bu**, or **2-Bn**) was dissolved in C_6_D_6_ (1 mL) then filtered into a J. Young tube through a pipet packed with glass microfiber filter paper. The J. Young tube was left at room temperature or heated and ^1^H NMR spectra were collected periodically to monitor the formation of decomposition products. All manipulations were performed in reduced light or dark conditions. See Figures S29–S39 for details.

### Reaction of **1-Bn** with TEMPO

For this experiment, **1-Bn** was generated *in situ* owing to its thermal instability. **1-Na** (22.8 mg, 0.0241 mmol) was dissolved in C_6_H_6_ (6 mL) in a 20 mL scintillation vial, then BnBr (3.3 μL, 0.031 mmol) was added via glass syringe with stirring in the dark. After 40 min, the reaction solution was filtered through a pad of Celite into a new 20 mL scintillation vial in the dark. TEMPO (9.3 mg, 0.060 mmol) was dissolved in C_6_H_6_ (1 mL) and added with stirring, no longer in reduced light conditions. The volatile components were removed in vacuo from an aliquot (0.5 mL) that was collected after 5 min. The remaining solid was dissolved in C_6_D_6_ for ^1^H NMR analysis (Figure S42). No further aliquots were collected, as the reaction was complete after the first time point.

### Reaction of **1-CH**_**3**_ with TEMPO

**1-CH**_**3**_ (6.0 mg, 0.0092 mmol) was dissolved in C_6_D_6_ (1 mL) then added to a shell vial containing TEMPO (3.3 mg, 0.021 mmol) with stirring. A ^1^H NMR spectrum was collected after 2 h, 26 h, 2 d, and 9 d (Figure S40). The solution changed color from orange to brown-green over the course of the experiment.

### Reaction of **1-Bu** with TEMPO

For this experiment, **1-Bu** was generated *in situ* owing to its thermal instability. **1-Na** (24.9 mg, 0.0263 mmol) was dissolved in C_6_H_6_ (6 mL) in a 20 mL scintillation vial, then ^*n*^BuBr (3.3 μL, 0.031 mmol) was added via glass syringe with stirring in the dark. After 40 min, the reaction solution was filtered through a pad of Celite into a new 20 mL scintillation vial in the dark. TEMPO (11.5 mg, 0.0736 mmol) was dissolved in C_6_H_6_ (1 mL) and added with stirring, no longer in reduced light conditions. The volatile components were removed in vacuo from an aliquot (0.5 mL) that was collected after 5 min. The remaining solids were dissolved in 0.75 mL C_6_D_6_ for ^1^H NMR analysis. This procedure was repeated with aliquots collected after 6 h, 24 h, 3 d, and 9 d (Figure S41). The solution changed color from orange to red over the course of the experiment.

### Reaction of **2-Bn** with TEMPO

TEMPO (7.3 mg, 0.47 mmol) was dissolved in THF (3 mL) then added to a 20 mL scintillation vial containing **2-Bn** (13.2 mg, 0.0182 mmol). After 24 h, the volatile components were removed in vacuo from a collected aliquot (0.5 mL). The remaining solid was dissolved in 0.75 mL C_6_D_6_ for ^1^H NMR analysis. This procedure was repeated after 3, 7, and 14 d (Figure S45). The solution changed from green to red-orange over the course of the experiment.

### Reaction of **2-Bu** with TEMPO

TEMPO (5.4 mg, 0.035 mmol) was dissolved in C_6_H_6_ (3 mL) and added to a 20 mL scintillation vial with **2-Bu** (10.8 mg, 0.0156 mmol) with stirring, resulting in a brown-green solution. The volatile components were removed in vacuo from an aliquot (0.5 mL) that was collected after 19 h. The remaining solid was dissolved in 0.75 mL C_6_D_6_ for ^1^H NMR analysis. This procedure was repeated after 6, 7, 8, and 15 d (Figure S44). The solution did not change color significantly over the course of the experiment.

### Reaction of **2-CH**_**3**_ with TEMPO

**2-CH**_**3**_ (13.7 mg, 0.0211 mmol) was dissolved in C_6_D_6_ (1 mL) then added to a shell vial containing TEMPO (7.3 mg, 0.047 mmol) with stirring, resulting in a green solution. A ^1^H NMR spectrum was collected after 19 h, 6 d, 7 d, and 14 d. After 14 days the reaction did not change color and no reaction was observed in any of the ^1^H NMR spectra collected. The ^1^H NMR spectrum after 14 days is displayed as a representative example in Figure S43.

### Eyring Plot Experiments General Procedure

The pseudo first-order rate constant for the reaction between **2-Bn** and ∼20 equiv of TEMPO was determined at five different temperatures ranging from 34–58 °C. The concentration of **2-Bn** was monitored using quantitative ^1^H NMR spectroscopy and hexamethylbenzene as an internal standard. Representative ^1^H NMR spectra are shown in Figure S48. Graphs of [**2-Bn**] vs time and ln­[**2-Bn**] vs time at each temperature are displayed in Figures S46 and S47. Specific experimental details for the reaction at each temperature are provided in the SI. All manipulations were performed in reduced light or dark conditions to eliminate photolytically induced homolysis as a variable for these experiments.

### Standard Catalytic Procedure

**1** (5 mol %), a stir bar, zinc powder (2 equiv), THF (5 mM in catalyst), styrene (10 equiv), and benzyl chloride (1 equiv) were added to a 20 mL scintillation vial with stirring and placed on a hot plate (50 °C). Aliquots were collected via micropipette and yields were quantified by GC-MS.

### X-ray Crystallography Procedures

All operations were performed on a Bruker Kappa Photon II CPAD diffractometer, using graphite-monochromate Mo Kα radiation. All diffractometer manipulations, including data collection, integration, scaling, and absorption corrections, were carried out using the Bruker Apex2 or Apex3 software suite. Fully labeled diagrams are shown on pages S63–76 while crystal data and structure refinement details can be found in Tables S1–S4.

### Cyclic Voltammetry

Cyclic voltammetry measurements were conducted in a nitrogen filled glovebox using a 0.1 M [*^n^* Bu_4_N]­[PF_6_] electrolyte solution in THF using a CHI 620 E potentiostat (CH instruments Inc., Austin TX). A glassy carbon electrode was used for the working electrode, a platinum wire was used for the counter electrode, and a Ag/Ag^+^ reference electrode was used with 0.01 M AgNO_3_/0.1 M [*^n^* Bu_4_N]­[PF_6_] as the reference solution.

### Computational Details

The ORCA 5.0.4 software package^52^ was used for all computations. Geometry optimizations and single-point numerical frequency calculations were performed at the TPSS^53^/def2-TZVPP^54,55^ level of theory using the conductor-like polarizable continuum model (CPCM)^56^ to model solvation in benzene, with the Becke-Johnson damping (D3BJ)^57,58^ dispersion correction. Crystallographic coordinates were used as the starting geometries for **1**, **2**, **1-CH**_**3**_, **1-Bn**, **2-CH**_**3**_, **2-Bu**, and **2-Bn**, following the removal of the solvent molecule(s). The coordinates for the starting geometry of **1-Bu** were derived from **1-CH**_**3**_ by removing the methyl group and adding a butyl group bound to the cobalt center. The starting geometries for (Salen)­Co and (Salen)­Co-Bu were obtained from the xyz coordinates of a previous reference.^39^ The carbon-centered radicals (CH_3_^•^, Bu^•^, and Bn^•^) were constructed in GaussView^59^ and the resultant coordinates were used as the starting geometry for the computations. Numerical frequency calculations were performed on all optimized geometries to ensure the absence of significant imaginary frequencies (>100 cm^–1^) and provided the energetic data for the cobalt–carbon bond dissociation calculations.

## Supplementary Material





## Notes

Spectroscopic characterization data for compounds **1-R** and **2-R**, spectral data and additional experimental details for thermolysis experiments, reactions with TEMPO, Eyring plot analysis (**2-Bn**), and catalysis experiments, computational details, X-ray crystallography details for **1-R** and **2-R** (PDF)
Cartesian coordinates for all DFT-optimized structures (XYZ)
