# Supplementary material for: Synthesis, Characterization, and Evaluation of the Cobalt–Carbon Bond Strength of (PNNP)CoIII-R Complexes
Source: Organometallics. 2026 Apr 2;45(7):845–59. doi: 10.1021/acs.organomet.6c00008 (PMC13081110; doi:10.1021/acs.organomet.6c00008)
Supplement: Supplementary file 1 [file om6c00008_si_001.pdf]

SUPPORTING INFORMATION

Synthesis, Characterization, and Evaluation of the Cobalt-Carbon Bond Strength of (PNNP)Co^{III}-R Complexes

Justin D. Miller, Mitchell M. Walsh, Curtis E. Moore, Christine M. Thomas*

Department of Chemistry and Biochemistry, The Ohio State University, Columbus, Ohio 43210, United States

thomasc@chemistry.ohio-state.edu

Table of Contents

NMR Spectroscopy of 1-R and 2-R complexes 2

Thermolysis Experiments 20

Preliminary reactions of 1-R and 2-R with TEMPO 31

Eyring Plot Experiments 37

Catalysis 41

GC Traces for Catalytic Trials 46

Crystal Structures 63

Cyclic Voltammetry 81

Computational Studies 82

NMR Spectroscopy of 1-R and 2-R complexes

Figure S1. ^{1}H NMR spectrum of **1-Na** (700 MHz, THF-d_8). Residual benzene is denoted by a * and free THF is denoted by #.

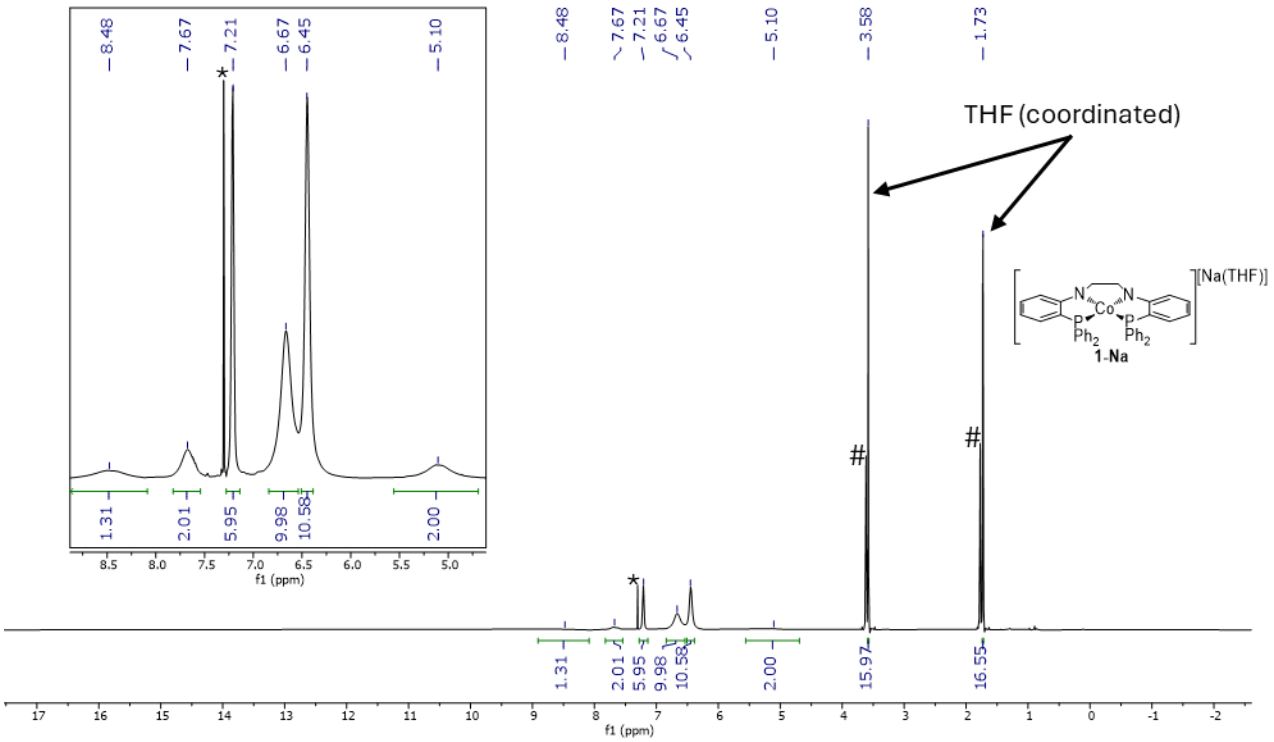

Figure S2. $^{13}C\{^{1}H\}$ NMR spectrum of **1-Na** (176 MHz, THF-d_8). THF-d_8 is denoted by # and THF is denoted by a *.

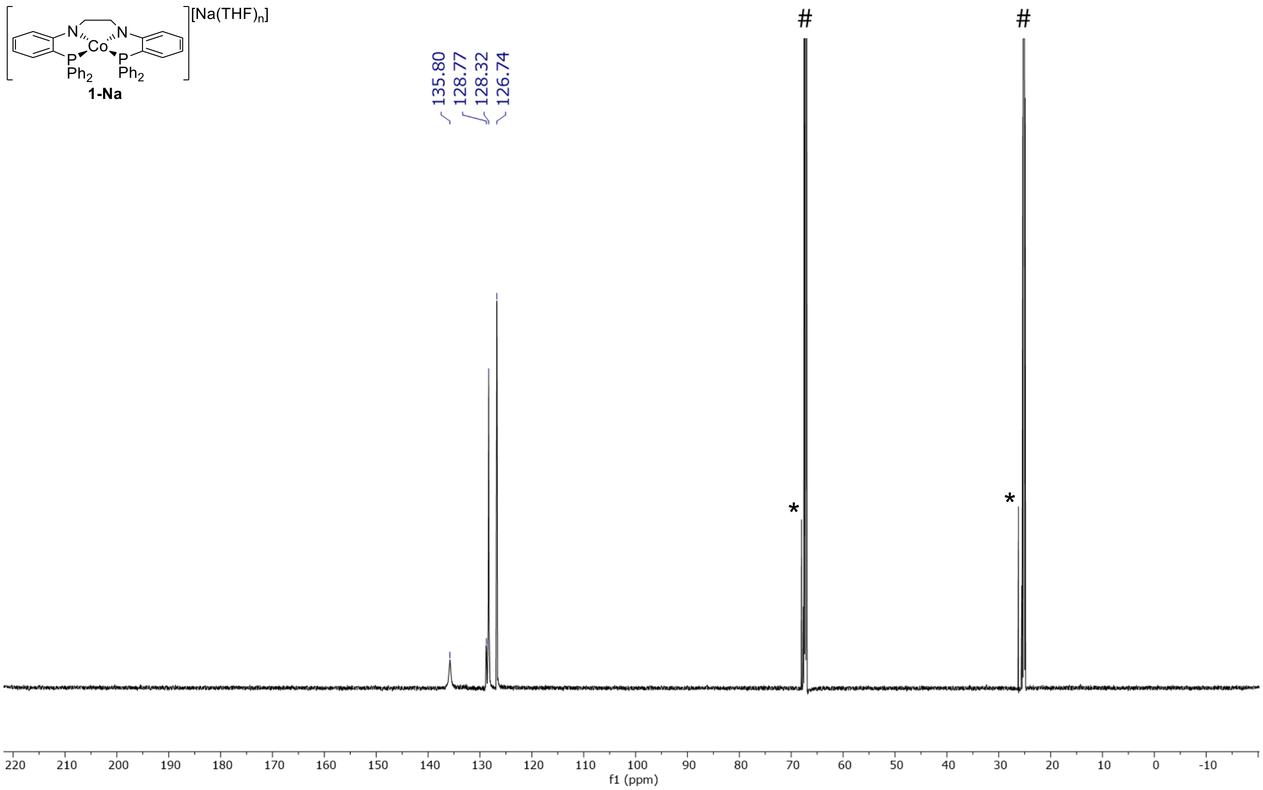

Figure S3. $^{31}P\{^{1}H\}$ NMR spectrum of **1-Na** (162 MHz, dioxane).

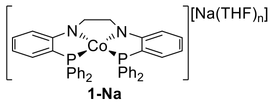

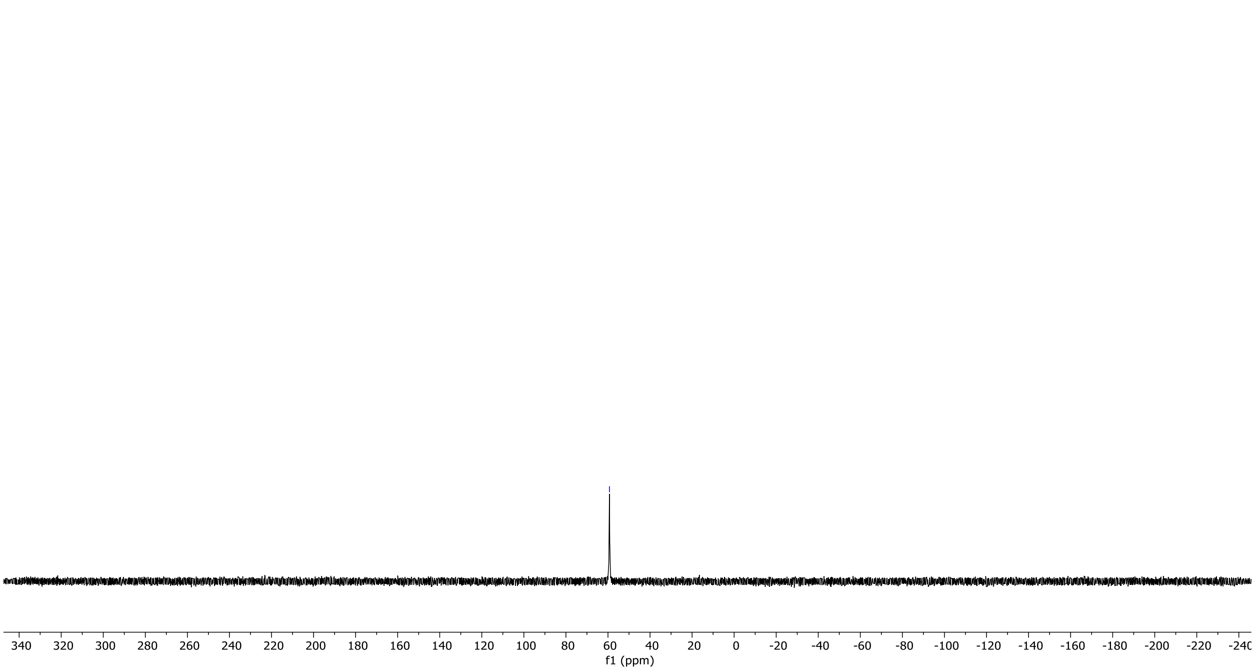

Figure S4. ^{1}H NMR spectrum of **2-Na** (400 MHz, THF-d_8). Residual benzene is denoted by a * and THF is denoted by #.

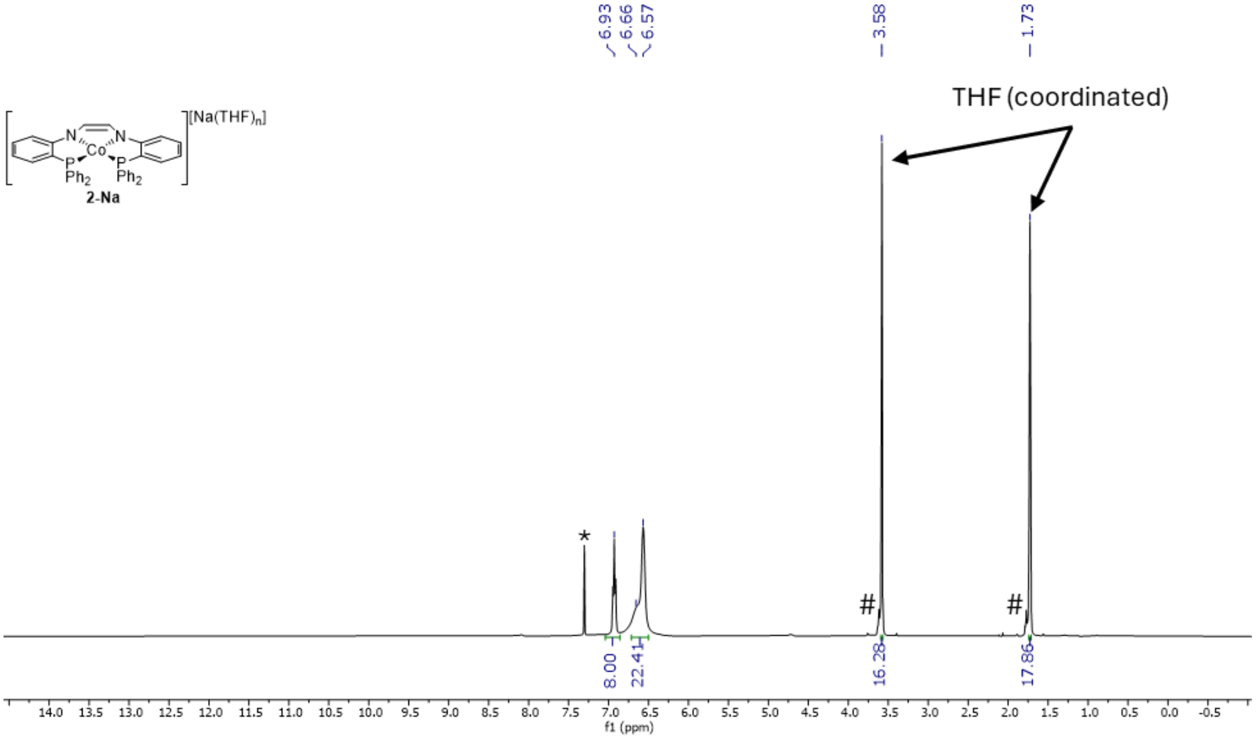

Figure S5. $^{13}C\{^{1}H\}$ NMR spectrum of **2-Na** (176 MHz, THF-d_8). THF is denoted by #.

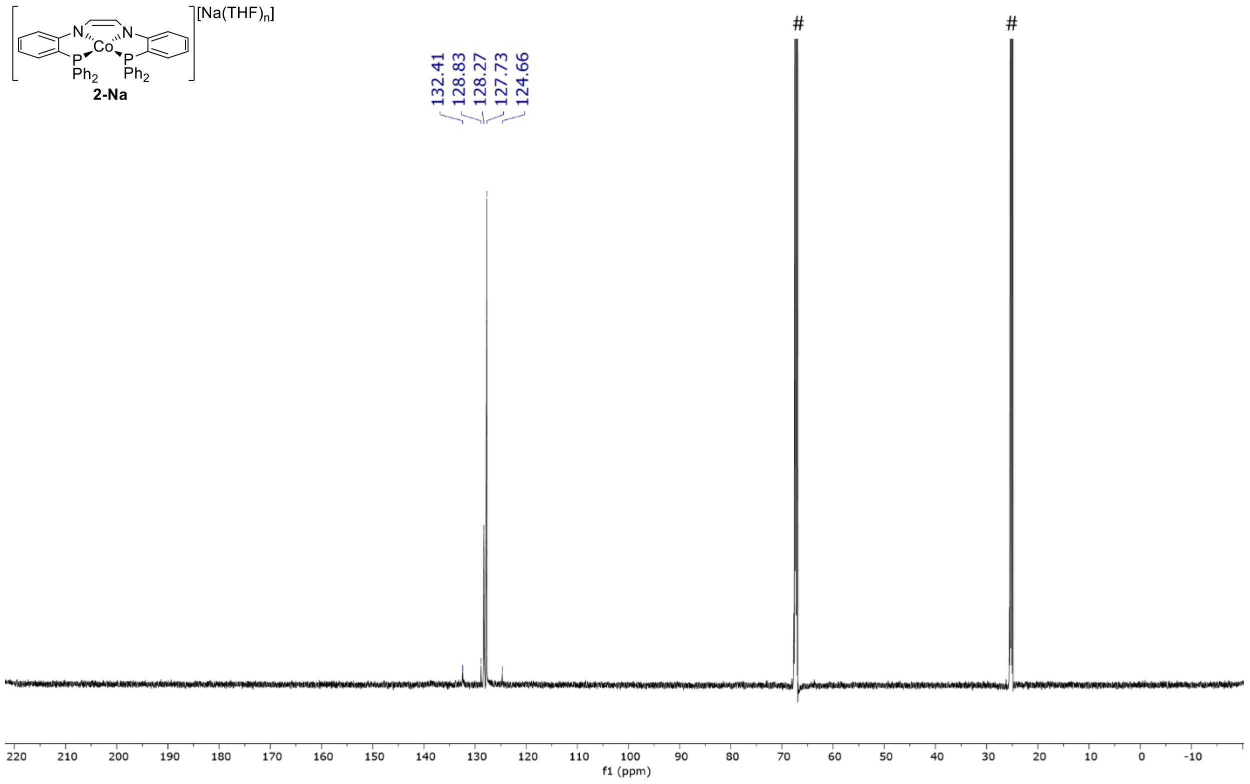

Figure S6. $^{31}P\{^{1}H\}$ NMR spectrum of **2-Na** (162 MHz, dioxane).

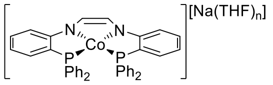

2-Na

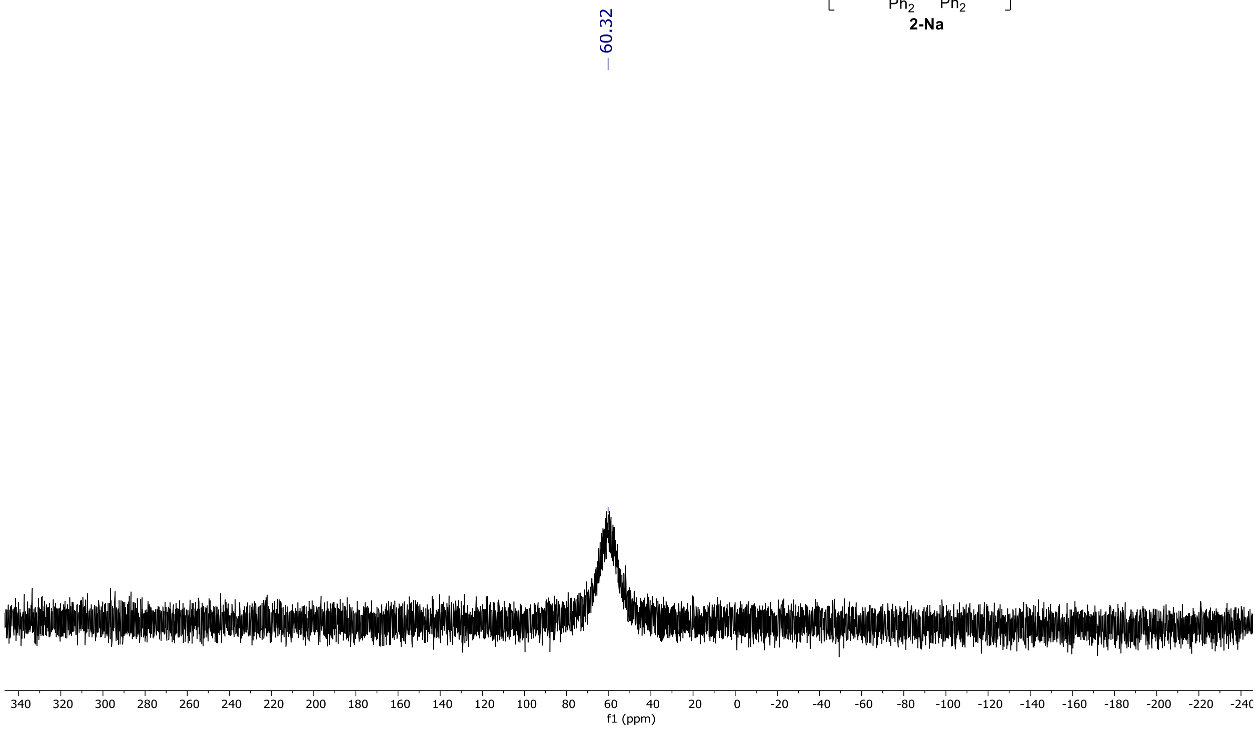

Figure S7. ^{1}H NMR spectrum of **2-Na** (600 MHz, THF-d_8) at temperatures ranging from 0 °C to -80 °C. THF is labeled with a #.

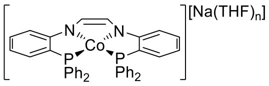

2-Na

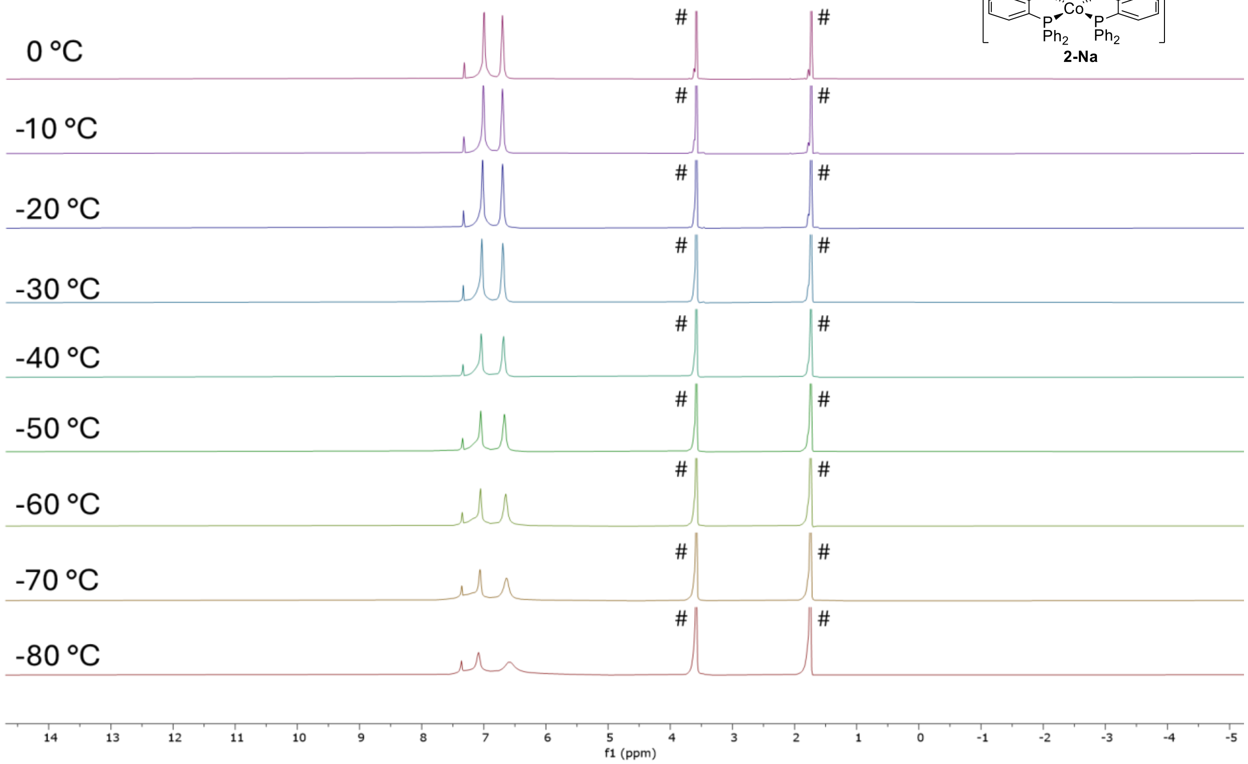

Discussion of the NMR spectra for 1-Na and 2-Na:

The $^{13}C\{^1H\}$ NMR spectra of **1-Na** and **2-Na** are not consistent with their proposed structures and are missing peaks that would be expected. Similarly, the ^1H NMR spectra for these complexes are broad and have a fewer number of peaks than expected for these complexes. We attempted to resolve the peaks in the ^1H NMR spectrum of **2-Na** through variable temperature NMR experiments (Figure S7) but were unsuccessful. We attribute the discrepancies in the NMR spectra of **1-Na** and **2-Na** to a fluctional process in solution that results in the overlap and/or broadening of resonances. The exact nature of this fluctional process is only speculative, but we believe it involves the interaction of the Na^+ counterion with the amide backbone of **1-Na** or **2-Na**. The crystal structure data supports this hypothesis with the Na^+ counterion in close proximity to the N atoms in the backbone of **1-Na** and **2-Na** (Figure 2, main text). Since K^+ derivatives of these complexes were synthesized in our previous report and the $^{31}P\{^1H\}$ NMR spectra of **1-Na** and **2-Na** are in agreement with the previously reported complexes, we did not investigate this fluxional process further. Additionally, we did not find any issues when using **1-Na** and **2-Na** to cleanly synthesize **1-R** and **2-R** derivatives via oxidative addition of alkyl halides, supporting the identity and purity of these molecules.

Figure S8. ^{1}H NMR spectrum of **1-CH$_3$** (700 MHz, C_6D_6). Residual C_6D_5H is denoted by #.

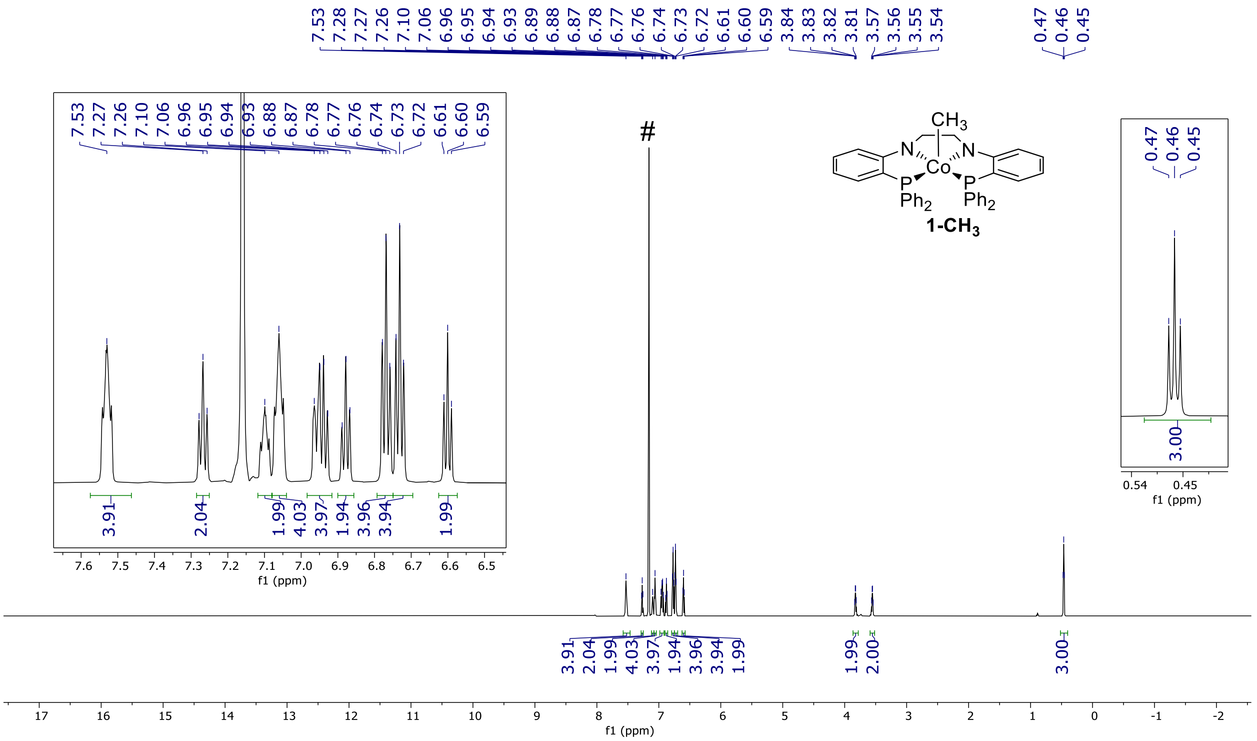

Figure S9. $^{13}C\{^1H\}$ NMR spectrum of **1-CH$_3$** (176 MHz, C_6D_6). C_6D_6 is denoted by #.

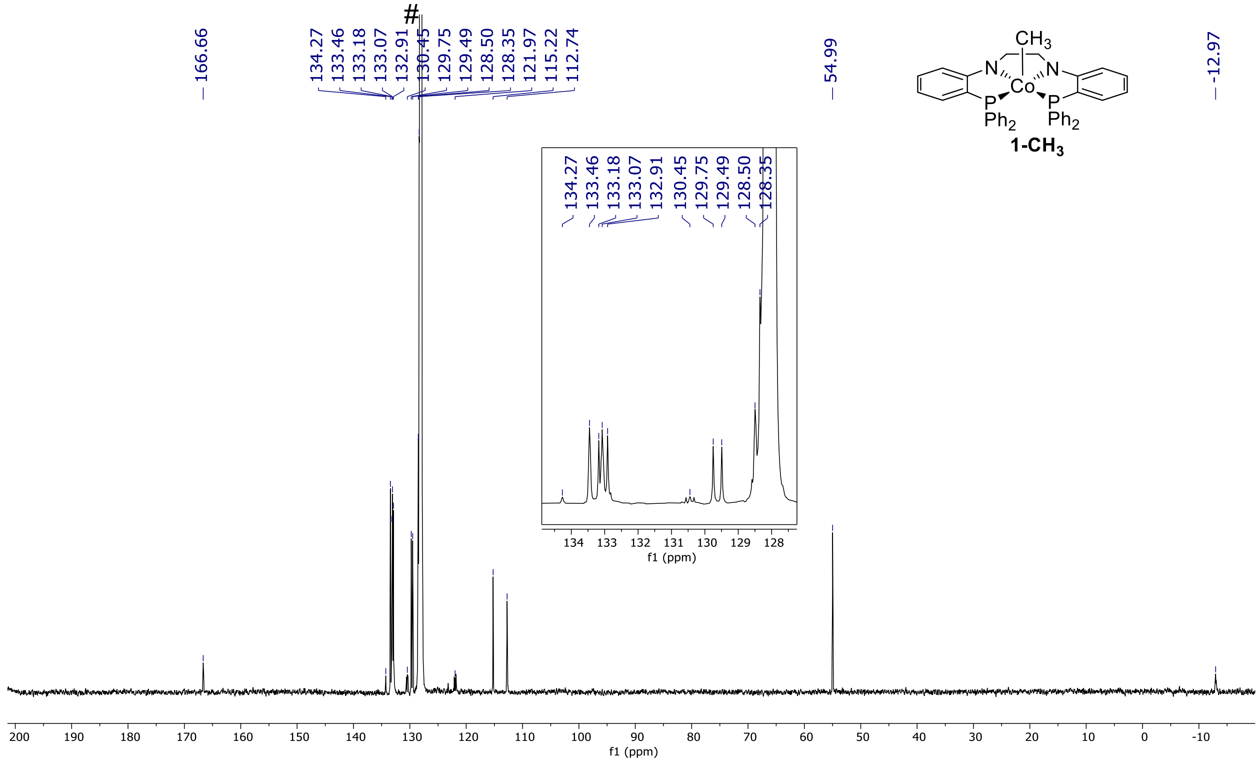

Figure S10. $^{31}P\{^1H\}$ NMR spectrum of **1-CH$_3$** (162 MHz in C_6D_6).

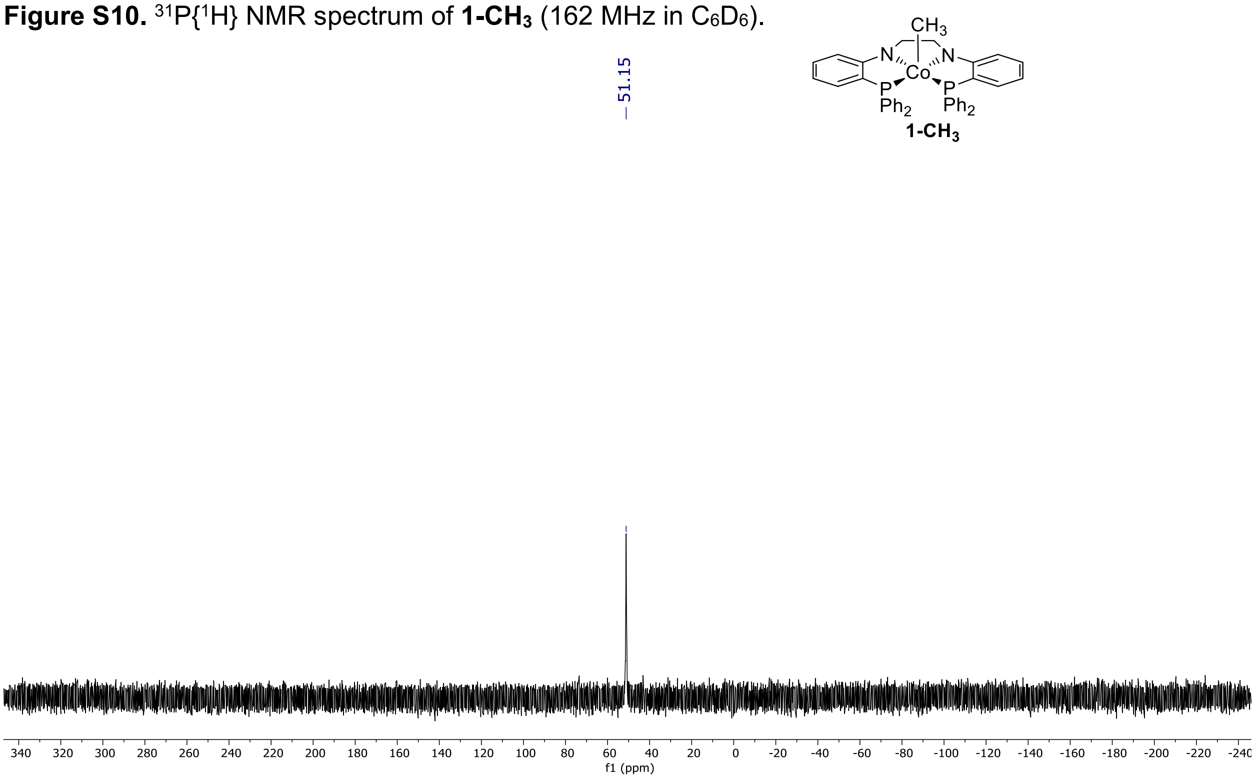

Figure S11. ^1H NMR spectrum of **1-Bu** (700 MHz, C_6D_6). Residual C_6D_5H is denoted by #.

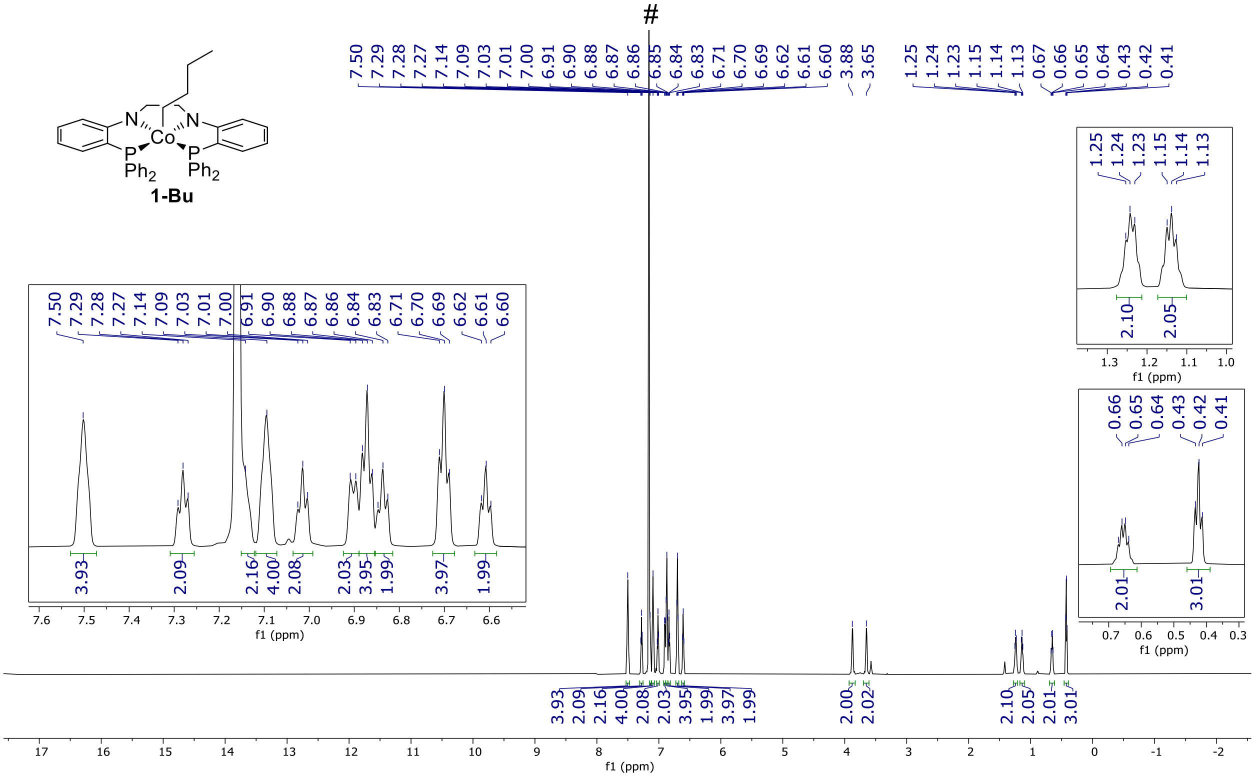

Figure S12. $^{31}P\{^{1}H\}$ NMR spectrum of **1-Bu** (162 MHz, C_6D_6).

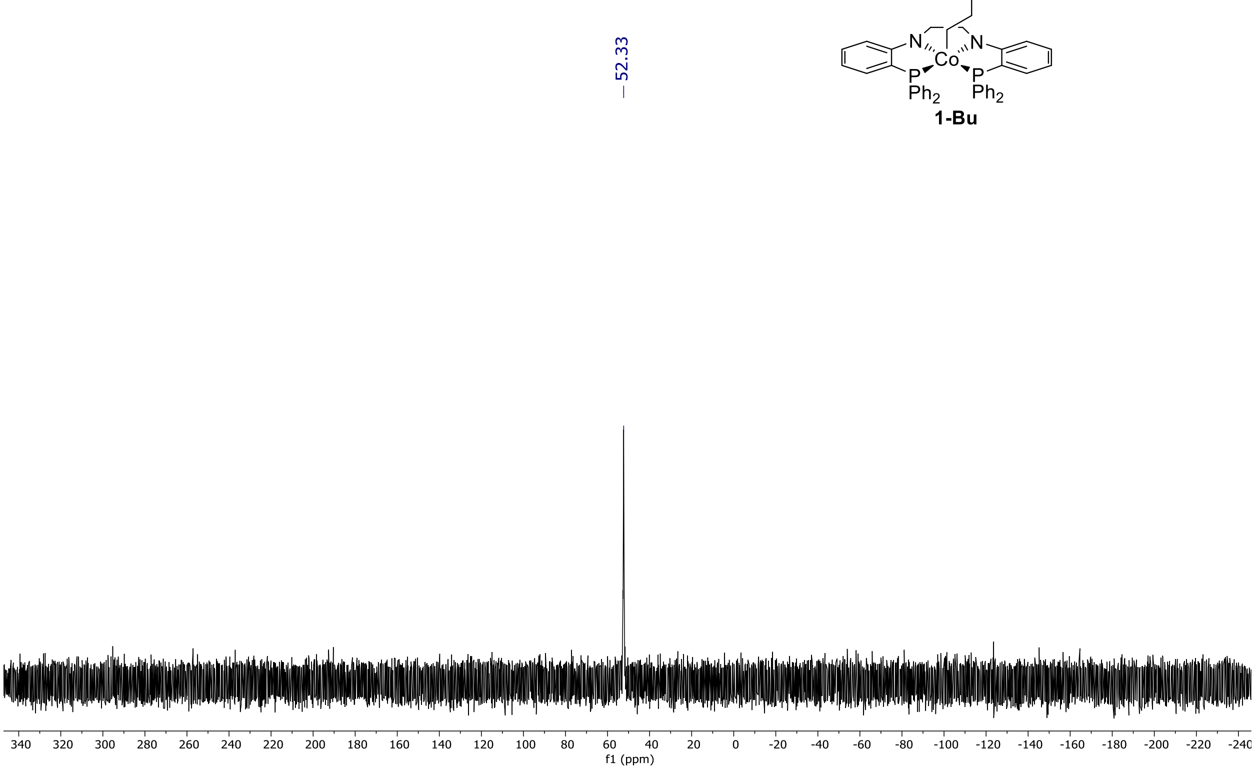

Figure S13. $^{13}C\{^{1}H\}$ NMR spectrum of **1-Bu** (176 MHz, C_6D_6). C_6D_6 is denoted by # and residual C_6H_6 is detonated with a *.

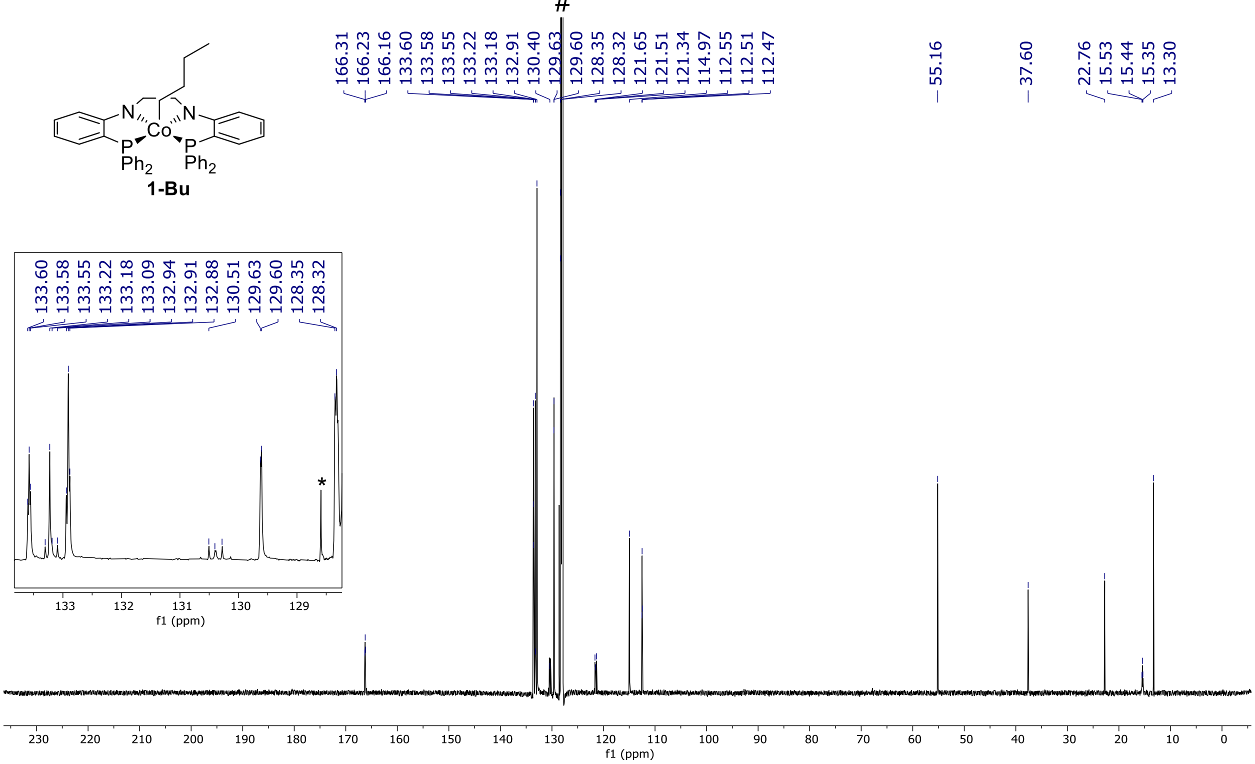

Figure S14. (Top) ^{13}C-^{1}H HSQC spectrum of **1-Bu** (700 MHz, C_6D_6). The cross peak assigned to CD_5H is labeled with a #. (Bottom) Showing only the aromatic region. The cross peak assigned to CD_5H is labeled with a #.

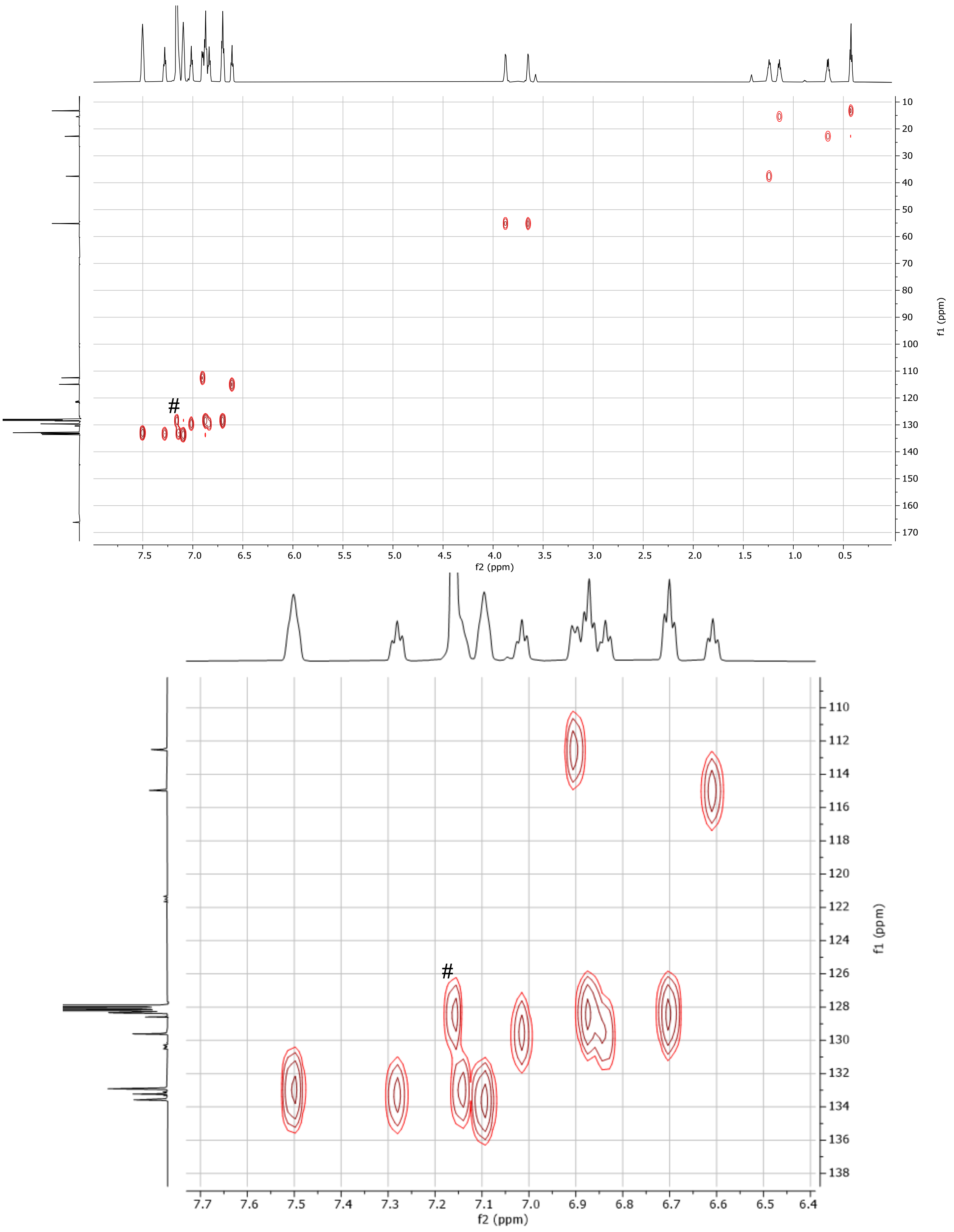

Figure S15. ^{1}H NMR spectrum of **1-Bn** (400 MHz, C_6D_6). Residual C_6D_5H is denoted by #, $(C_6H_5)CH_2Cl$ by a *, and bibenzyl by a "!".

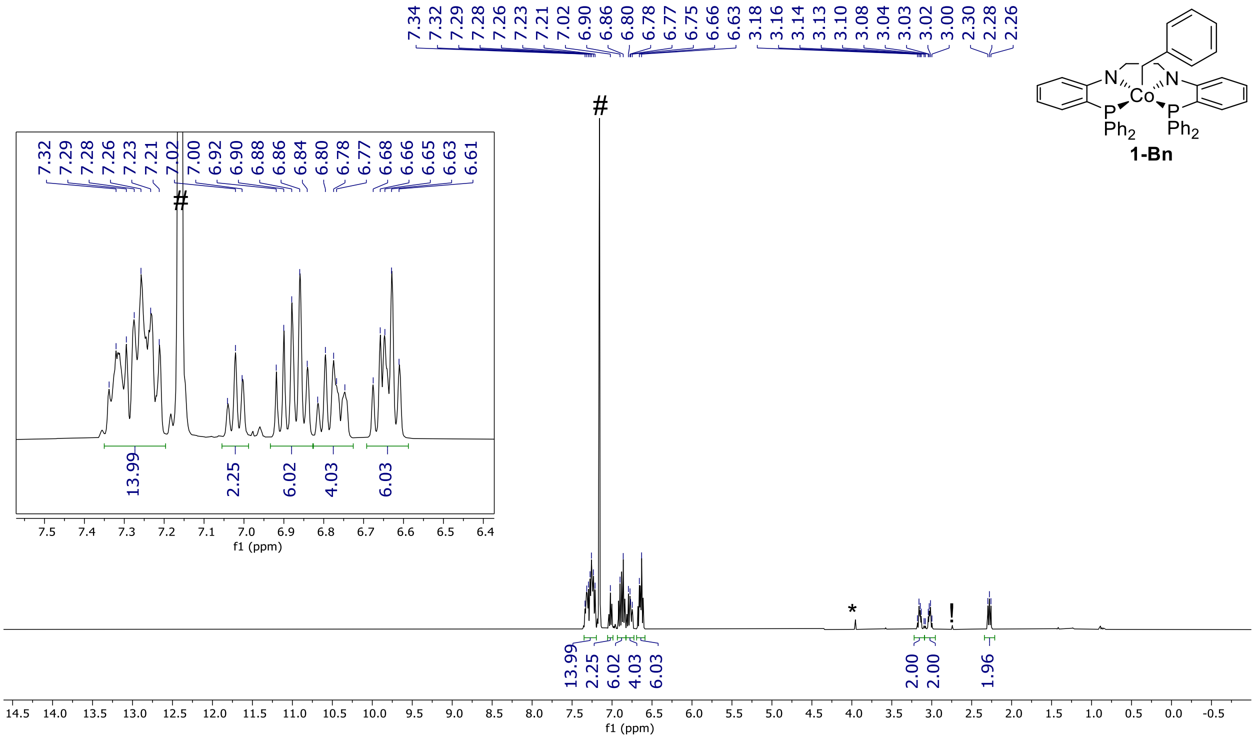

Figure S16. $^{31}P\{^{1}H\}$ NMR spectrum of **1-Bn** (162 MHz, C_6D_6).

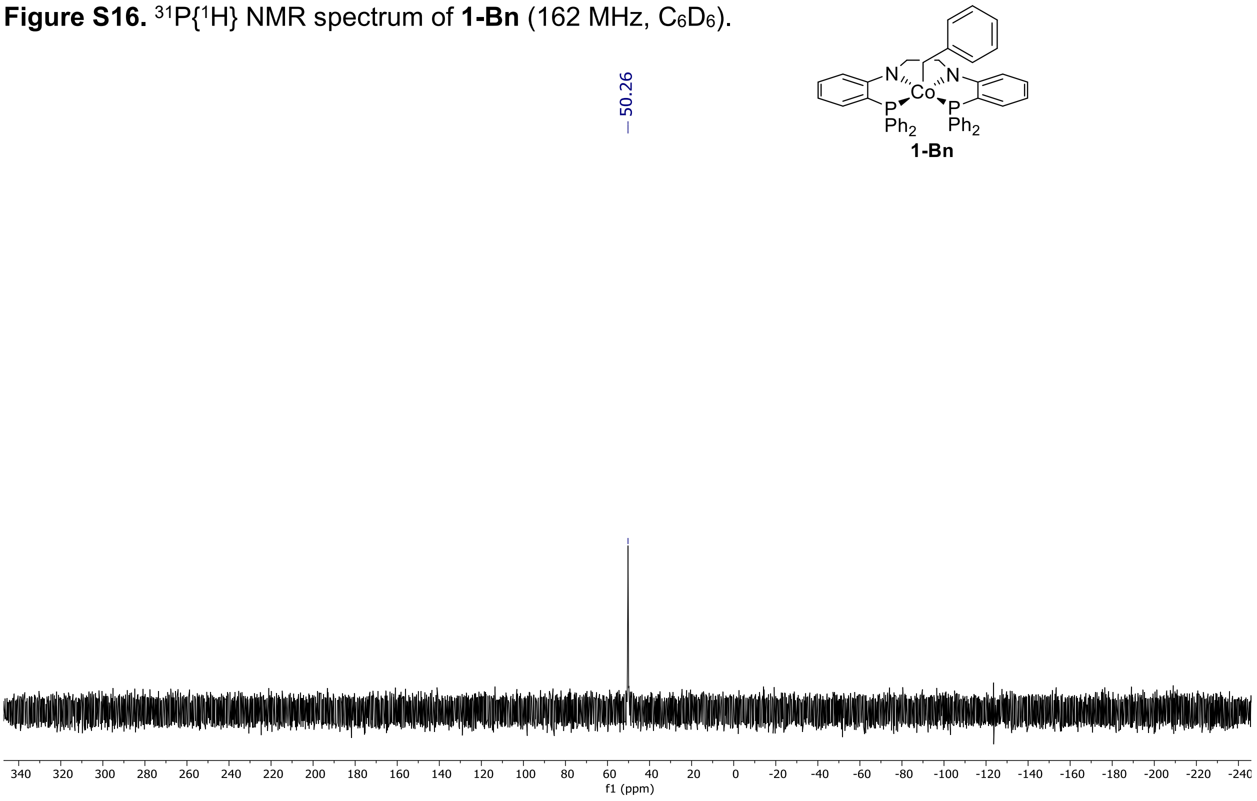

Figure S17. $^{13}C\{^1H\}$ NMR spectrum of **1-Bn** (176 MHz, C_6D_6). C_6D_6 is denoted by # and residual C_6H_6 is detonated with a *.

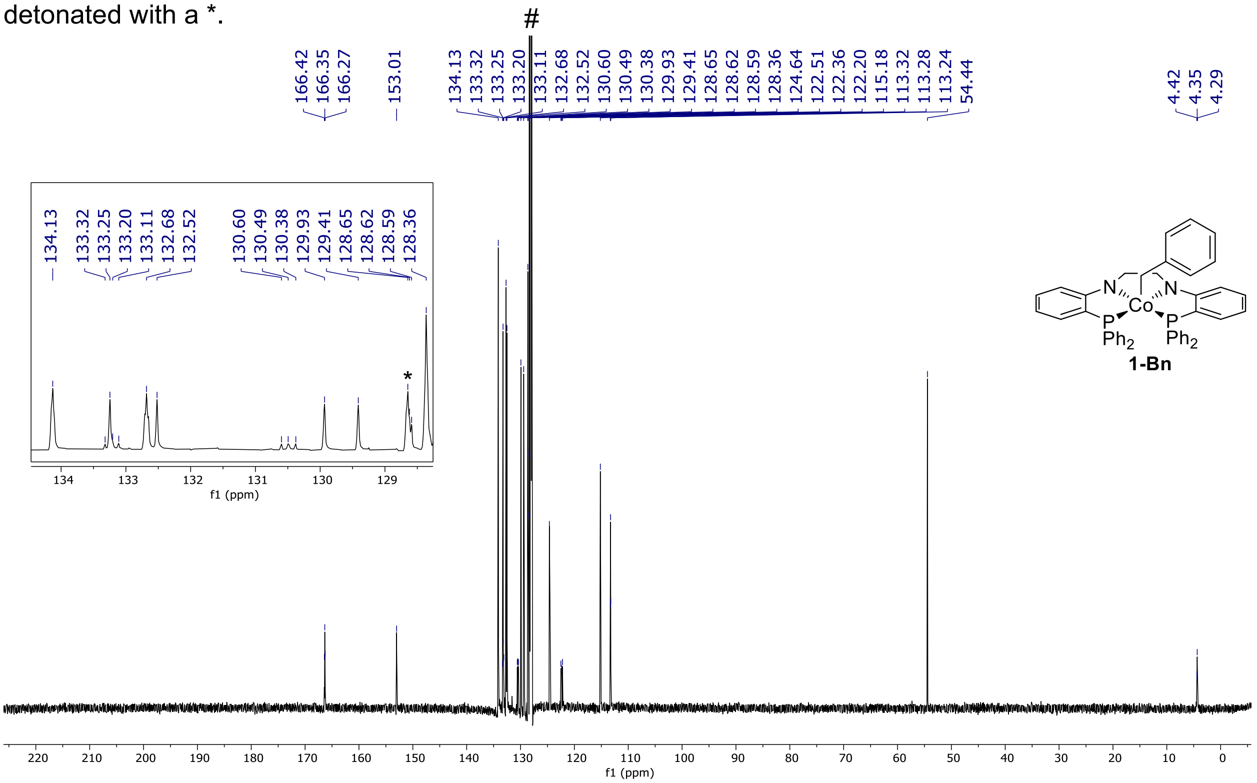

Figure S18. (Top) ^{13}C-^{1}H HSQC spectrum of **1-Bn** (700 MHz, C_6D_6). (Bottom) Showing the region with the cross peaks for the missing $^{13}C\{^{1}H\}$ and ^{1}H NMR resonances.

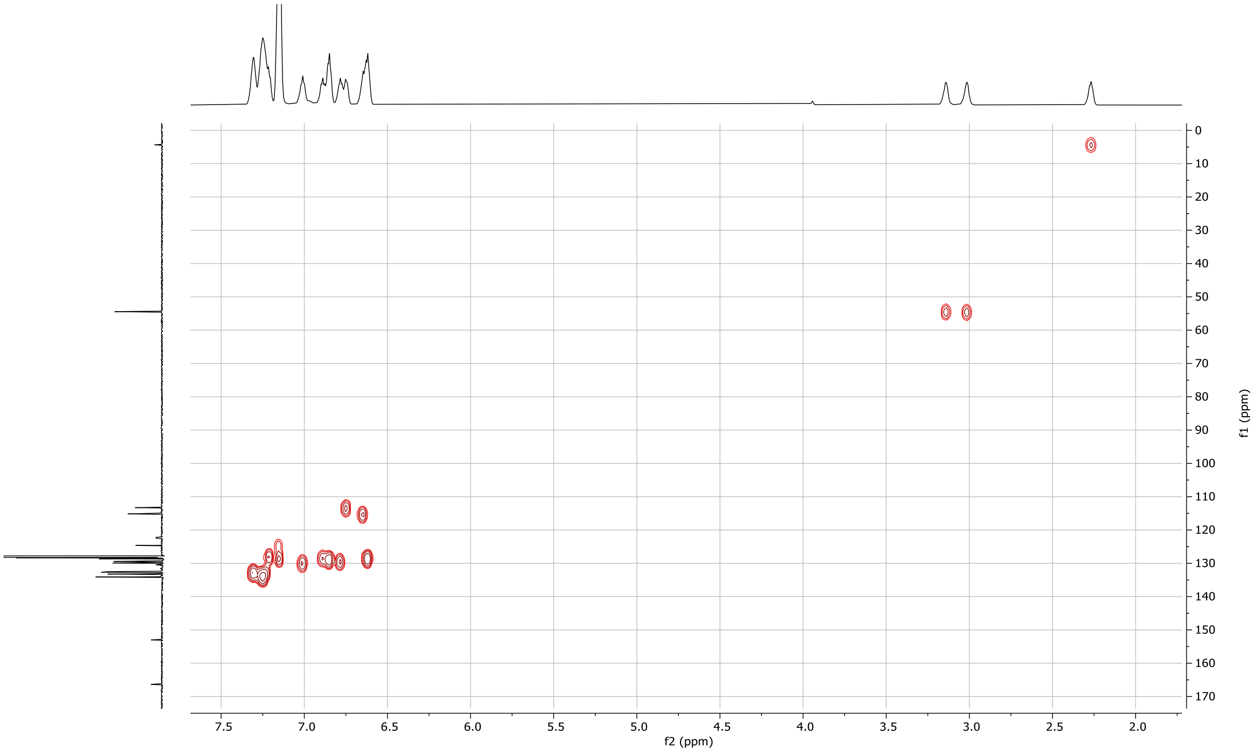

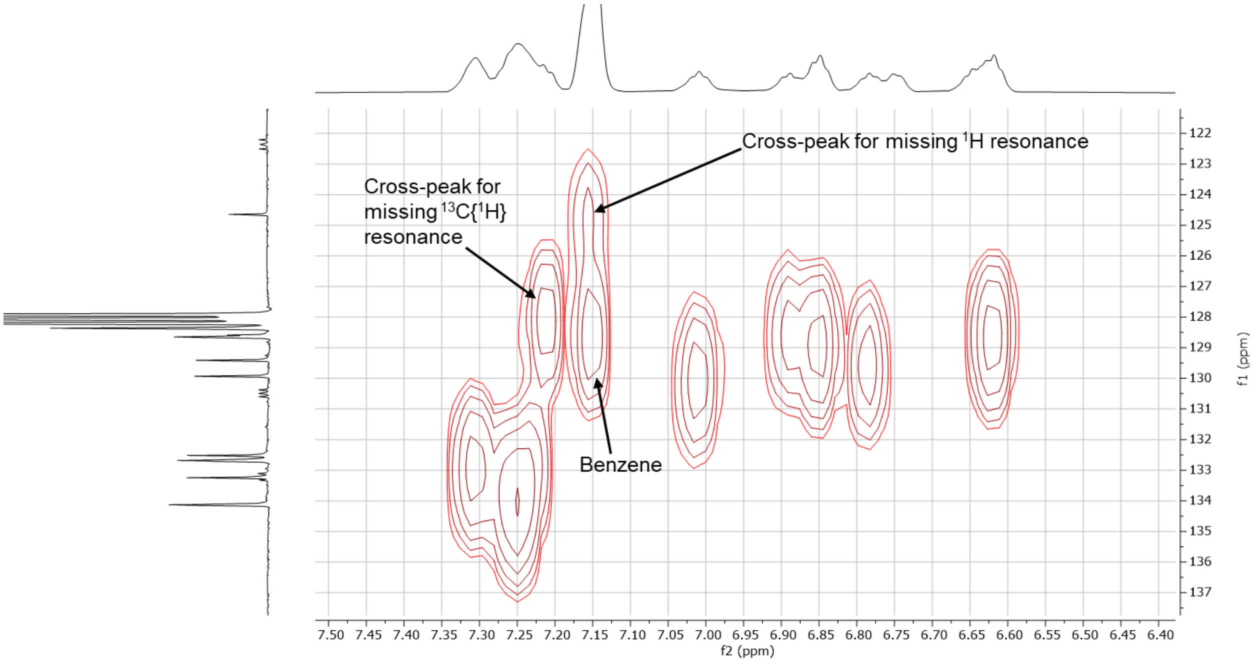

Figure S19. ^{1}H NMR spectrum of **2-CH$_3$** (700 MHz, C_6D_6). Residual C_6D_5H is denoted by a # and residual THF is denoted by a *.

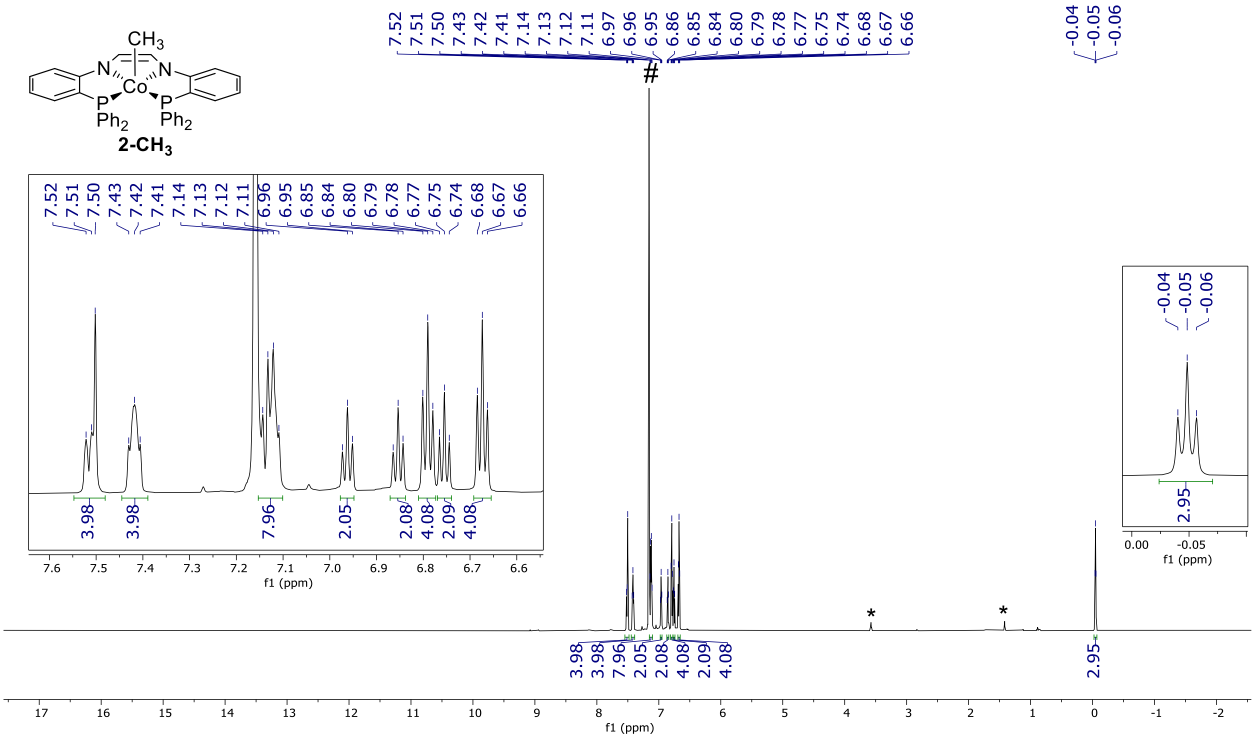

Figure S20. $^{31}P\{^{1}H\}$ NMR spectrum of **2-CH$_3$** (162 MHz, C_6D_6).

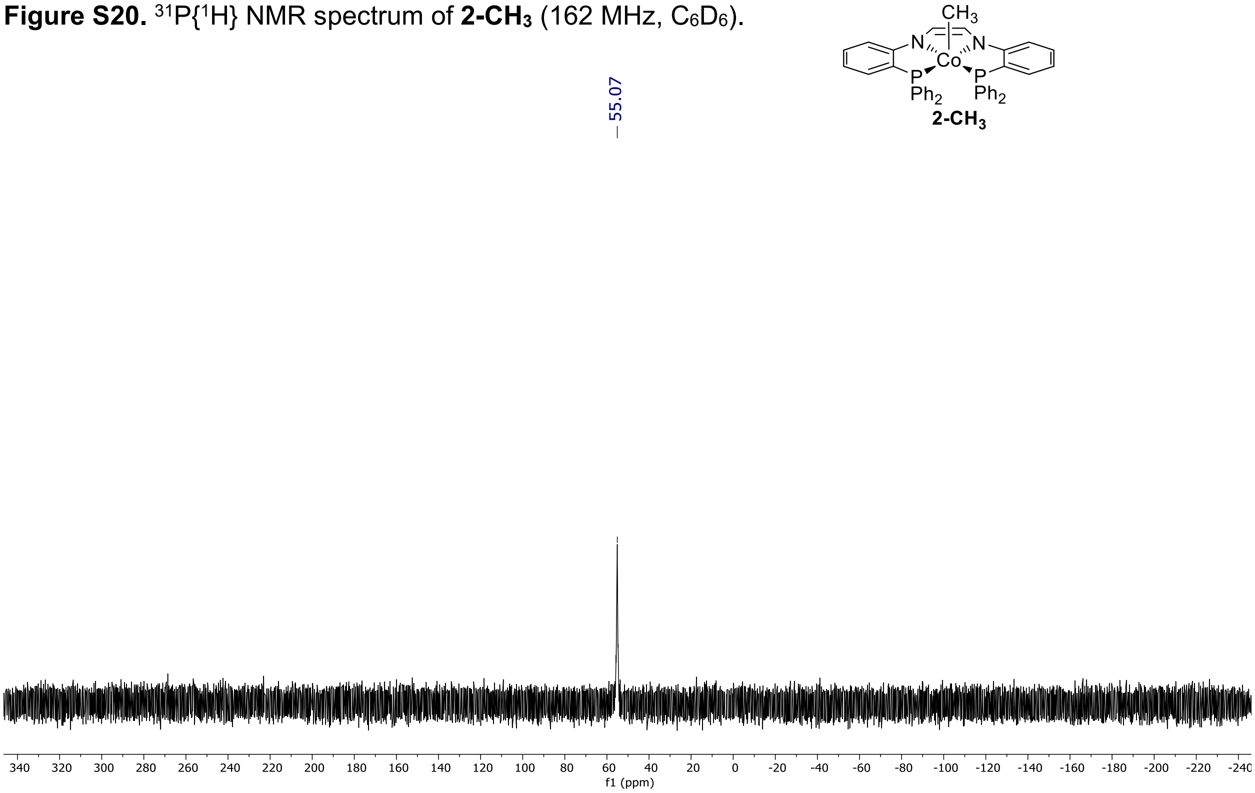

Figure S21. $^{13}C\{^{1}H\}$ NMR spectrum of **2-CH₃** (176 MHz, C_6D_6). C_6D_6 is denoted by # and residual C_6H_6 is detonated with a *.

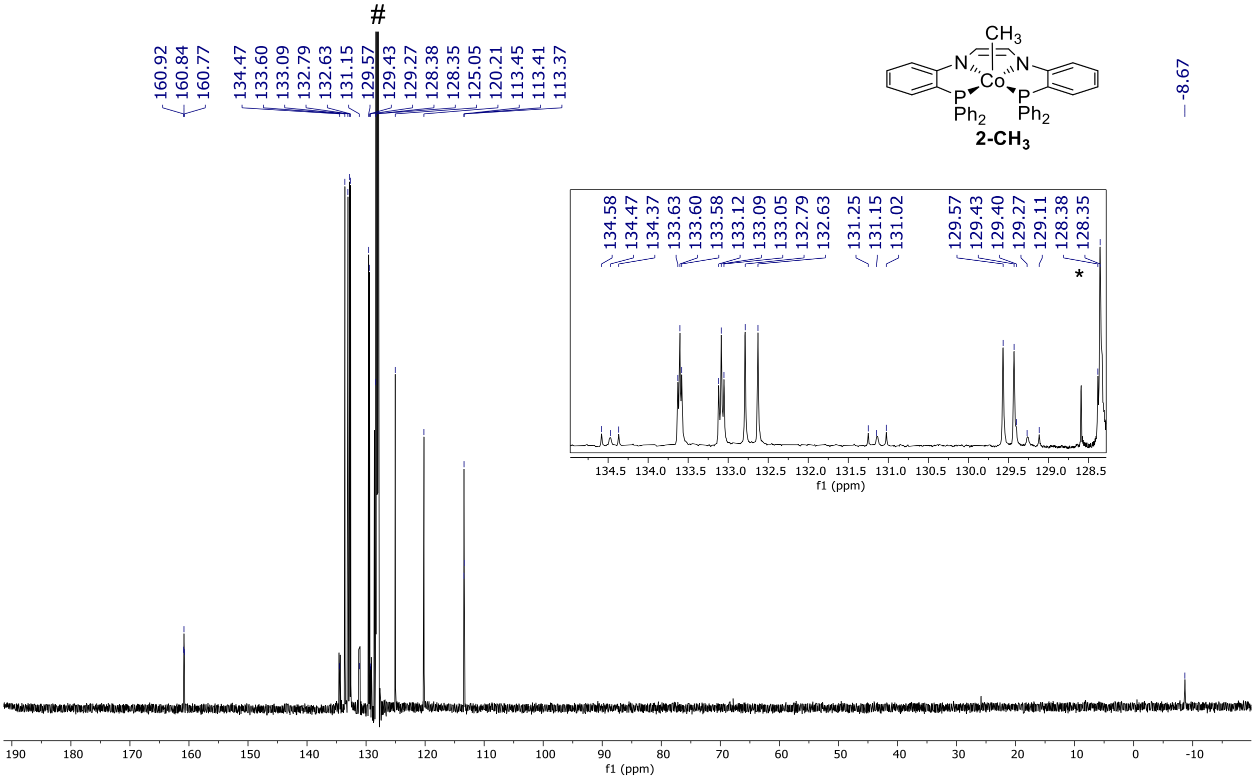

Figure S22. ^{1}H NMR spectrum of **2-Bu** (400 MHz, C_6D_6). Residual C_6D_5H is denoted by a # and residual THF is denoted by a *.

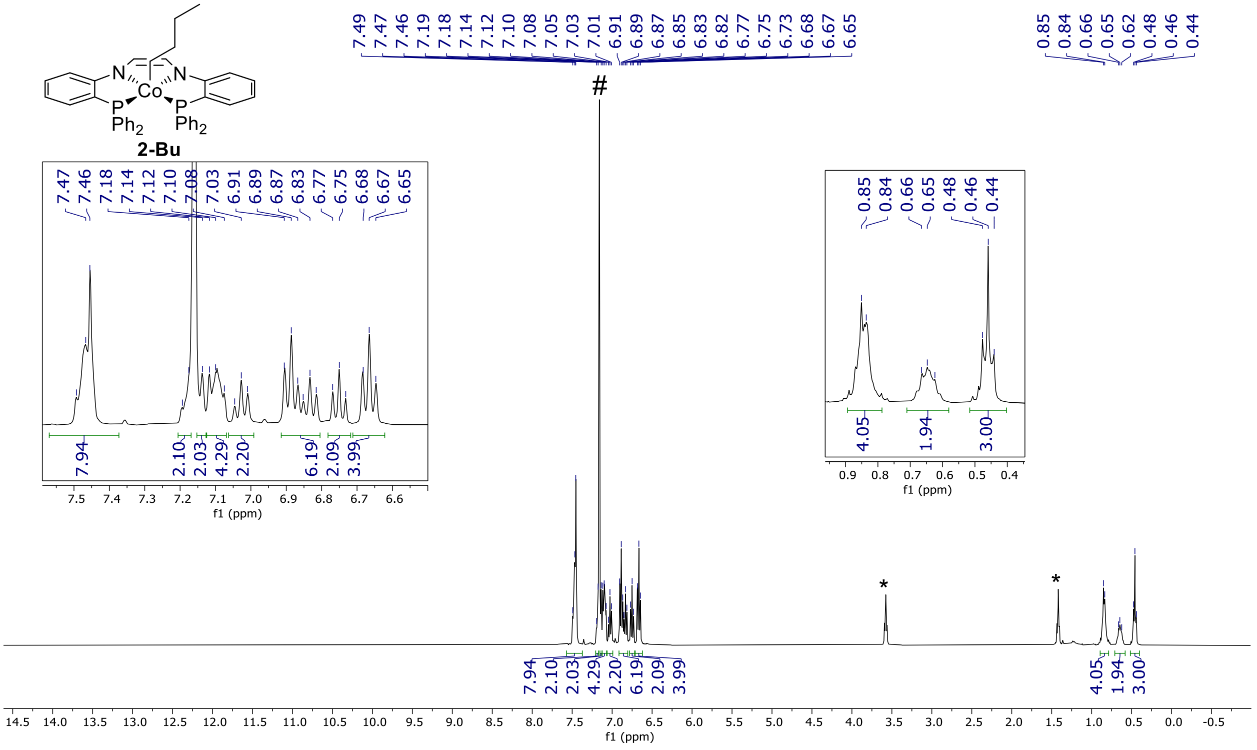

Figure S23. $^{31}P\{^1H\}$ NMR spectrum of **2-Bu** (162 MHz, C_6D_6).

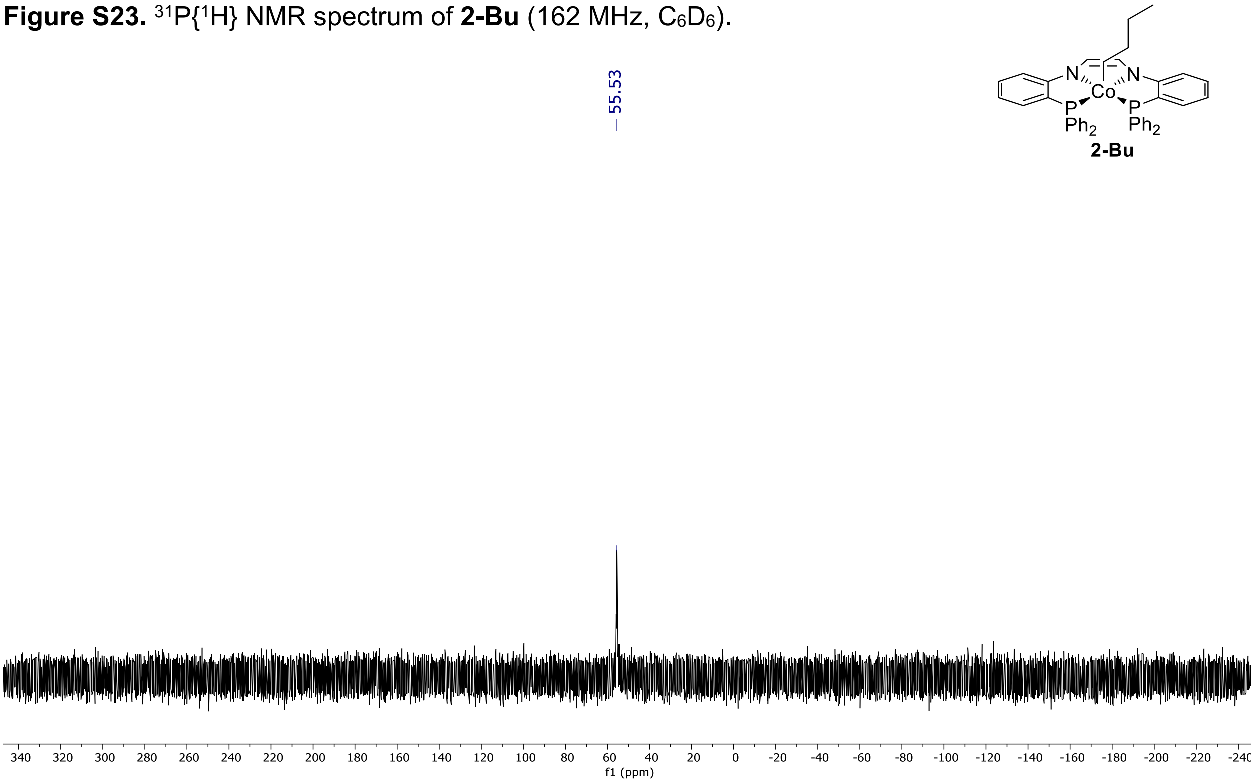

Figure S24. $^{13}C\{^1H\}$ NMR spectrum of **2-Bu** (176 MHz, C_6D_6). C_6D_6 is denoted by # and residual C_6H_6 is detonated with a *.

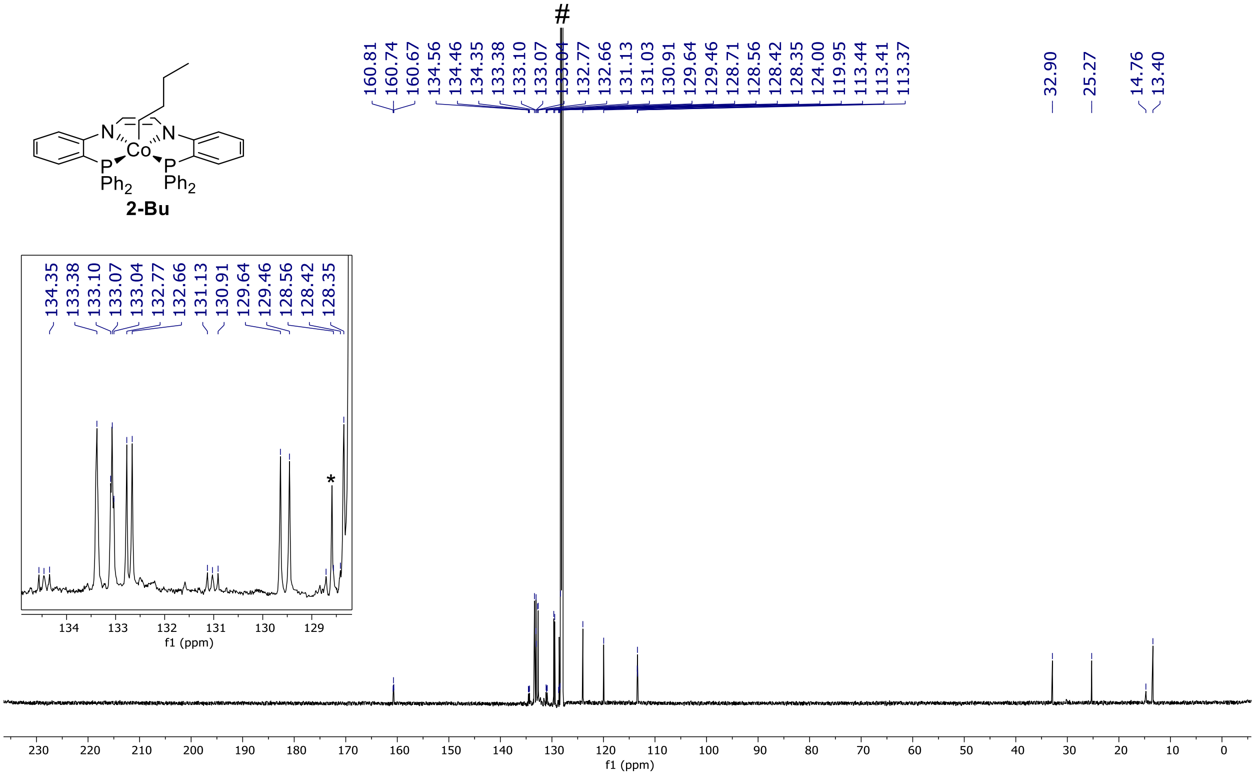

Figure S25. (Top) ^{13}C-^{1}H HSQC spectrum of **2-Bu** (700 MHz, C_6D_6). (Bottom) Showing the region with the cross peak for the missing $^{13}C\{^{1}H\}$ resonance.

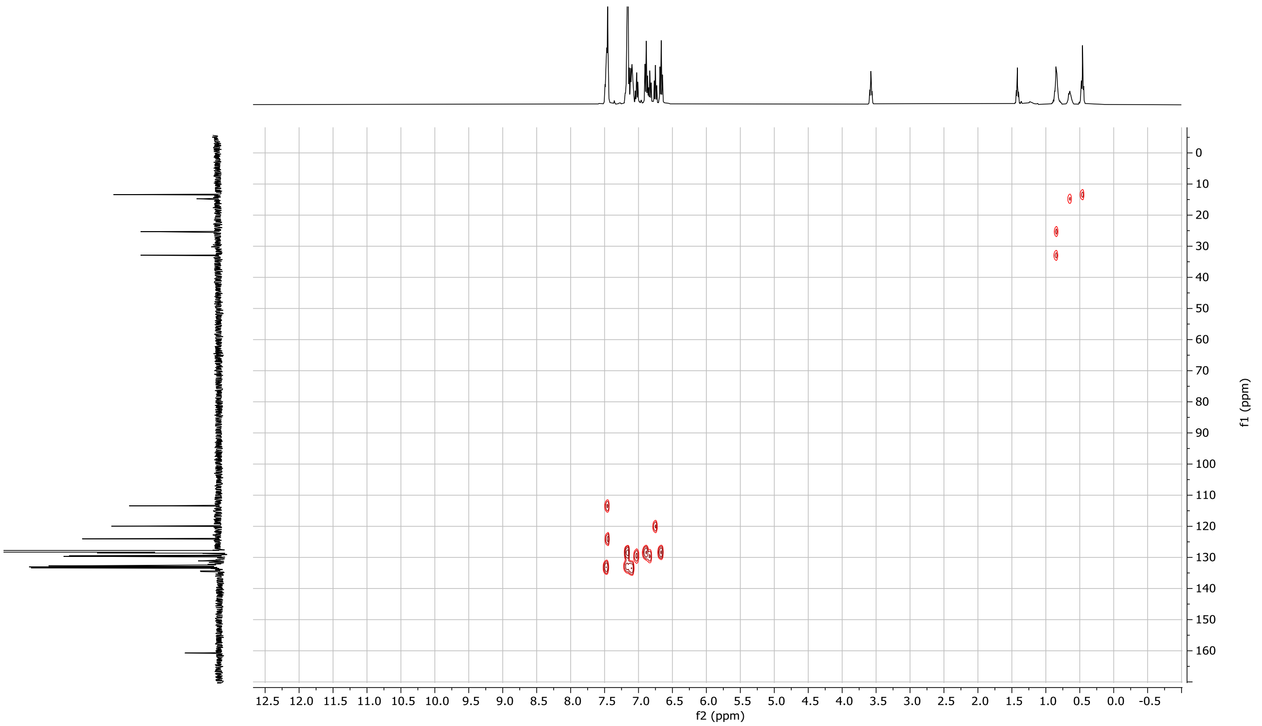

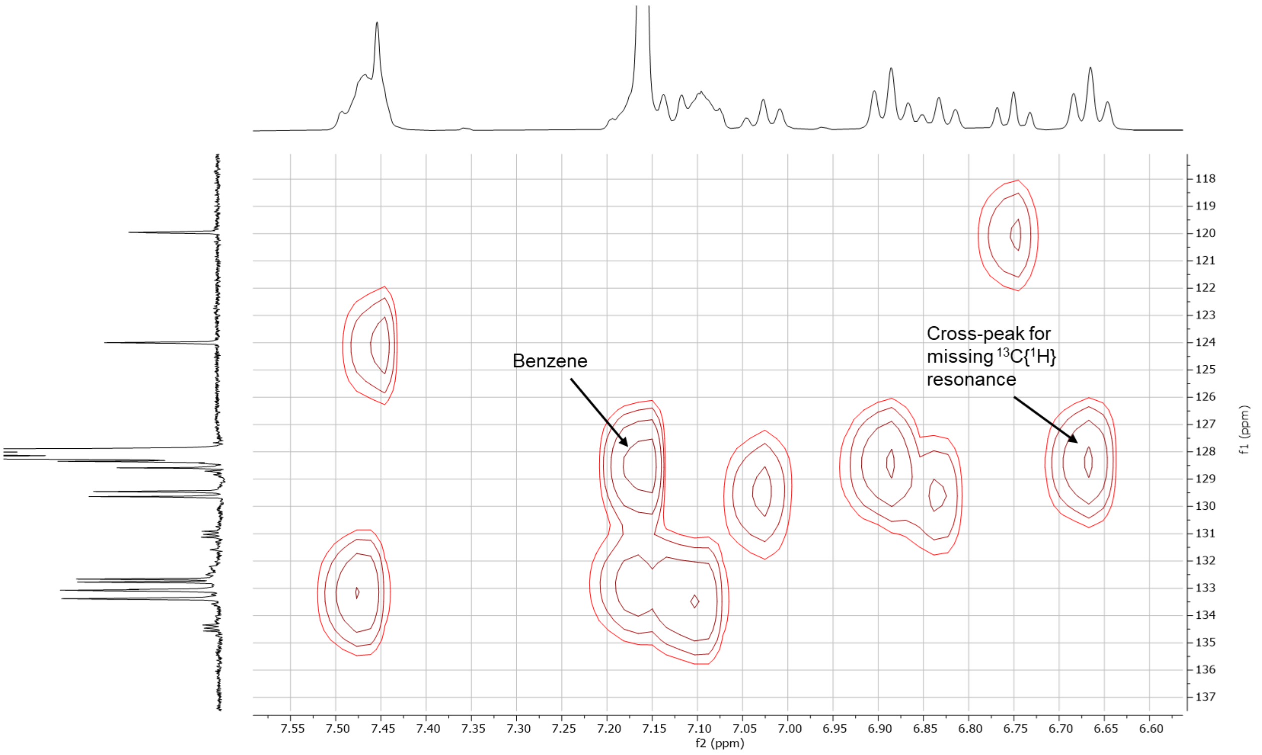

Figure S26. ^{1}H NMR spectrum of **2-Bn** (700 MHz, C_6D_6). Residual C_6D_5H is denoted by #.

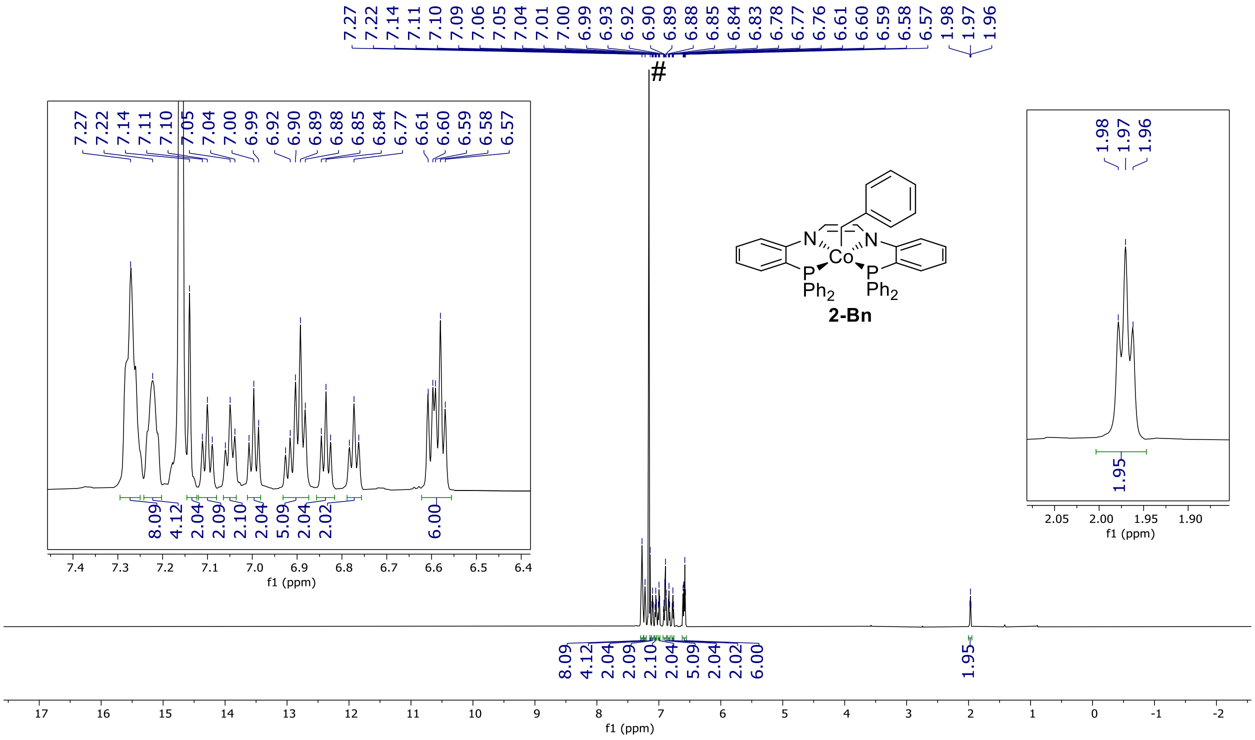

Figure S27. $^{31}P\{^{1}H\}$ NMR spectrum of **2-Bn** (162 MHz, C_6D_6).

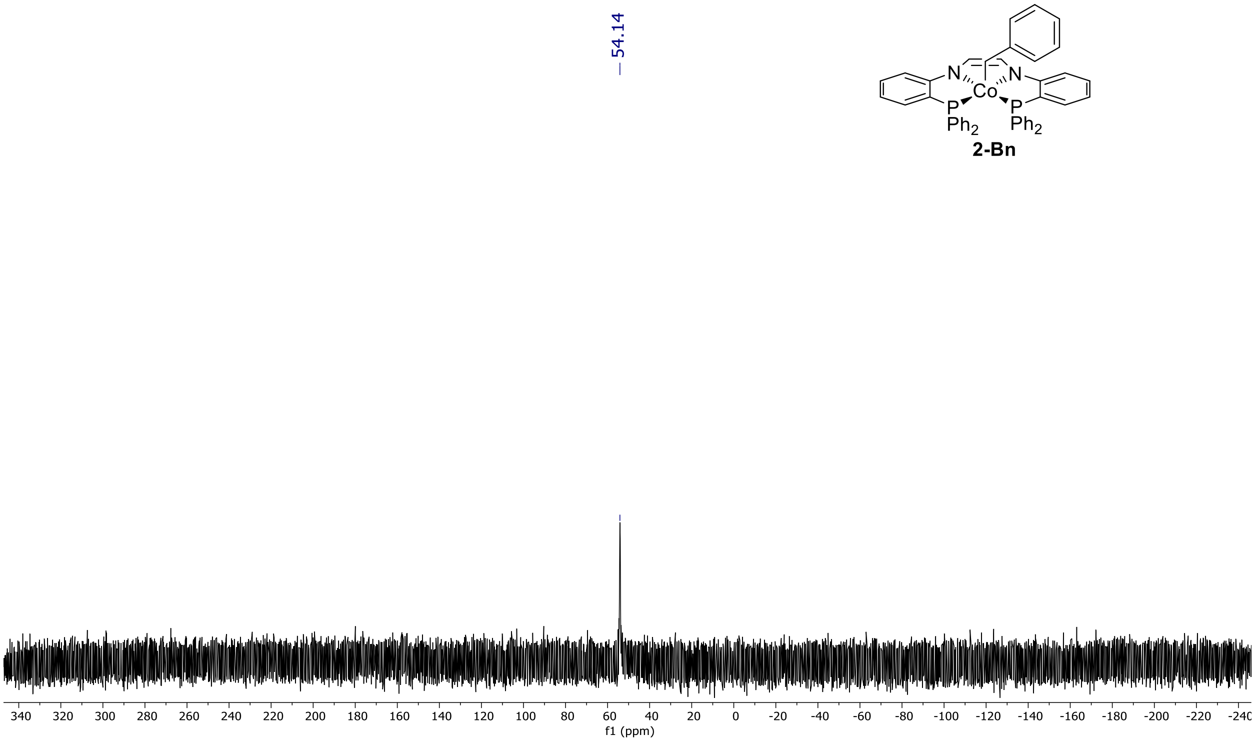

Figure S28. $^{13}C\{^1H\}$ NMR spectrum of **2-Bn** (176 MHz, C_6D_6). C_6D_6 is denoted by # and residual C_6H_6 is detonated with a *.

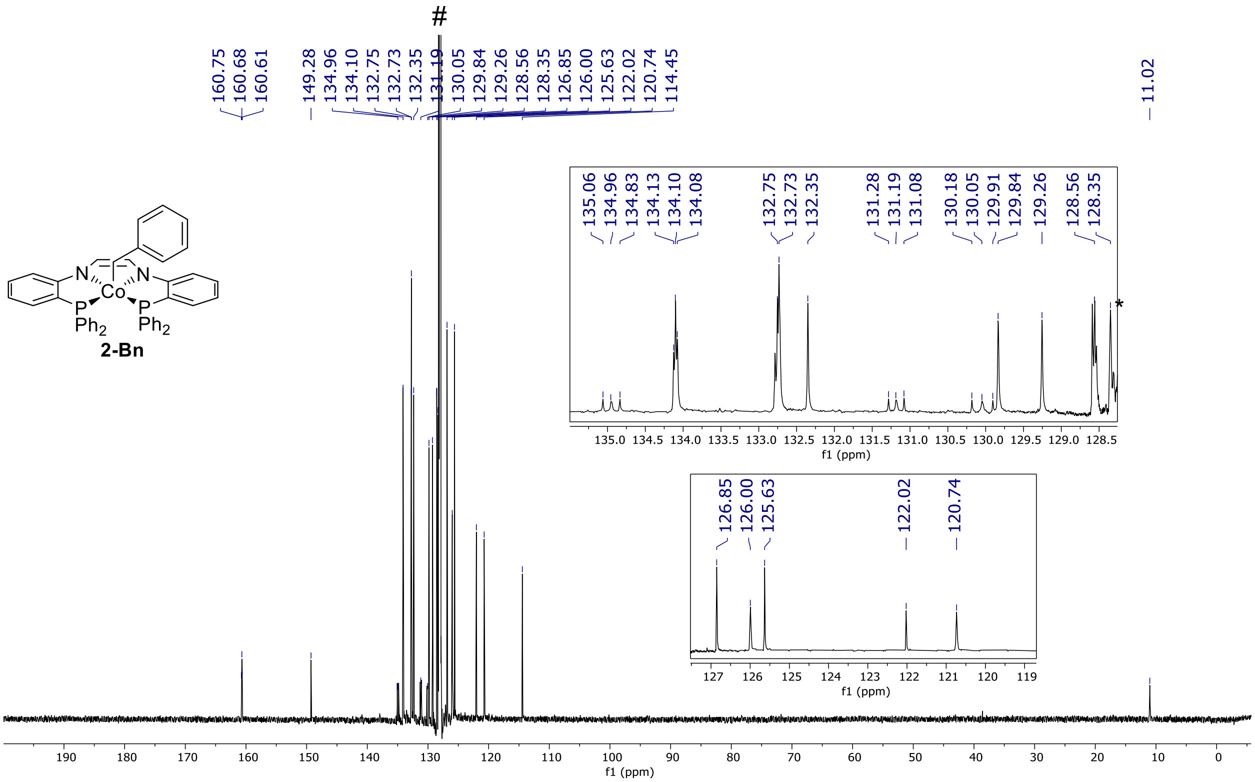

Thermolysis Experiments

Figure S29. ^{1}H NMR spectra (400 MHz, C_6D_6) of **1-CH$_3$** after thermal decomposition. NMR spectra taken before any heating (t_0) after heating for 15.7 h at 40 °C (t_1) then 5.5 h at 50 °C (t_2) then 1 h at 60 °C (t_3) then an additional 90 h at 60 °C (t_4). Residual C_6D_5H is denoted by # and residual solvents (Et_2O, THF, hexanes) are denoted with a *.

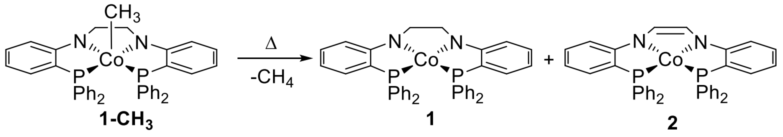

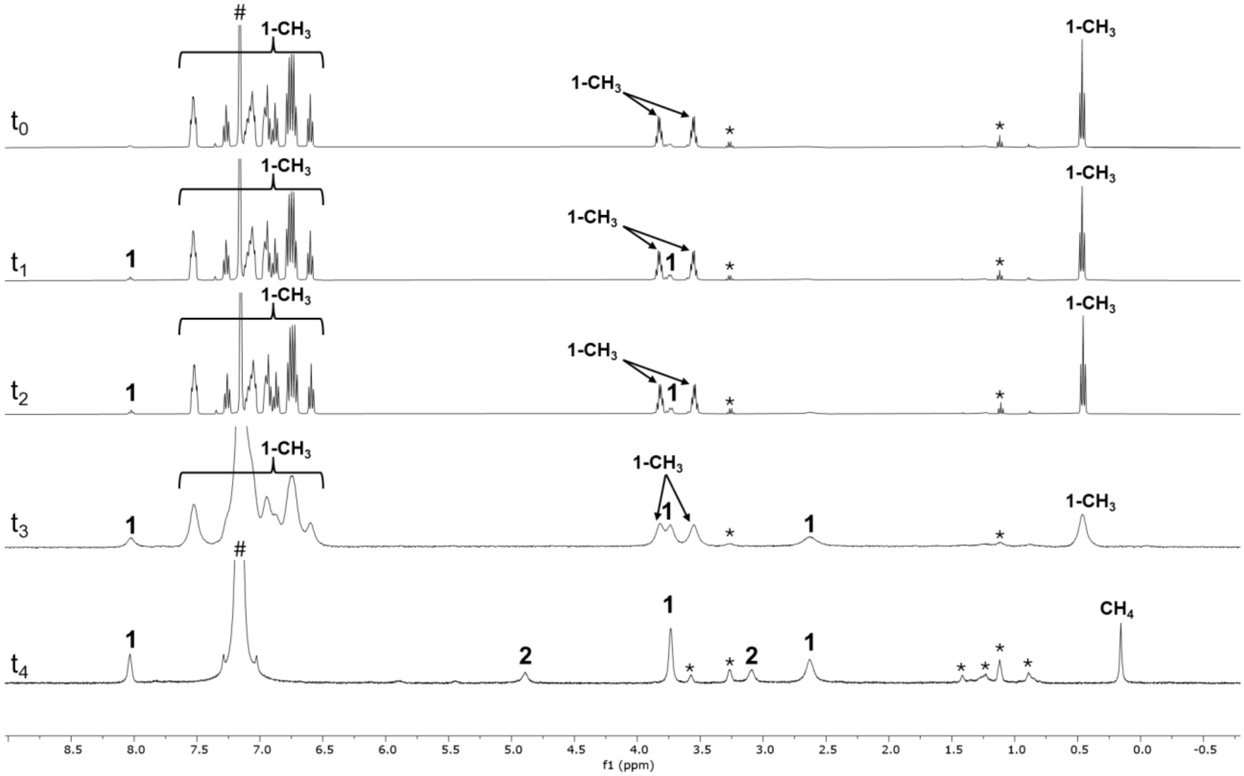

The ratio of **1** to **2** at the end of the experiment was determined to be 2:0.82 based on the relative integrations for the resonances at 2.63 ppm (8 H) for **1** and 3.08 ppm (8 H) for **2**. Due to the paramagnetic nature of **1** and **2** these values are only approximate and not quantitative.

Figure S30. ^{1}H NMR spectra (400 MHz, C_6D_6) of **1-Bu** after thermal decomposition at room temperature. NMR spectra taken after initial synthesis (t_0) after 2 days (t_1) after 3 days (t_2) and after 7 days (t_3). Residual C_6D_5H is denoted by #, butane is denoted by "B", and residual solvents (THF, hexanes) are denoted with a *.

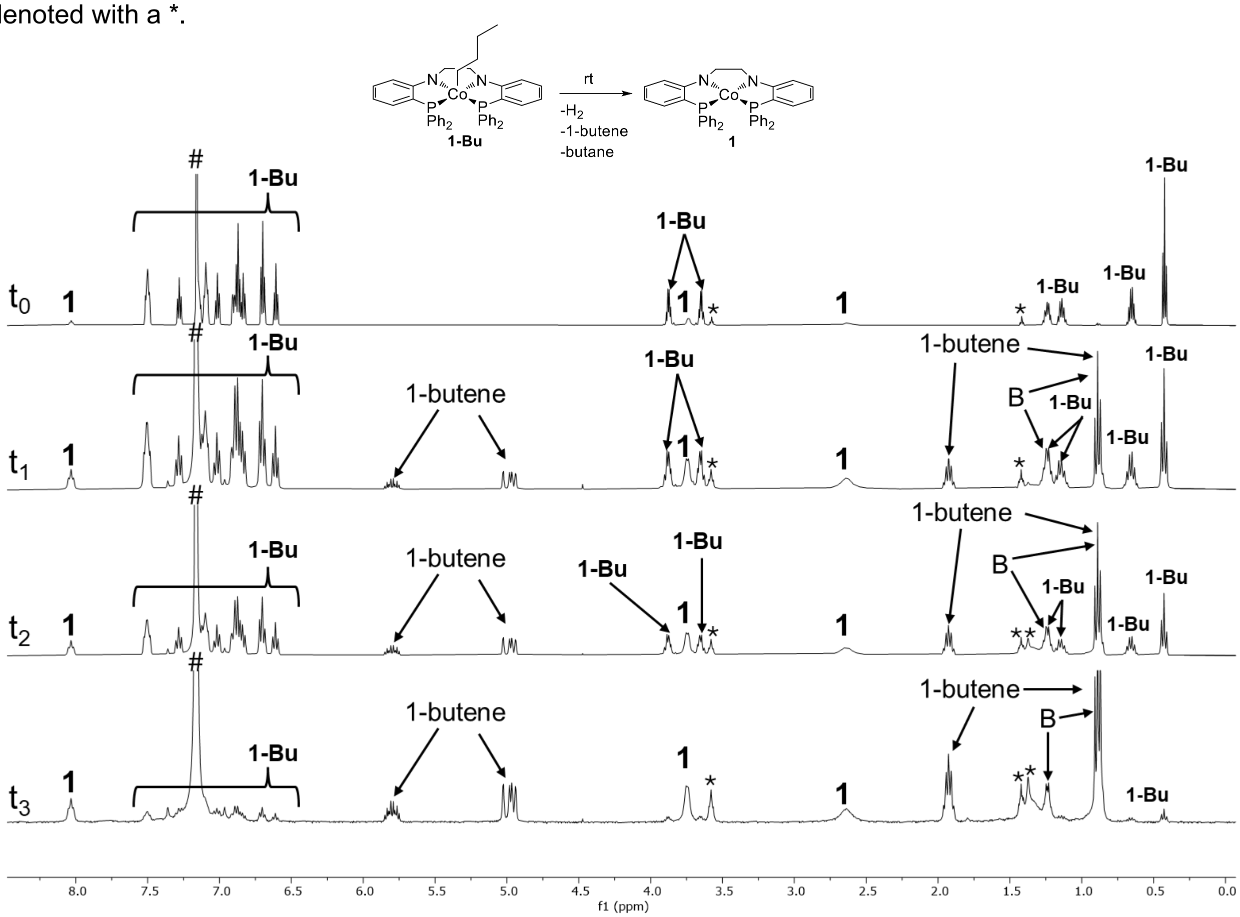

Figure S31. ^{1}H NMR spectra (600 MHz (top) and 400 MHz (bottom), C_6D_6) of **1-Bn** after thermal decomposition at room temperature. NMR spectra taken after initial synthesis (top) and after 8 days (bottom) Residual C_6D_5H is denoted by # and residual THF is denoted with a *.

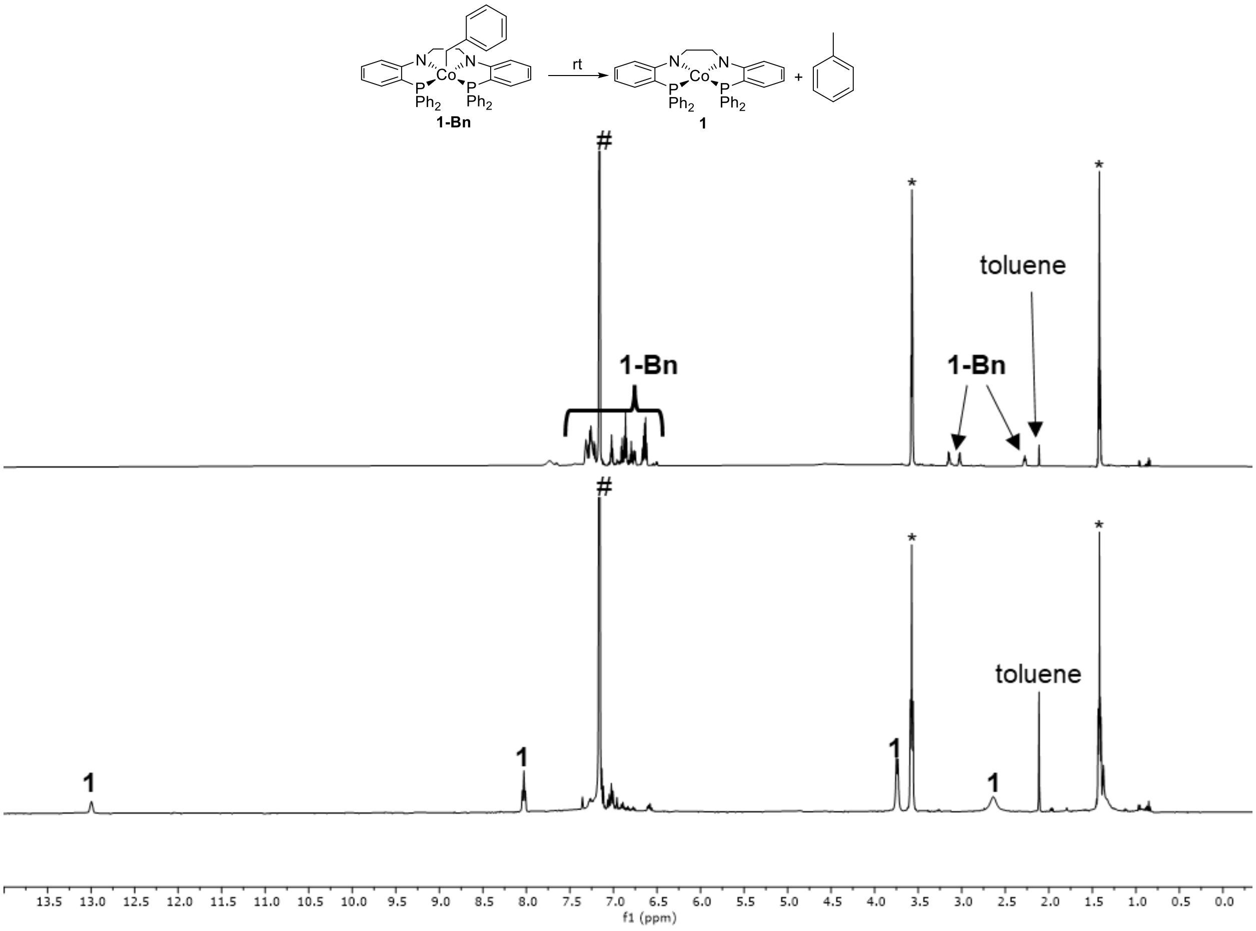

Figure S32. Final ^{1}H NMR spectrum (400 MHz, C_6D_6) of **2-CH$_3$** after attempted thermal decomposition at 60 °C for 2 days and 80 °C for 12 days. Residual C_6D_5H is denoted by # and residual solvents (THF, hexanes) are denoted by a *.

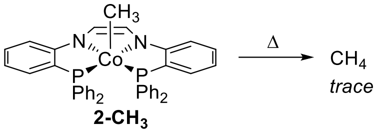

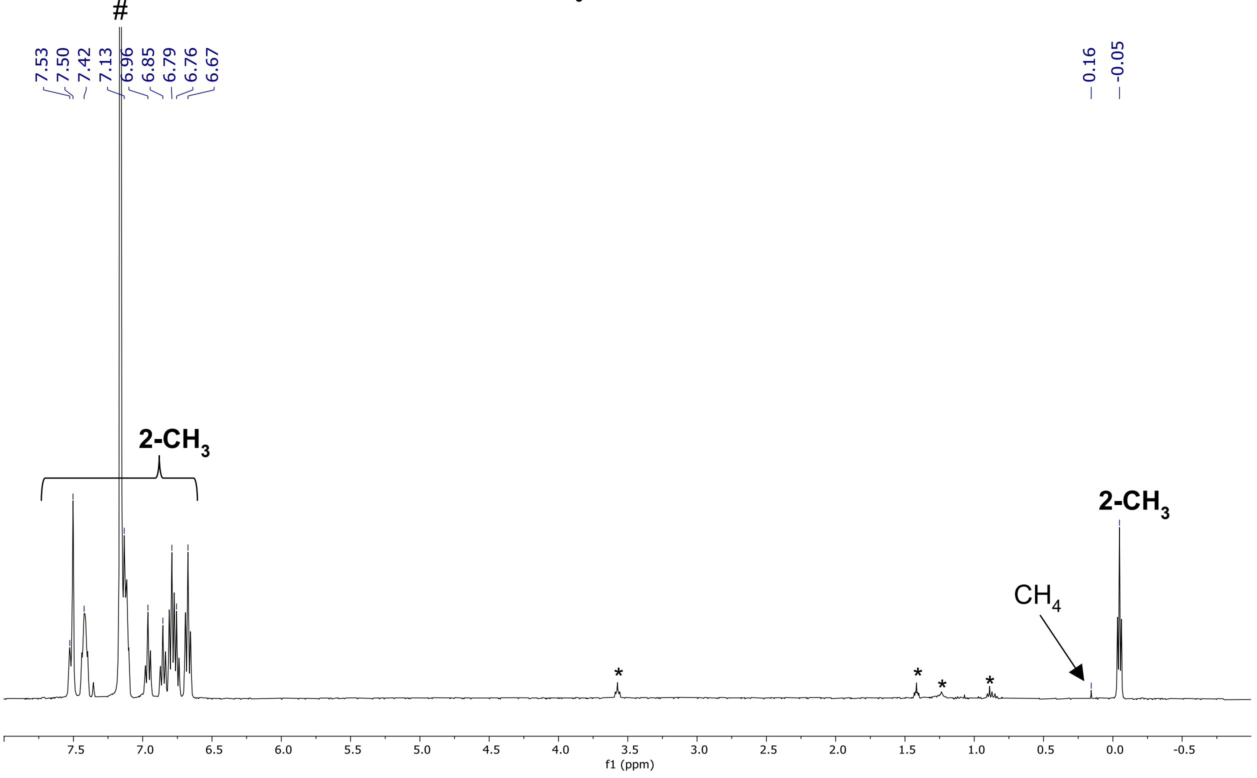

Figure S33. ^{1}H NMR spectra (400 MHz, C_6D_6) of **2-Bu** after thermal decomposition. NMR spectra were taken after initial synthesis (t_0) after 4 d at 45 °C (t_1) after 3 d at 60 °C (t_2) then after 1 d (t_3), 4 d (t_4) and 11 d (t_5) at 80 °C. Residual C_6D_5H is denoted by # and residual Et_2O is denoted with a *.

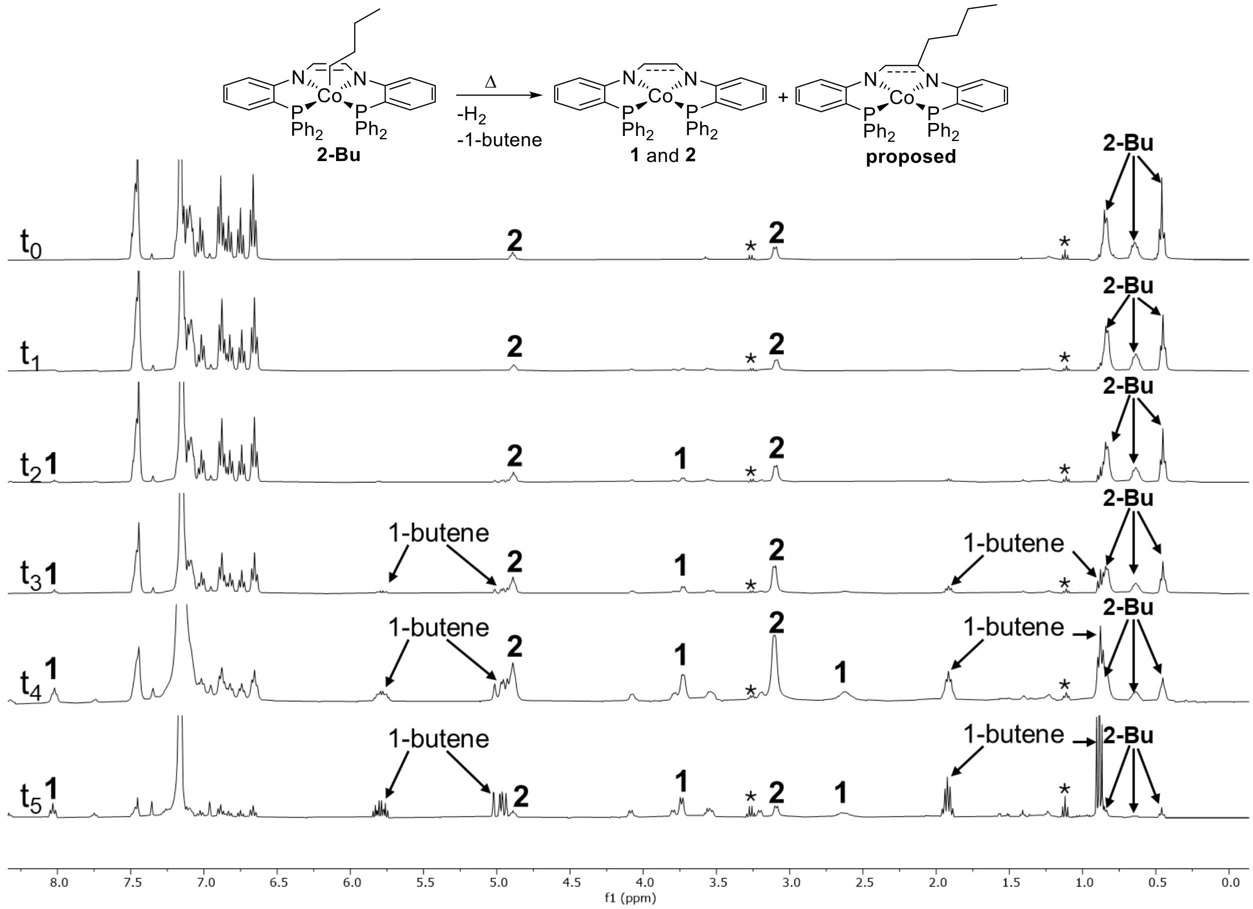

Figure S34. Final paramagnetic ^1H NMR spectra (400 MHz, C_6D_6) of products remaining after thermal decomposition of **2-Bu**. Residual C_6D_5H is denoted by # and residual Et_2O is denoted with a *. The proposed product is labeled with a "?".

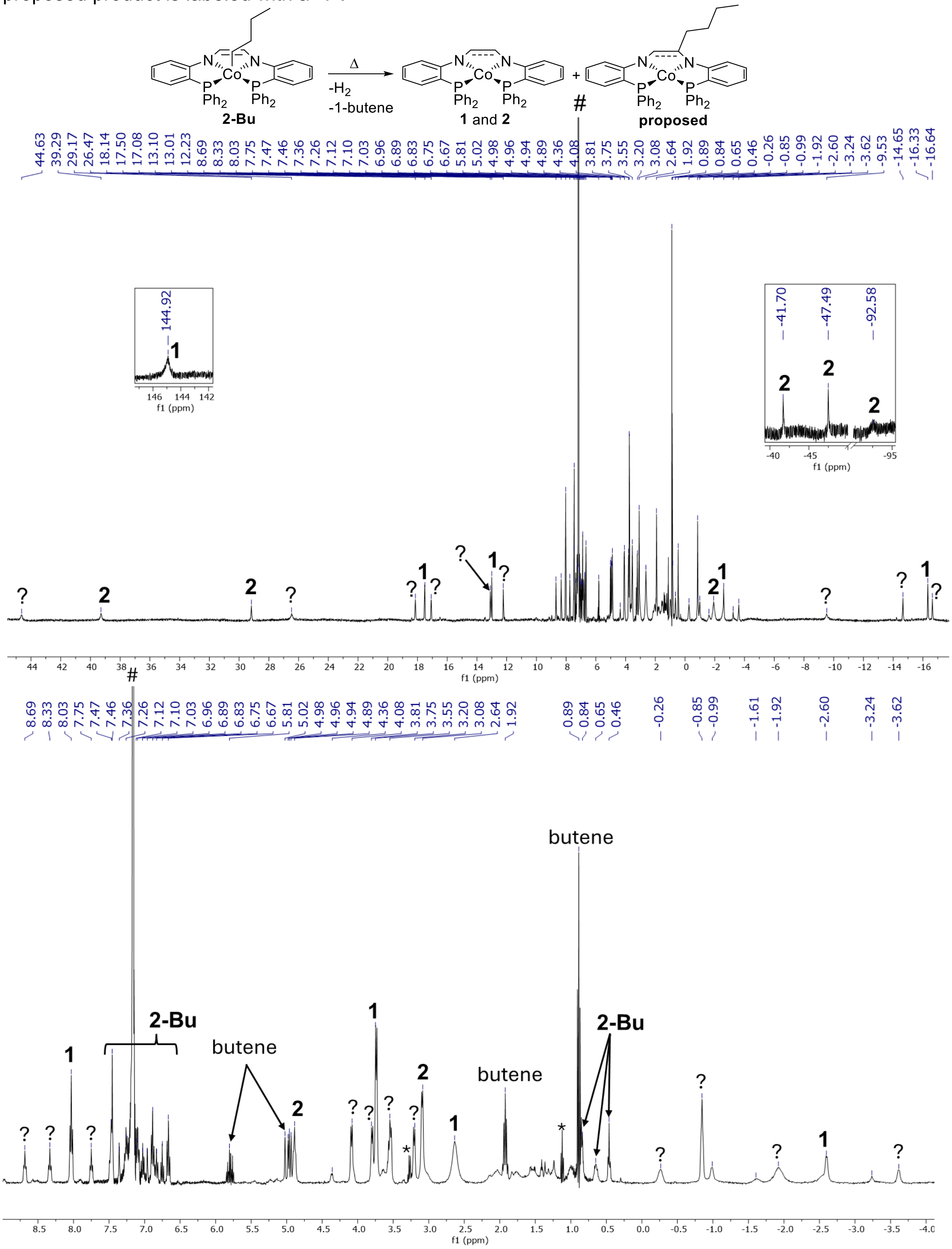

Figure S35. Overlay of final paramagnetic ^{1}H NMR spectra (400 MHz, C_6D_6) for the thermal decomposition of **2-Bn** (top) and **2-Bu** (bottom). Peaks for product **3** are labeled with A, B or C, while the similar peaks observed in the **2-Bu** spectrum are labeled with A', B', or C'.

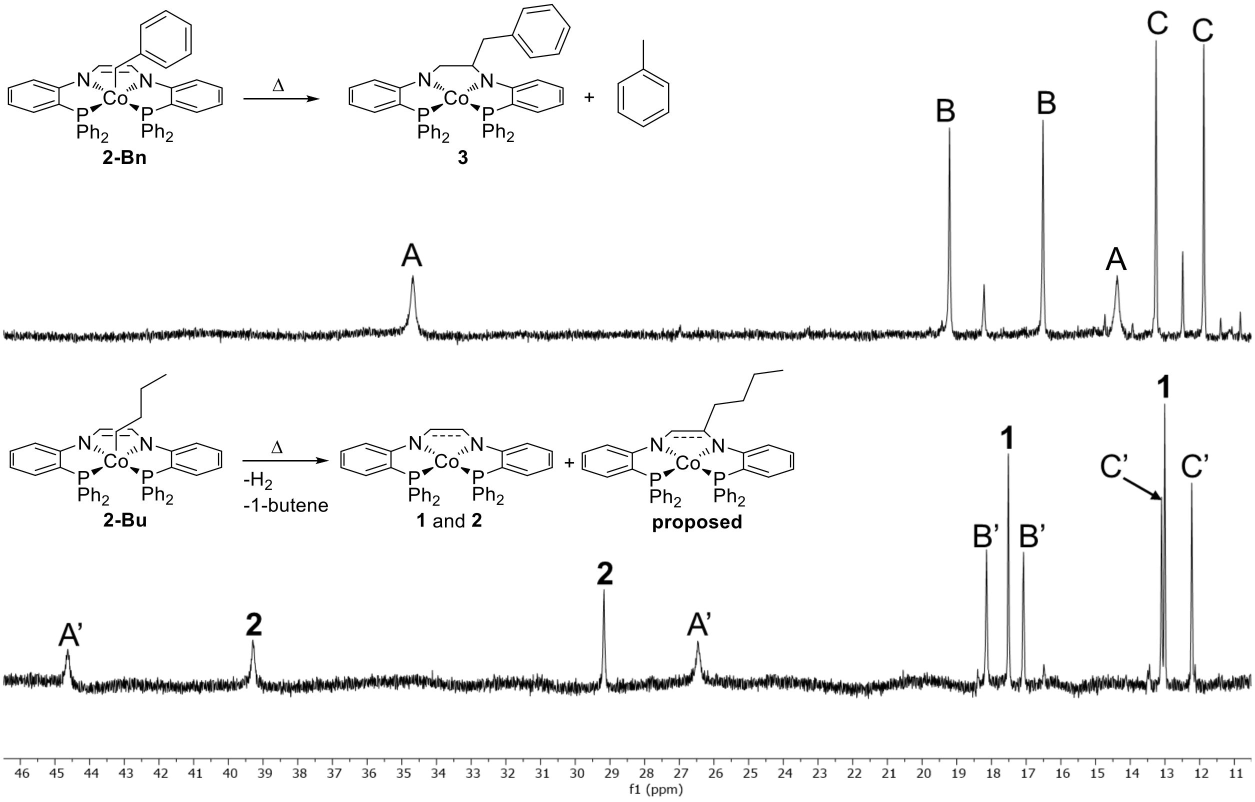

Figure S36. Overlay of final paramagnetic ^{1}H NMR spectra (400 MHz, C_6D_6) for the thermal decomposition of **2-Bn** (top) and **2-Bu** (bottom). Peaks for product **3** are labeled with D or E, while the similar peaks observed in the **2-Bu** spectrum are labeled with D’ or E’. Residual C_6D_5H is labeled with a # and residual Et_2O is labeled with a *.

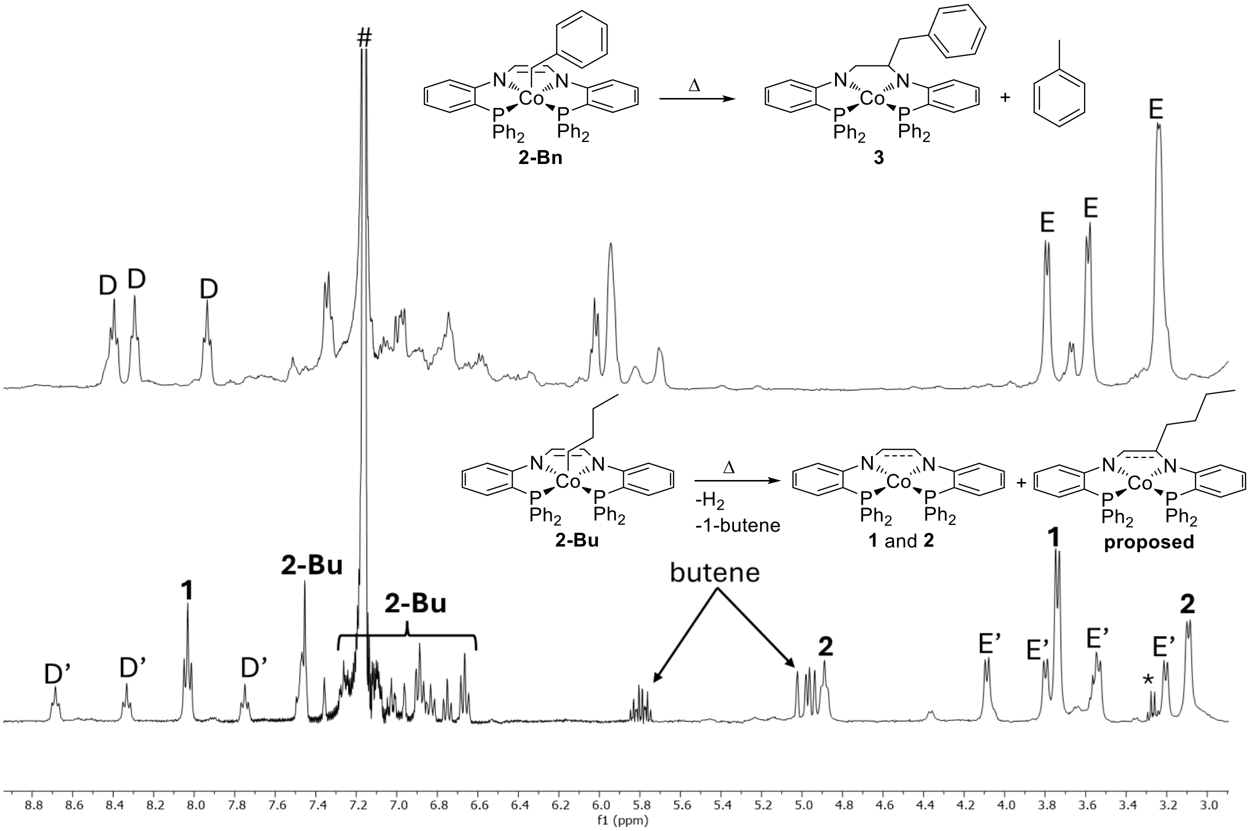

Figure S37. Overlay of final paramagnetic ^{1}H NMR spectra (400 MHz, C_6D_6) for the thermal decomposition of **2-Bn** (top) and **2-Bu** (bottom). Peaks for product **3** are labeled with F or G, while the similar peaks observed in the **2-Bu** spectrum are labeled with F’ or G’.

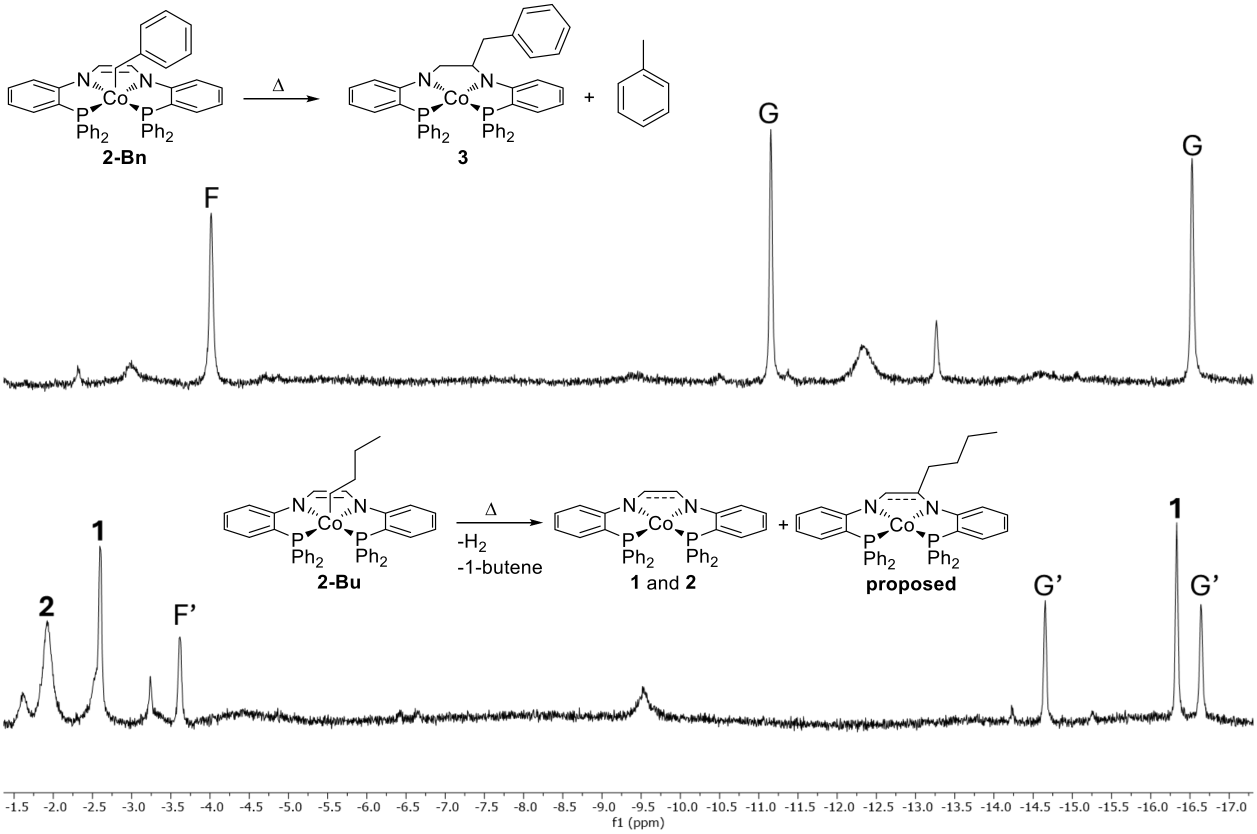

Figure S38. ^{1}H NMR spectra (400 MHz, C_6D_6) of **2-Bn** after thermal decomposition. NMR spectra were taken after initial synthesis (t_0) after 18 h (t_1) and 42 h (t_2) at 50 °C. Residual C_6D_5H is denoted by # and residual bibenzyl is denoted with a *.

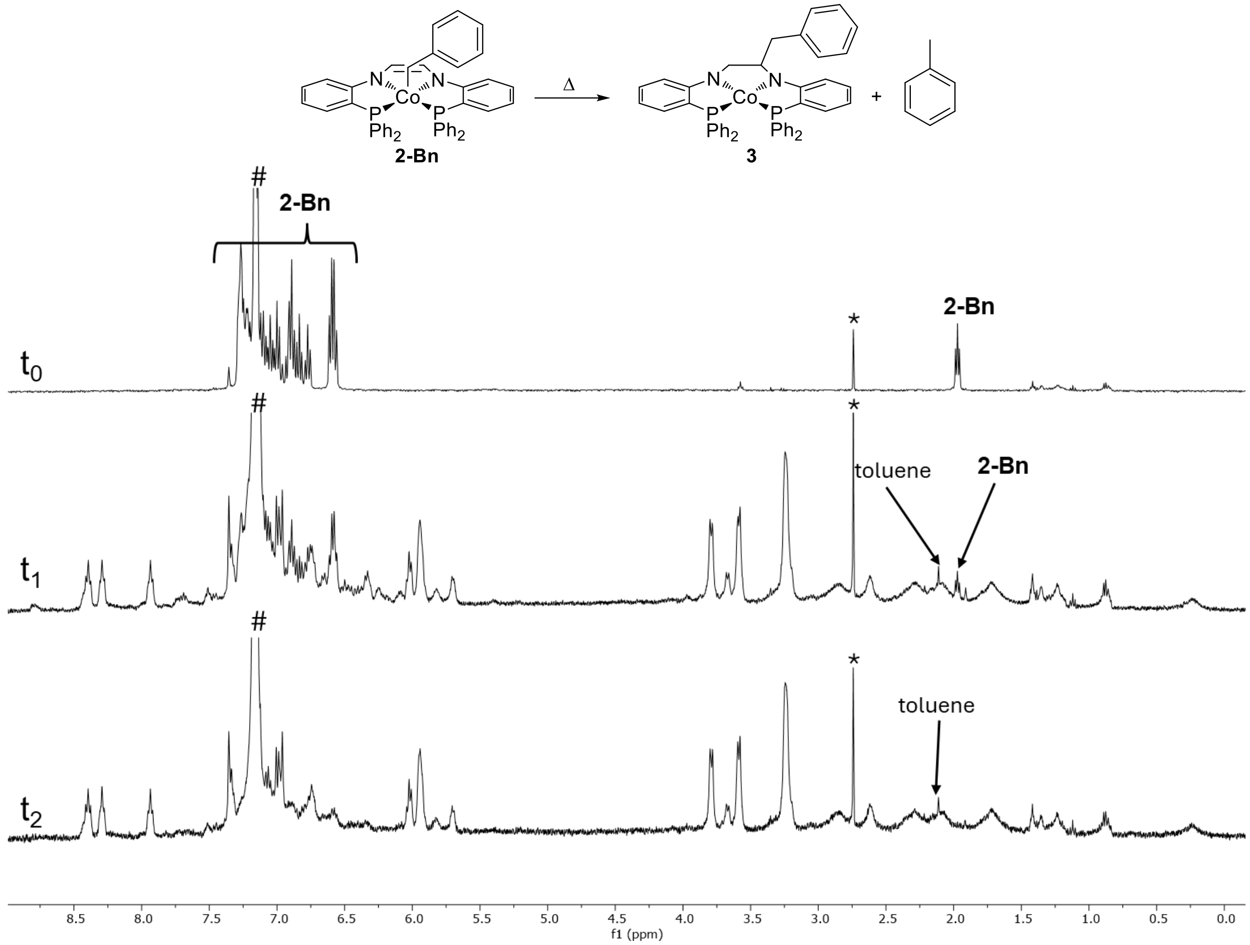

Figure S39. Paramagnetic ^{1}H NMR spectrum (400 MHz, C_6D_6) after the thermal decomposition of **2-Bn**. The labeled peaks correspond to product **3**. Residual bibenzyl from the synthesis of **2-Bn** is identified with a * and residual C_6D_5H is labeled with a #.

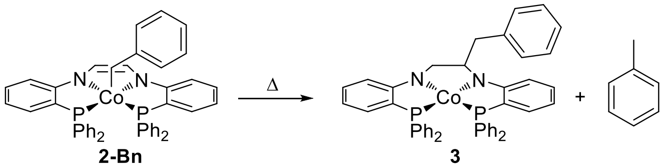

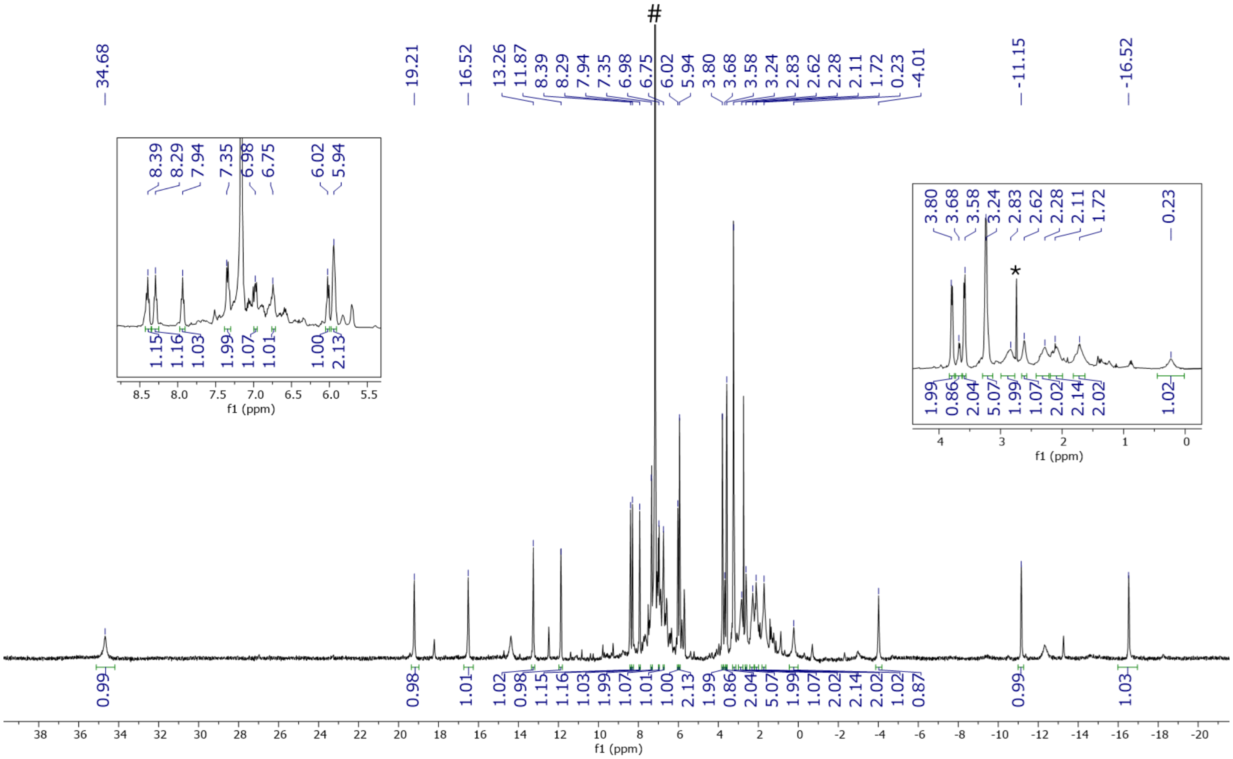

Preliminary reactions of 1-R and 2-R with TEMPO

Figure S40. ^{1}H NMR spectra (400 MHz, C_6D_6) of radical trapping experiment with **1-CH$_3$** and TEMPO at different timepoints at room temperature. Residual Et_2O is denoted with a *. The disappearance of **1** after the 2 d timepoint is likely due to **1** crashing out of solution.

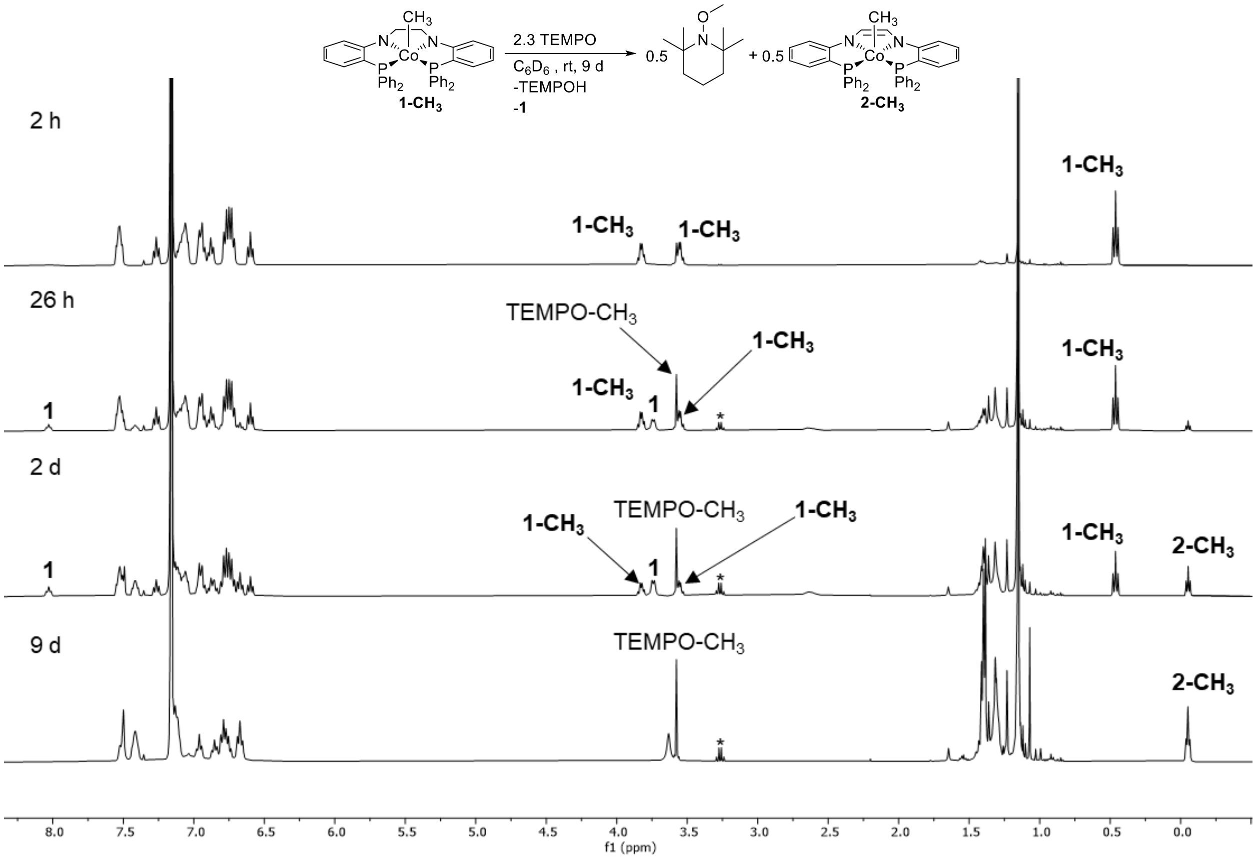

Figure S41. ^{1}H NMR spectra (400 MHz, C_6D_6) of radical trapping experiment with **1-Bu** and TEMPO at different timepoints at room temperature. Residual Et_2O and THF are denoted with a *.

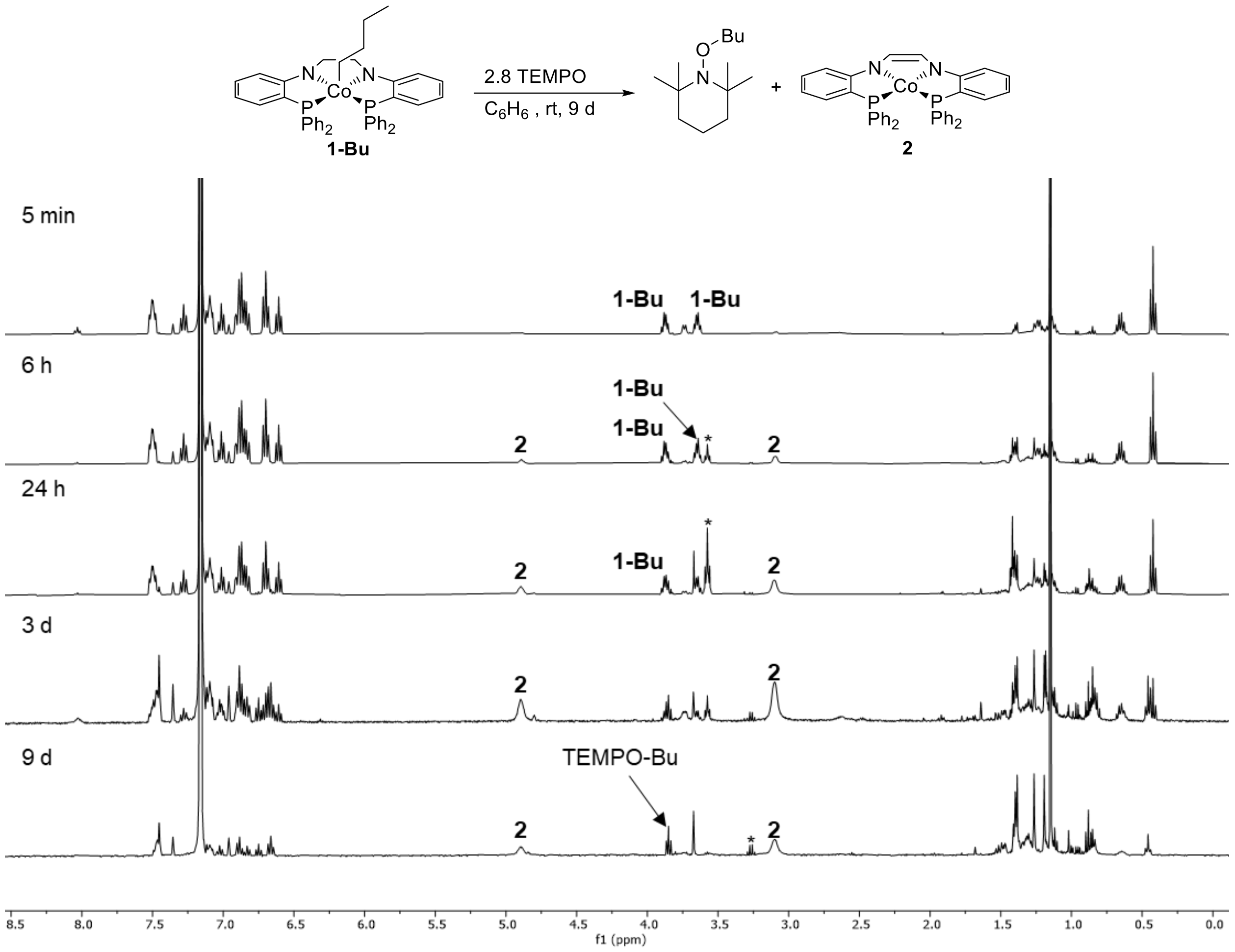

Figure S42. Radical trapping experiment with **1-Bn** and TEMPO at room temperature. ^{1}H NMR spectrum (400 MHz, C_6D_6) was taken after 2 hours of reaction time.

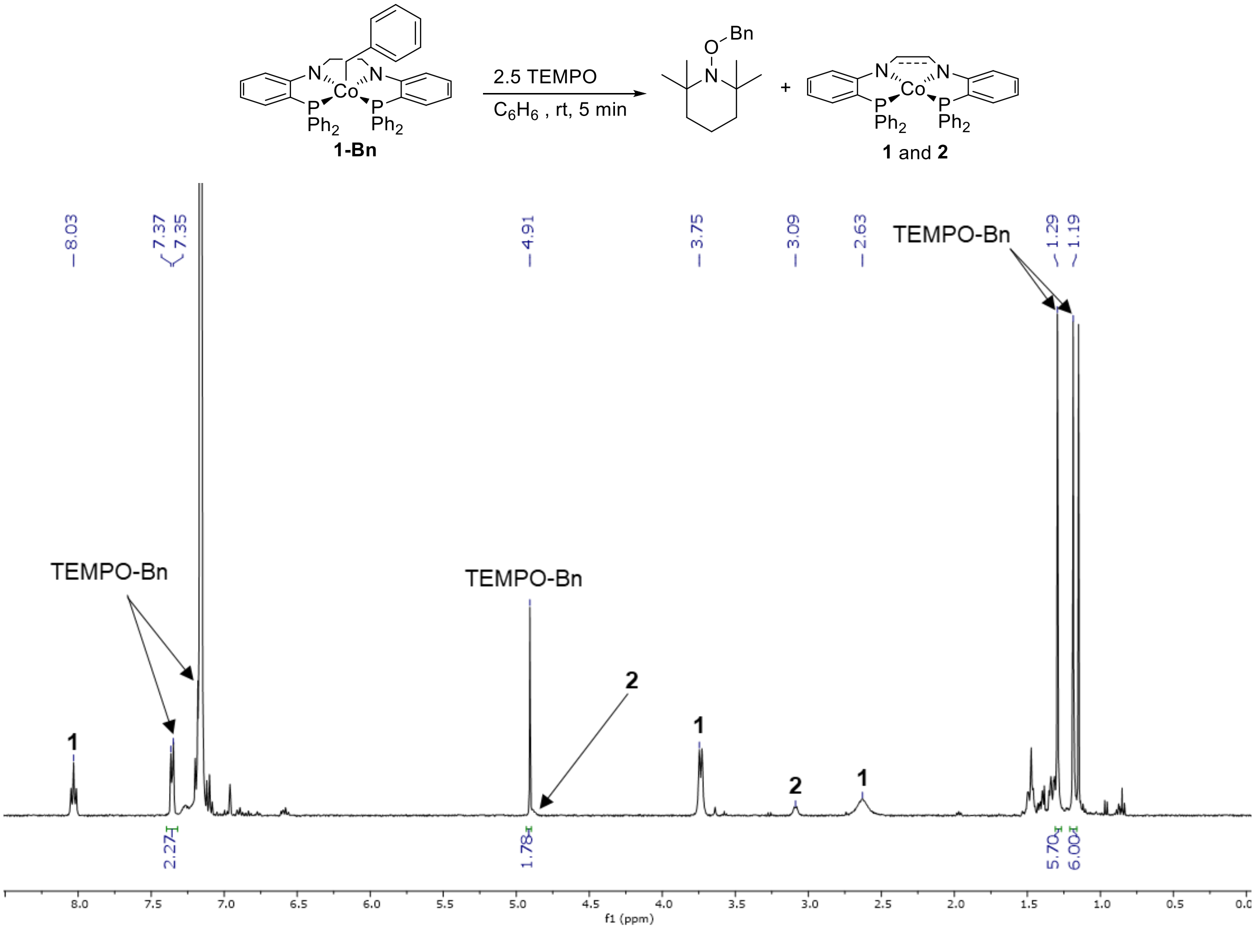

Figure S43. Radical trapping experiment with **2-CH_3** and TEMPO at room temperature. ^{1}H NMR spectrum (400 MHz, C_6D_6) was taken after 14 days of reaction time. Residual Et_2O is denoted with a *.

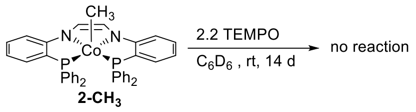

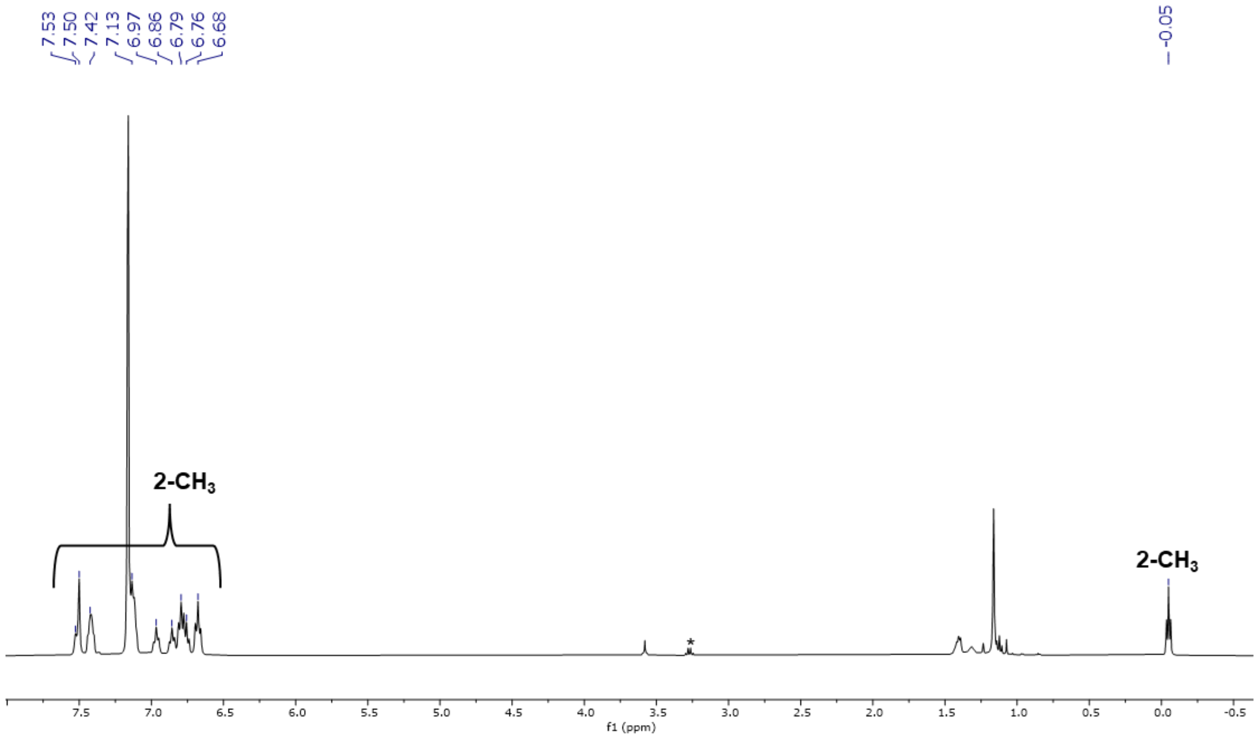

Figure S44. ^{1}H NMR spectra (400 MHz, C_6D_6) of radical trapping experiment with **2-Bu** and TEMPO at different timepoints at room temperature. Residual Et_2O is denoted with a *.

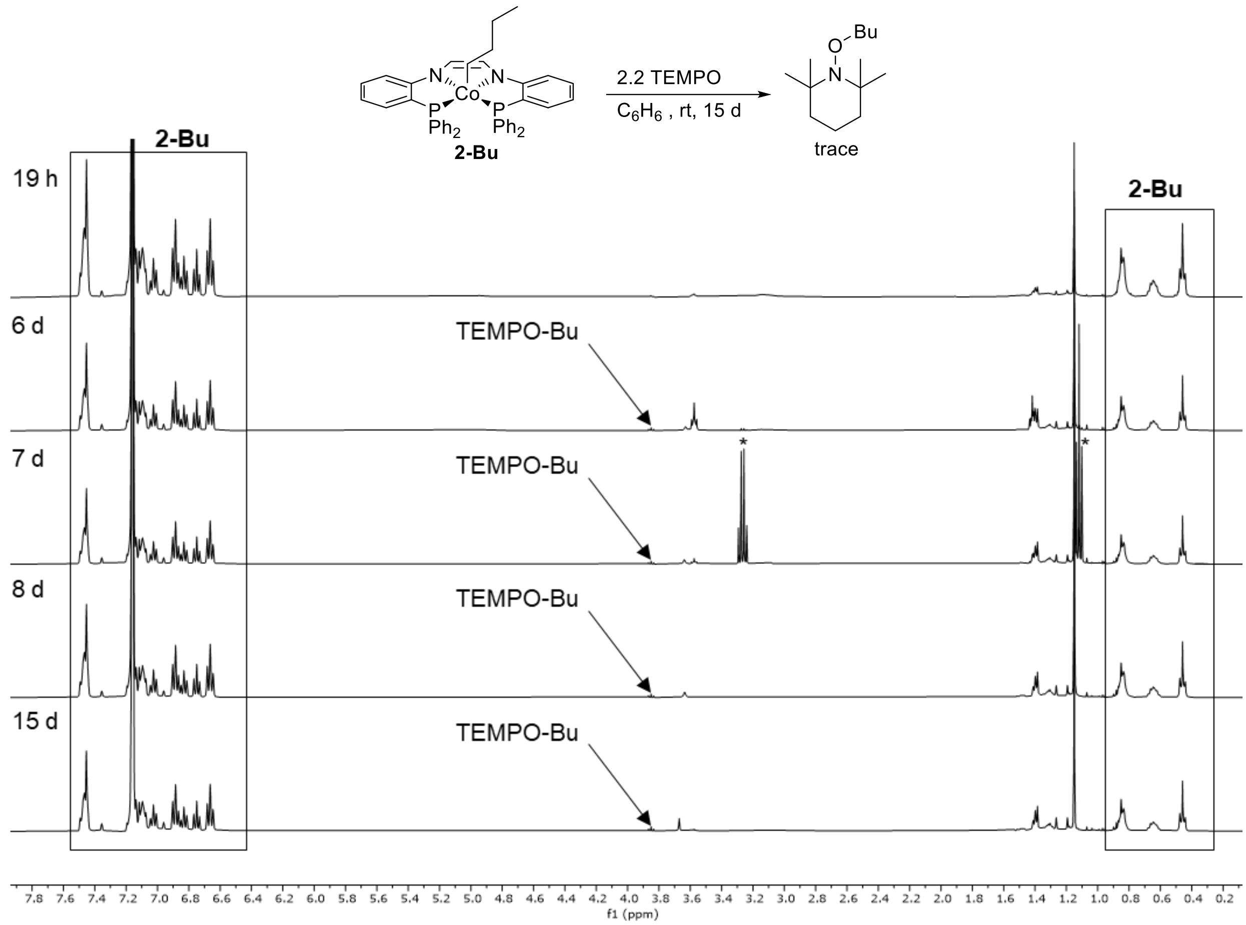

Figure S45. ¹H NMR spectra (400 MHz, C_6D_6) of radical trapping experiment with **2-Bn** and TEMPO at different timepoints at room temperature. Residual THF is denoted with a *.

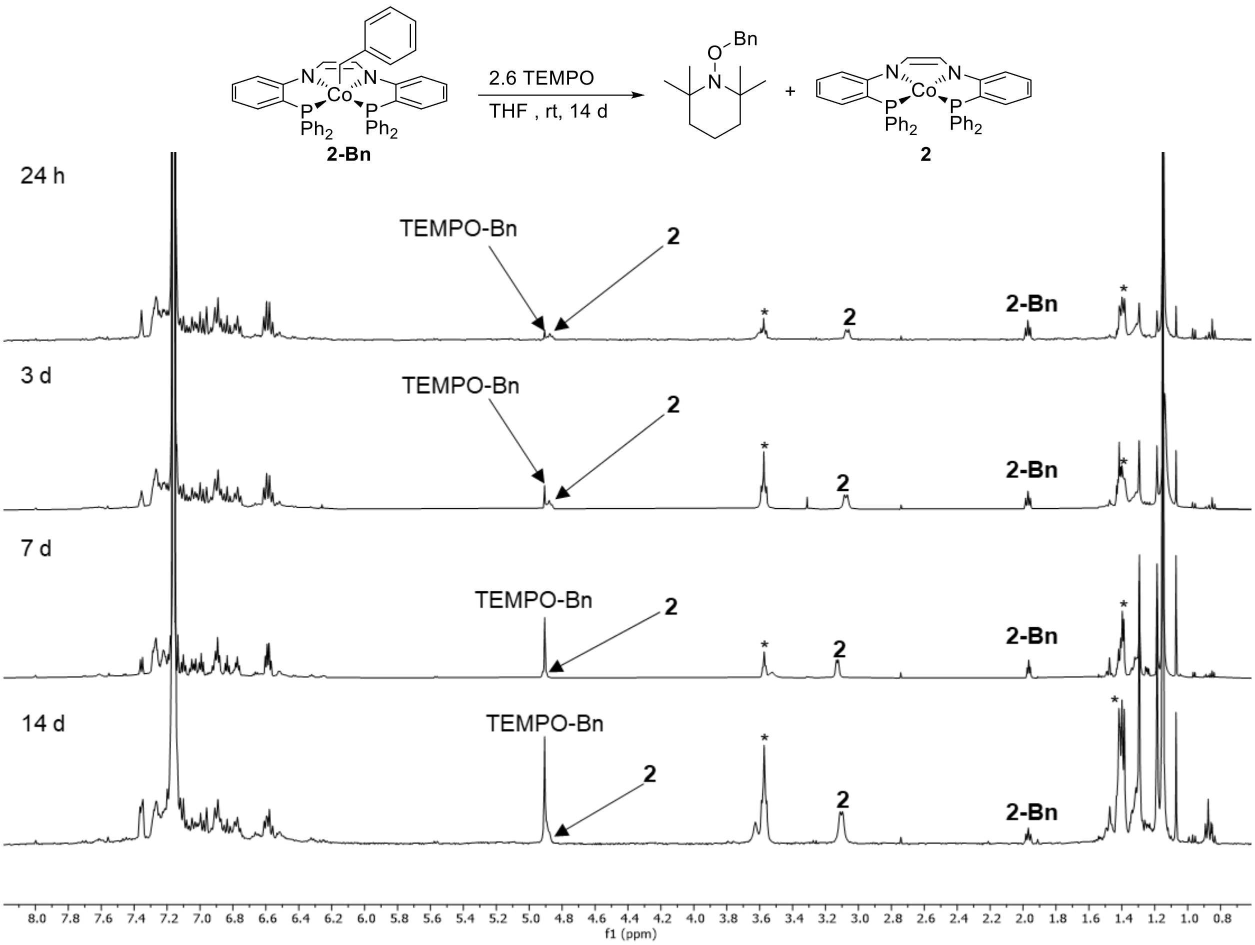

Eyring Plot Experiments

Eyring plot kinetics at 34 °C. 2-Bn (3.5 mg, 0.0048 mmol) and TEMPO (14.9 mg, 0.0954 mmol) were dissolved in 0.75 mL C_6D_6 then filtered into a J. Young tube containing 3.0 µL of a hexamethylbenzene solution (0.39 M in C_6H_6). The J. Young tube was immediately sealed and a quantitative ^{1}H NMR spectrum was obtained within 20 minutes (t_0). The J. Young tube was then placed in an aluminum block heated to 34.4±0.2 °C in the dark. The reaction was monitored by quantitative ^{1}H NMR spectroscopy every 12-24 hours and the concentration of **2-Bn** at each timepoint was determined using hexamethylbenzene as an internal standard.

Eyring plot kinetics at 41 °C. 2-Bn (3.6 mg, 0.0050 mmol) and TEMPO (15.1 mg, 0.0966 mmol) were dissolved in 0.75 mL C_6D_6 then filtered into a J. Young tube containing 3.0 µL of a hexamethylbenzene solution (0.39 M in C_6H_6). The J. Young tube was immediately sealed and a quantitative ^{1}H NMR spectrum was obtained within 20 minutes (t_0). The J. Young tube was then placed in an aluminum block heated to 41.4±0.1 °C in the dark. The reaction was monitored by quantitative ^{1}H NMR spectroscopy every 4-6 hours and the concentration of **2-Bn** at each timepoint was determined using hexamethylbenzene as an internal standard.

Eyring plot kinetics at 47 °C. 2-Bn (3.2 mg, 0.0044 mmol) and TEMPO (14.2 mg, 0.0909 mmol) were dissolved in 0.75 mL C_6D_6 then filtered into a J. Young tube containing 3.0 µL of a hexamethylbenzene solution (0.39 M in C_6H_6). The J. Young tube was immediately sealed and taken to a pre-heated (47.0 °C) 400 MHz NMR spectrometer and quantitative ^{1}H NMR spectra were obtained every 15-60 minutes. The concentration of **2-Bn** at each timepoint was determined using hexamethyl benzene as an internal standard.

Eyring plot kinetics at 52 °C. 2-Bn (3.3 mg, 0.0045 mmol) and TEMPO (14.2 mg, 0.0909 mmol) were dissolved in 0.75 mL C_6D_6 then filtered into a J. Young tube containing 3.0 µL of a hexamethylbenzene solution (0.39 M in C_6H_6). The J. Young tube was immediately sealed and taken to a pre-heated (52.0 °C) 400 MHz NMR spectrometer and quantitative ^{1}H NMR spectra were obtained every 5-30 minutes. The concentration of **2-Bn** at each timepoint was determined using hexamethyl benzene as an internal standard.

Eyring plot kinetics at 58 °C. 2-Bn (3.2 mg, 0.0044 mmol) and TEMPO (14.0 mg, 0.0896 mmol) were dissolved in 0.75 mL C_6D_6 then filtered into a J. Young tube containing 3.0 µL of a hexamethylbenzene solution (0.39 M in C_6H_6). The J. Young tube was immediately sealed and taken to a pre-heated (58.0 °C) 400 MHz NMR spectrometer and quantitative ^{1}H NMR spectra were obtained every 7-15 minutes. The concentration of **2-Bn** at each timepoint was determined using hexamethyl benzene as an internal standard.

Figure S46. Plots of [**2-Bn**] vs time at 34.4 °C, 41.4 °C, 47.0 °C, 52.0 °C, and 58.0 °C. Concentrations were determined through quantitative ^{1}H NMR spectroscopy using hexamethylbenzene as an internal standard.

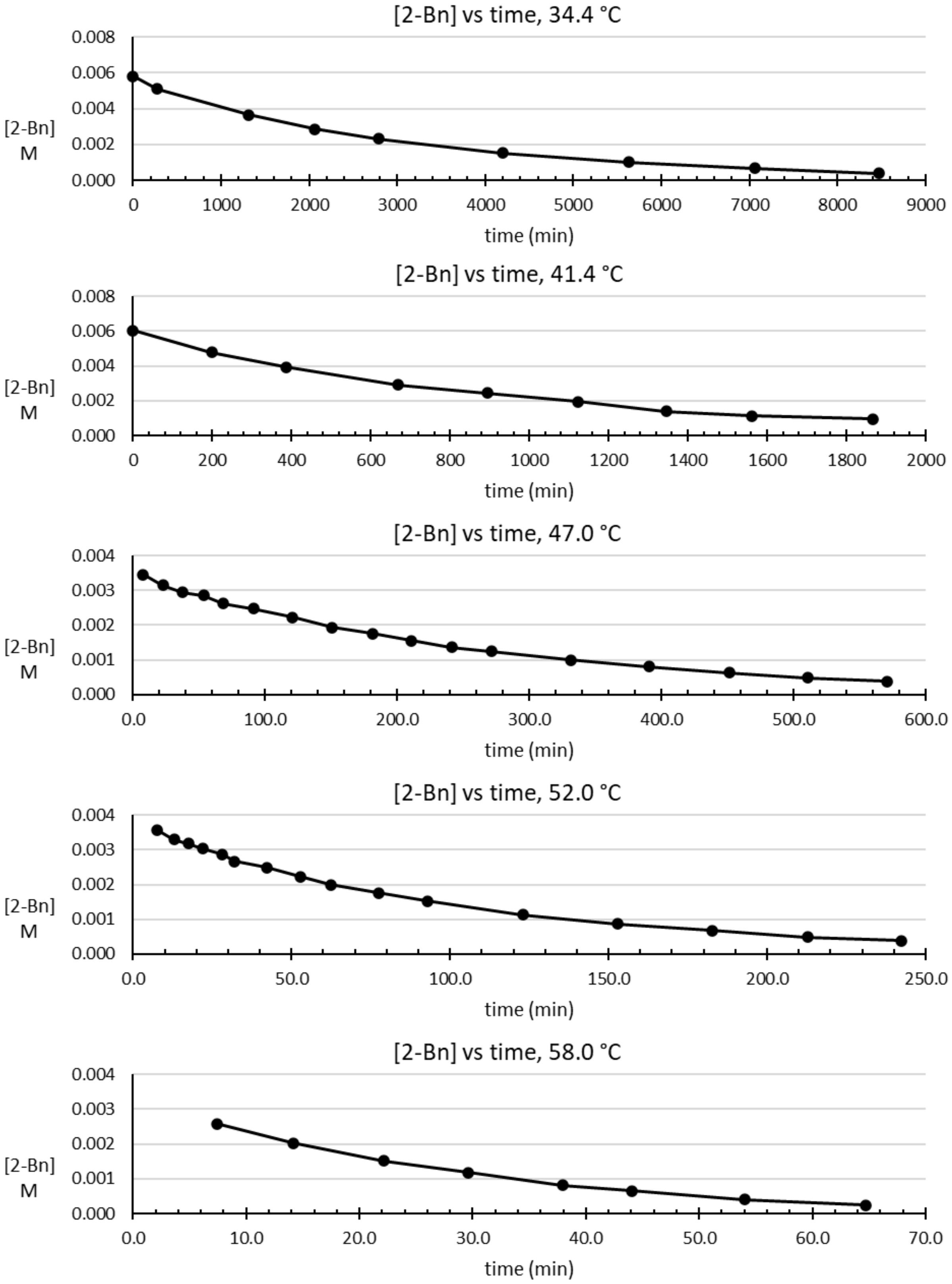

Figure S47. Plots of ln[**2-Bn**] vs time at 34.4 °C, 41.4 °C, 47.0 °C, 52.0 °C, and 58.0 °C. Concentrations were determined through quantitative ^{1}H NMR spectroscopy using hexamethylbenzene as an internal standard.

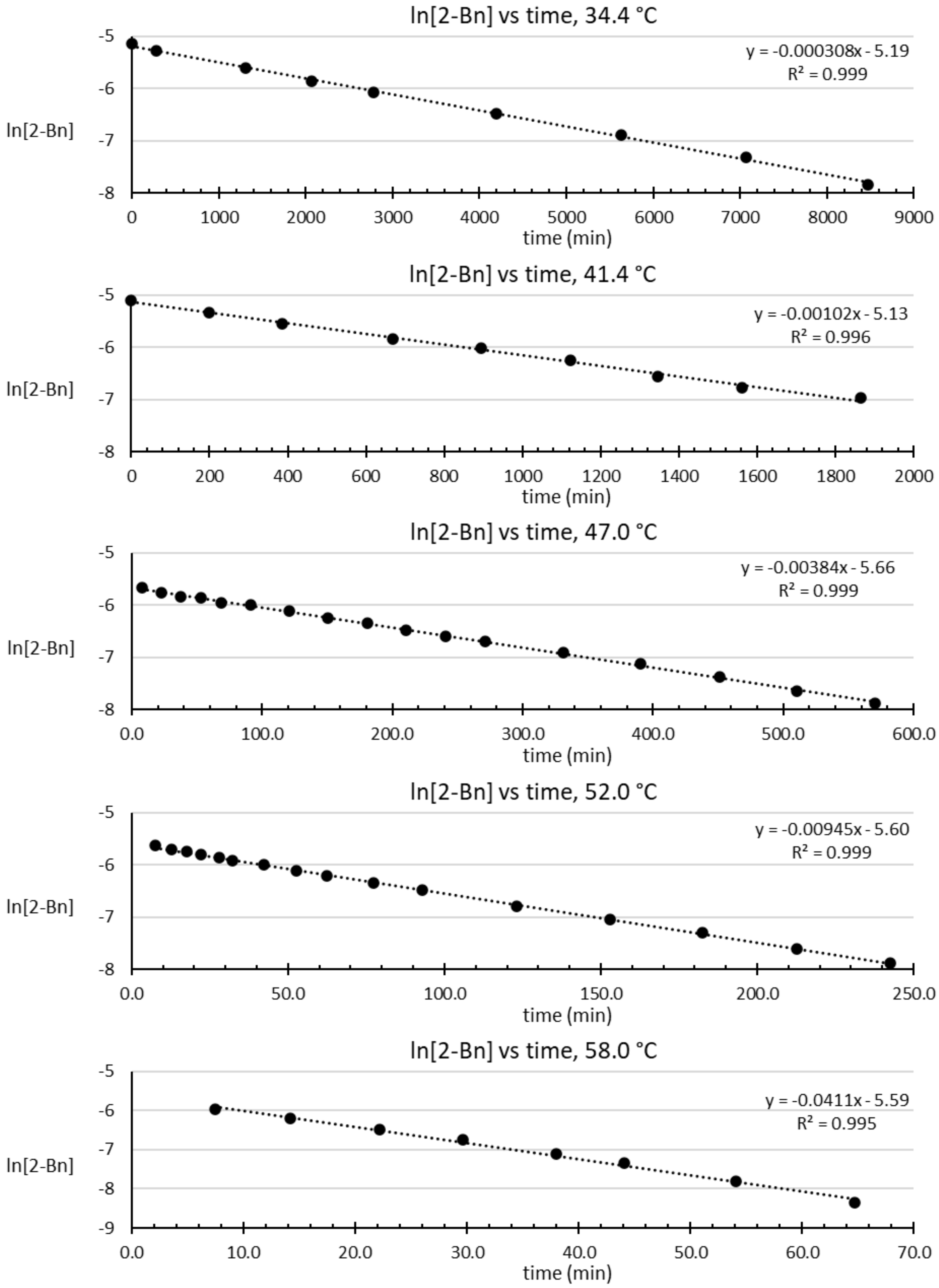

Figure S48. Representative quantitative ^{1}H NMR spectra (400 MHz, C_6D_6) for the reaction of **2-Bn** with 20 equivalents of TEMPO at 34.4±0.2 °C. The top spectrum is the initial (t_0) timepoint, while the bottom spectrum shows the final timepoint (t_8, 8474 min).

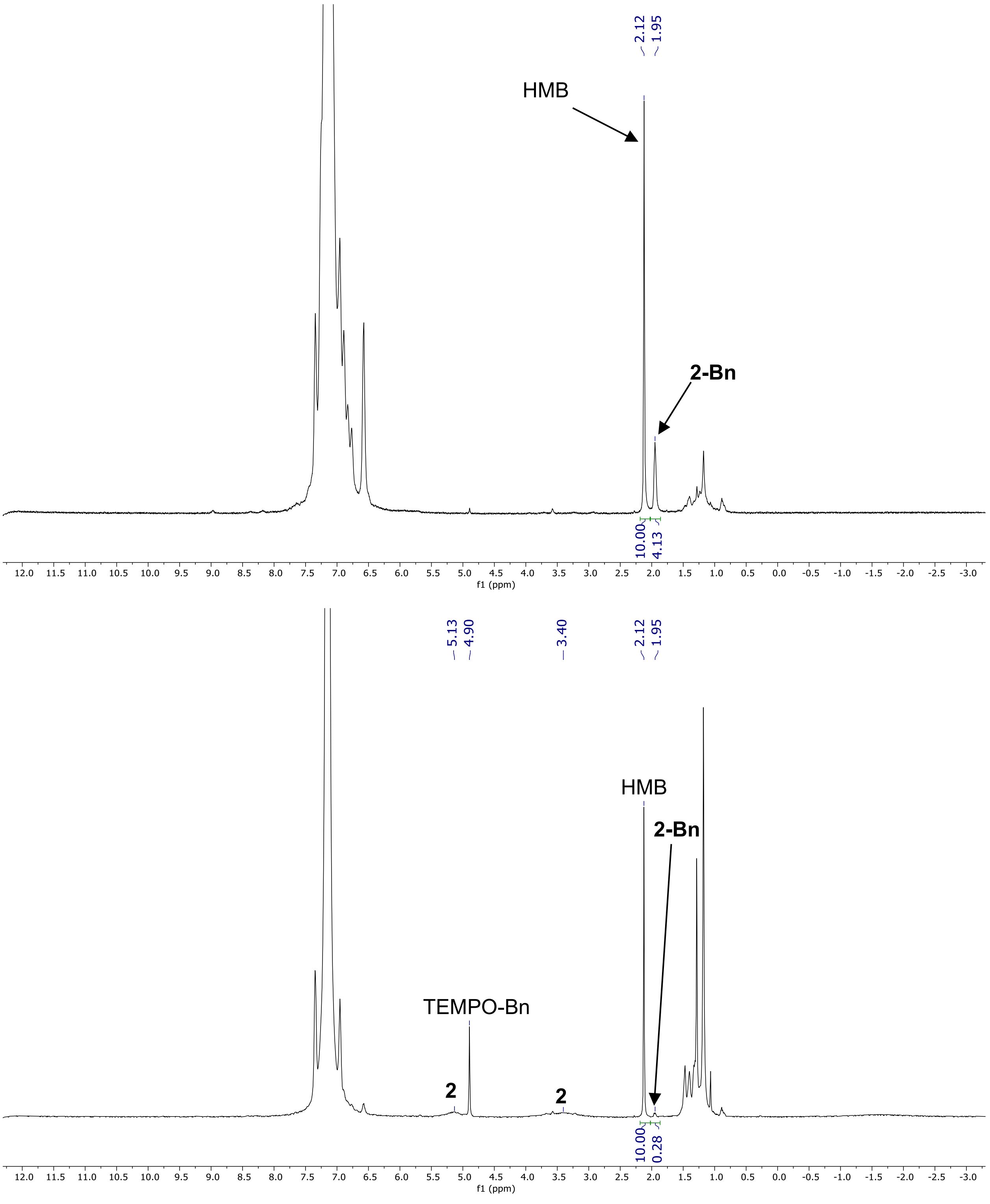

Catalysis

Scheme S1. General scheme for catalytic reactions.

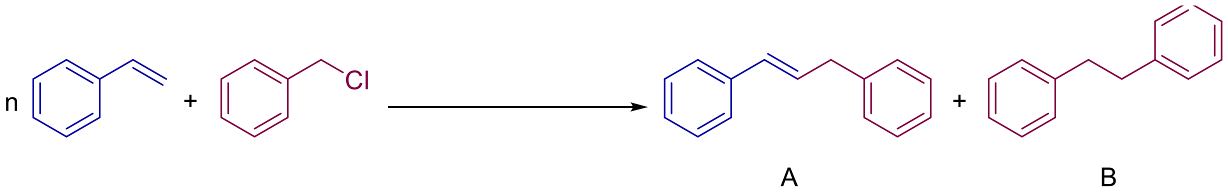

Figure S49. ^{1}H NMR spectrum post-catalysis with **1** (400 MHz, C_6D_6) used for response factor calculation of **A** and **B,** using 1,3,5-trimethoxybenzene (TMB) as an internal standard.

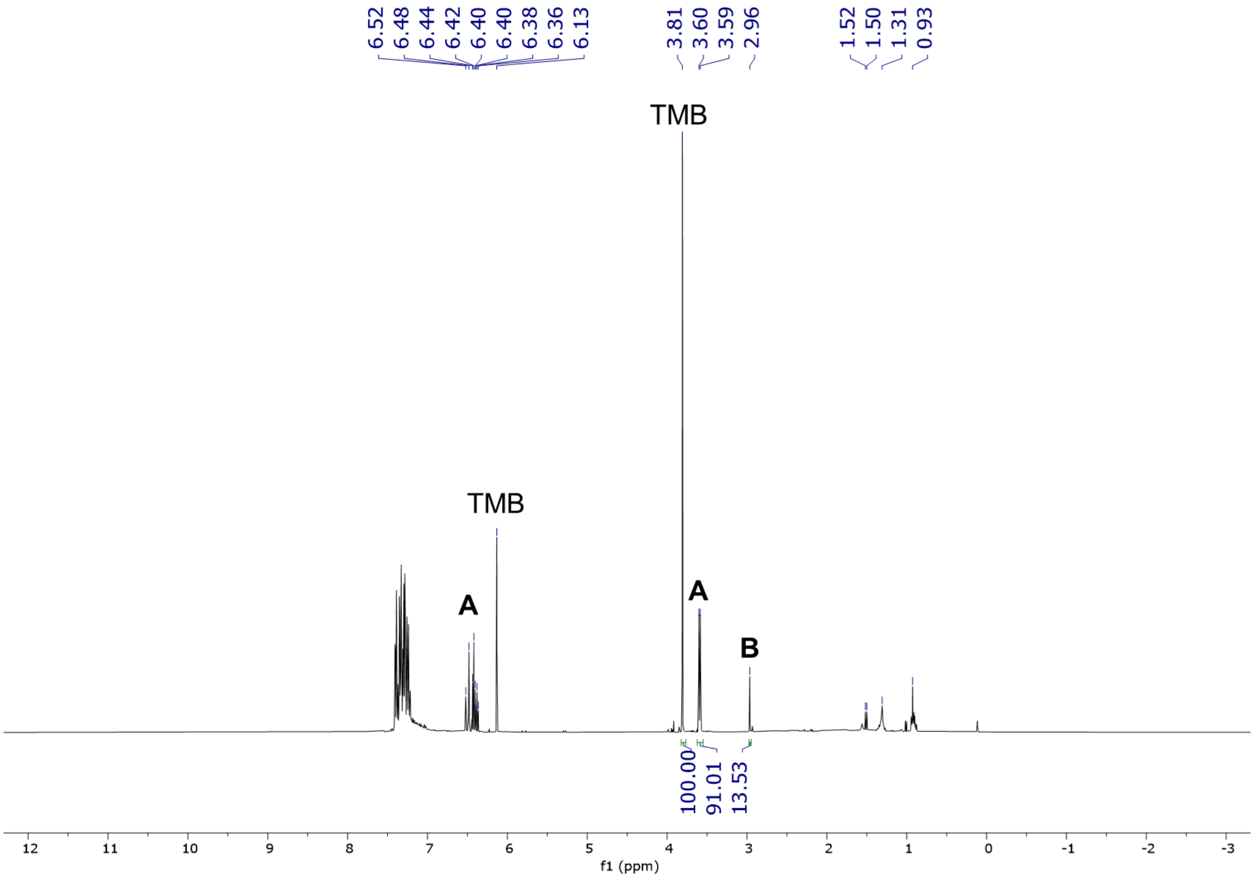

Figure S50. Gas chromatogram (FID) post-catalysis with **1** used for response factor calculation of **A** and **B** with respect to TMB**.**

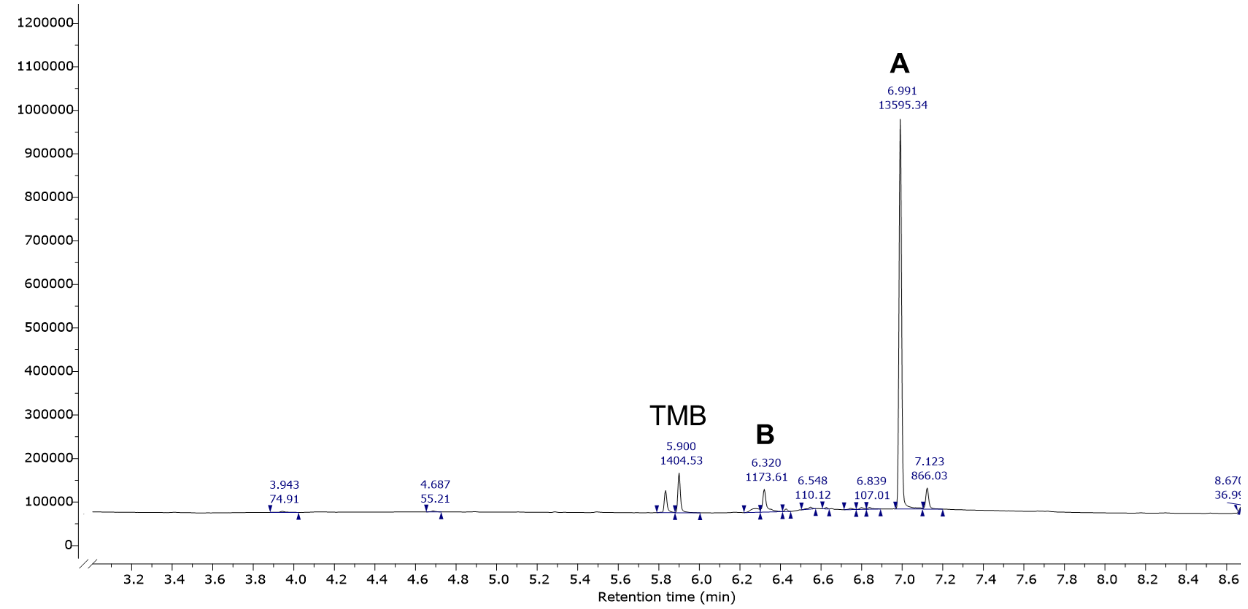

Figure S51. Representative gas chromatogram (FID) of post-catalytic mixture**.**

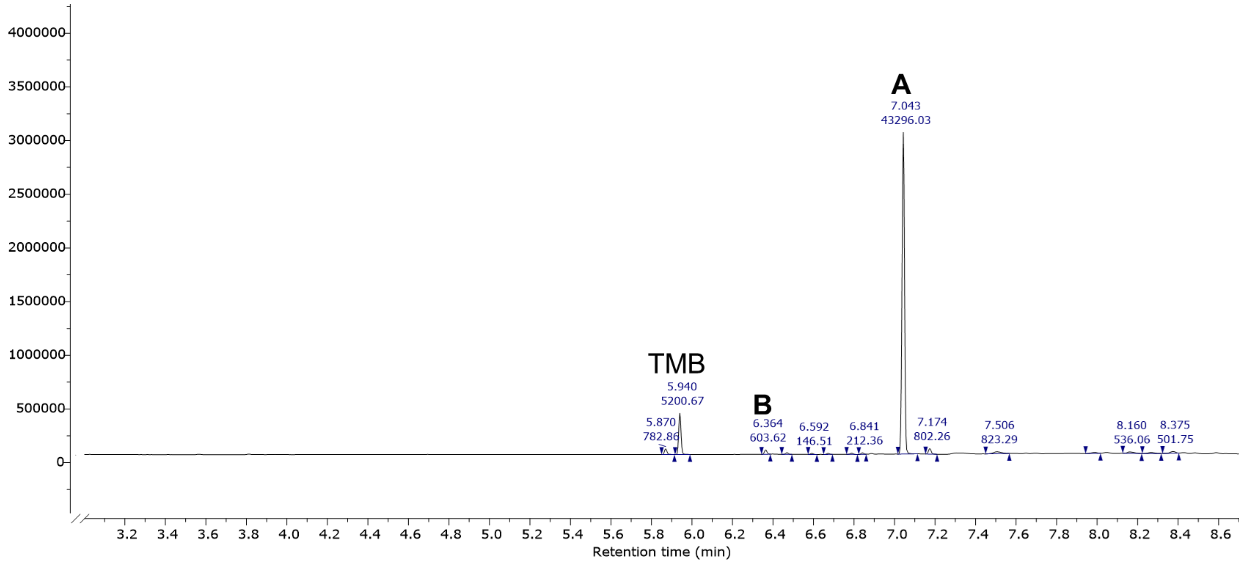

Figure S52. Mass spectrum of 1,3,5-trimethoxybenzene, TMB.

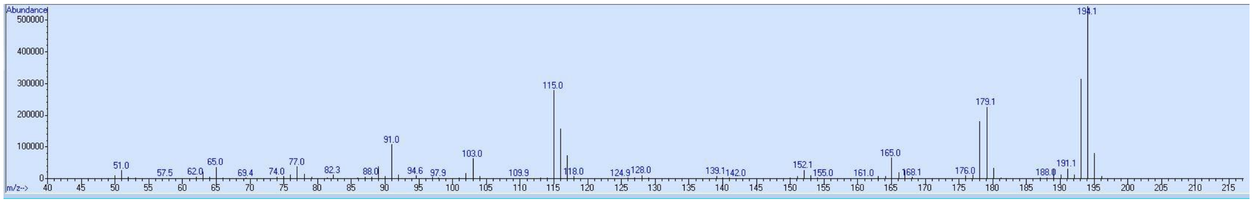

Figure S53. Mass spectrum of cross-coupled product, **A**.

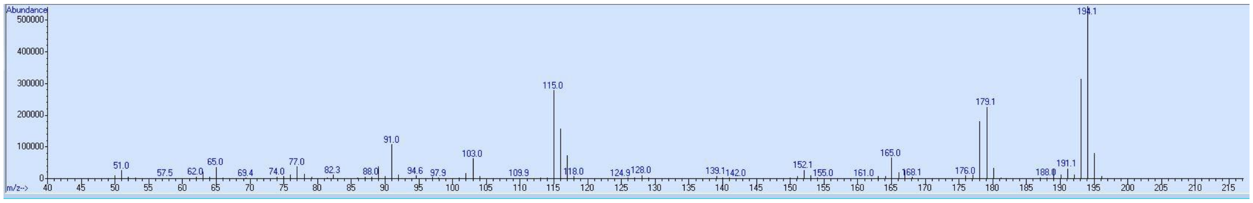

Figure S54. Mass Spectrum of bibenzyl, **B**.

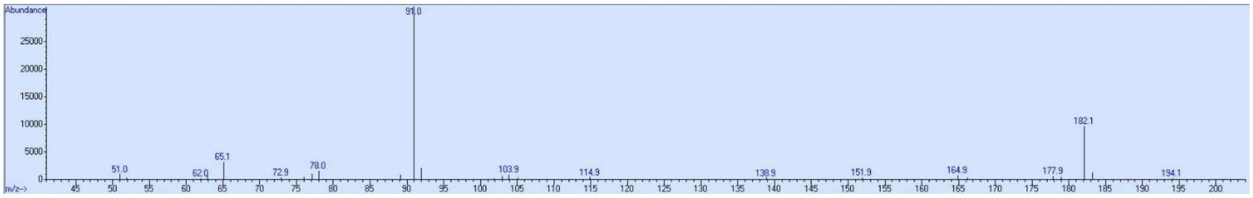

Figure S55. ^{1}H NMR spectrum (400 MHz, C_6D_6) obtained following the reaction of **1-Bn** and styrene. Residual C_6D_5H is detonated with a #. Residual THF is designated with a *. Residual pentane denoted with a %.

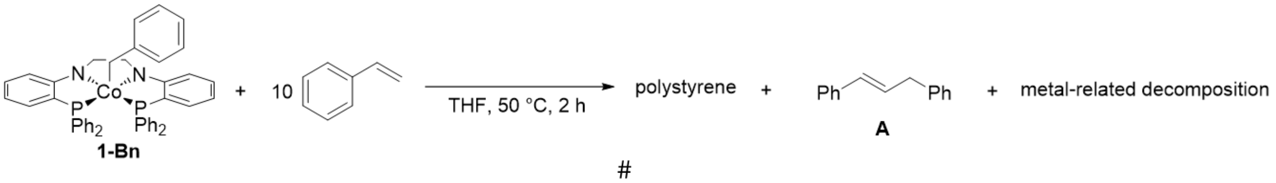

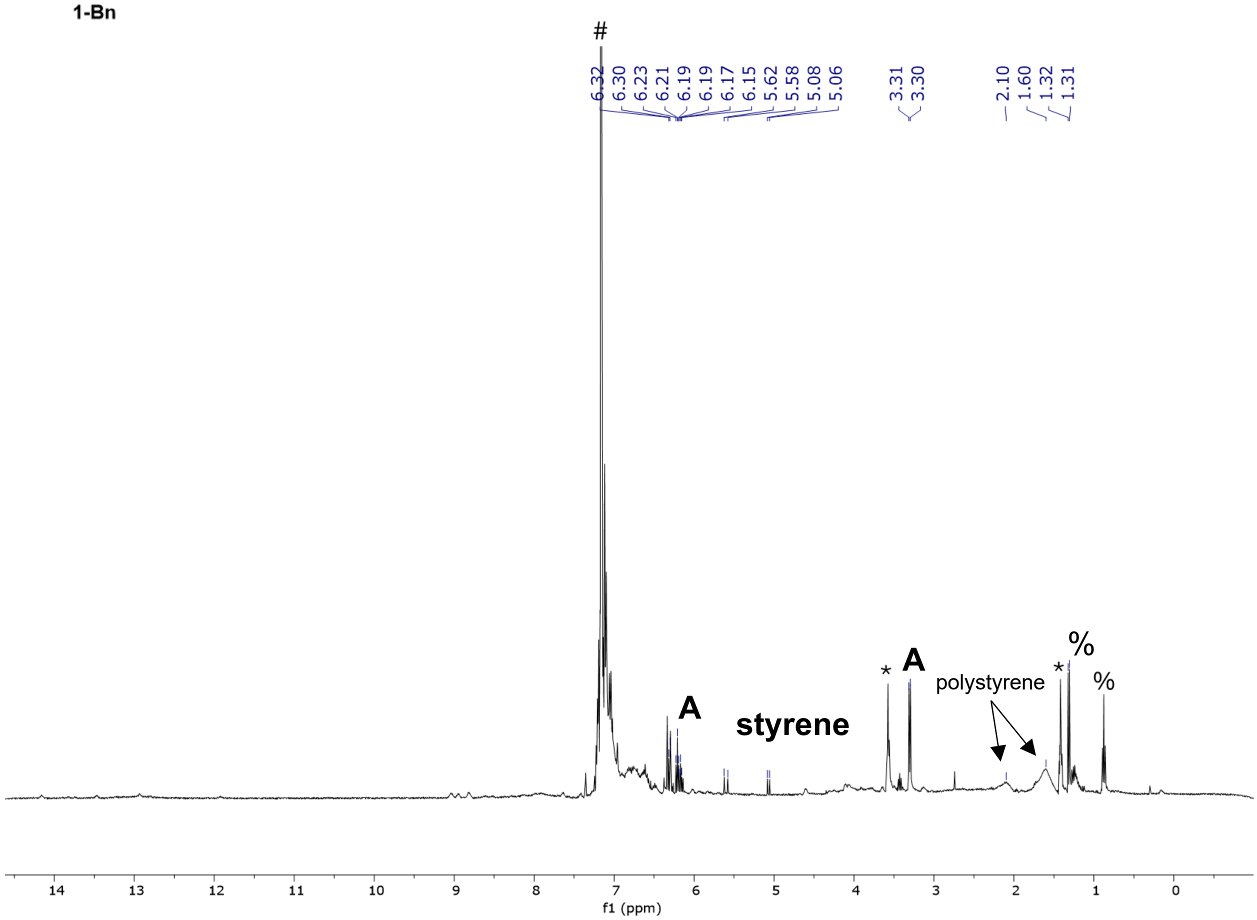

Figure S56. ^{1}H NMR spectrum (400 MHz, C_6D_6) of the post-catalytic mixture showing formation of polystyrene. Residual toluene denoted by a #.

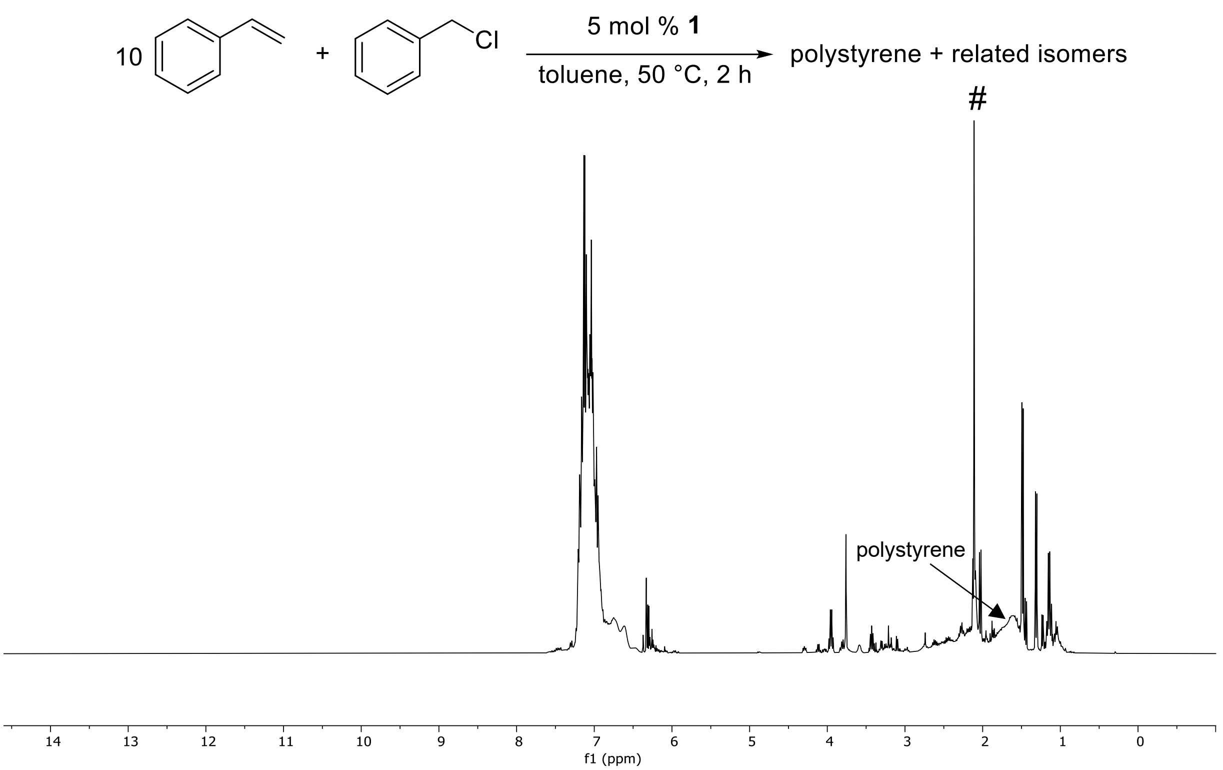

Discussion of catalytic vs homolysis product distribution for 1-Bn/1:

When **1** (**1-Bn** being the active catalyst) is used as a catalyst for the radical Heck-type cross coupling of styrene and BnCl a significant amount of bibenzyl is observed, while during homolysis of **1-Bn** bibenzyl is not observed and toluene is the major organic product. This discrepancy is likely due to the differences in the instantaneous concentration of benzyl radicals in solution. For the homolysis and the catalytic experiments, the overall concentrations of metal complex are similar (3-5 mM for the homolysis experiments, 5 mM for catalysis). The increased temperature for the catalytic experiments (50 °C) compared to the homolysis experiment (rt) would result in an increased rate of Co-C bond homolysis for **1-Bn**, effectively increasing the concentration of benzyl radicals in solution. Additionally, the Zn and BnCl present in the catalytic reaction would re-form any **1-Bn** that underwent Co-C bond homolysis, resulting in a relatively constant concentration of **1-Bn** during the reaction. For these reasons the concentration of benzyl radicals in solution for the catalytic experiments would be higher than the homolysis experiment, increasing the likelihood of bibenzyl formation. For the homolysis experiment, the instantaneous concentration of benzyl radicals in solution is low compared to the catalytic reactions and the benzyl radicals are more likely to abstract an H-atom from the bulk solution rather than find another benzyl radical in solution to produce bibenzyl.

GC Traces for Catalytic Trials

Figure S57. GC / FID data for Table 3, entry 1 in the main text. Conditions: 5 mM / 5 mol % **1**, 1 equivalent of styrene, 1 equivalent benzyl chloride, 2 h reaction time, 2 equivalents Zn, 50 °C, THF as solvent. TMB was used as an internal standard.

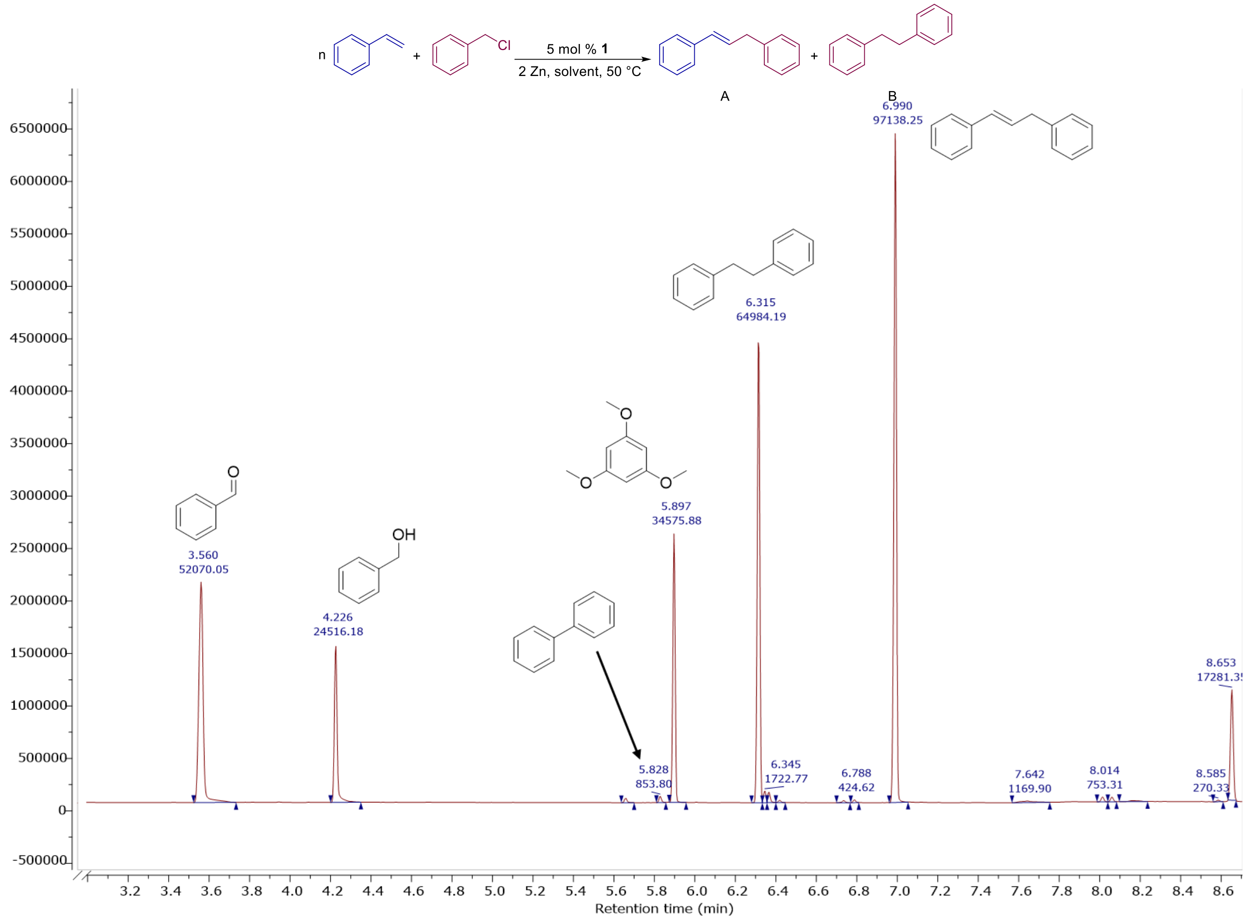

Figure S58. GC / FID data for Table 3, entry 2 in the main text. <u>Conditions:</u> 5 mM / 5 mol % **1**, 5 equivalents of styrene, 1 equivalent benzyl chloride, 2 equivalents Zn, 2 h reaction time, 50 °C, THF as solvent. TMB was used as an internal standard.

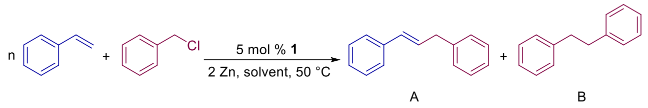

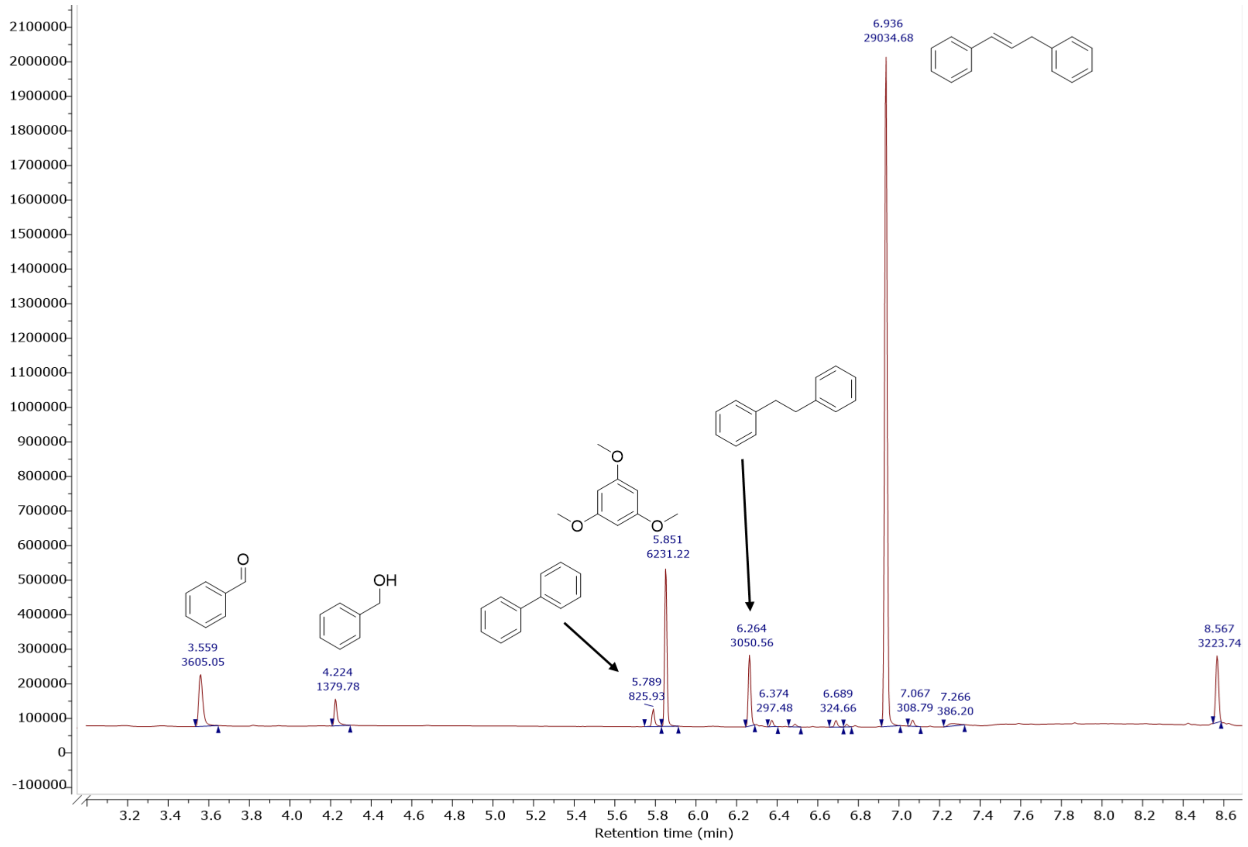

Figure S59. GC / FID data for Table 3, entry 3 in the main text. Conditions: 5 mM / 5 mol % **1**, 10 equivalents of styrene, 1 equivalent benzyl chloride, 2 equivalents Zn, 2 h reaction time, 50 °C, THF as solvent. TMB was used as an internal standard.

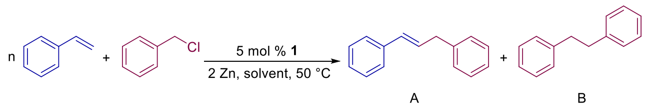

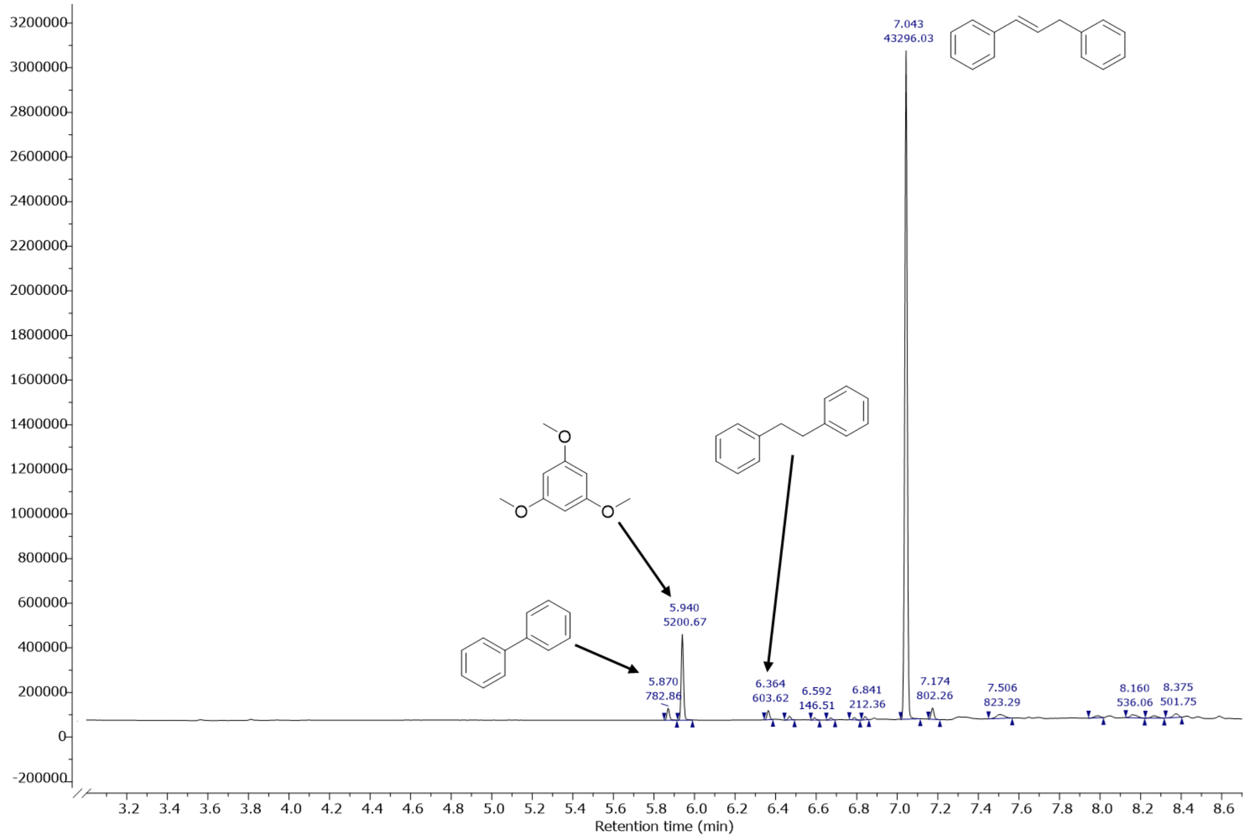

Figure S60. GC / FID data for Table 3, entry 4 in the main text. Conditions: 5 mM / 5 mol % **1**, 20 equivalents of styrene, 1 equivalent benzyl chloride, 2 equivalents Zn, 2 h reaction time, 50 °C, THF as solvent. TMB was used as an internal standard.

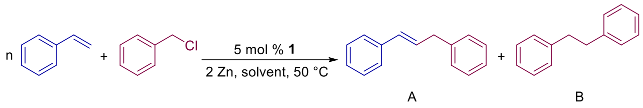

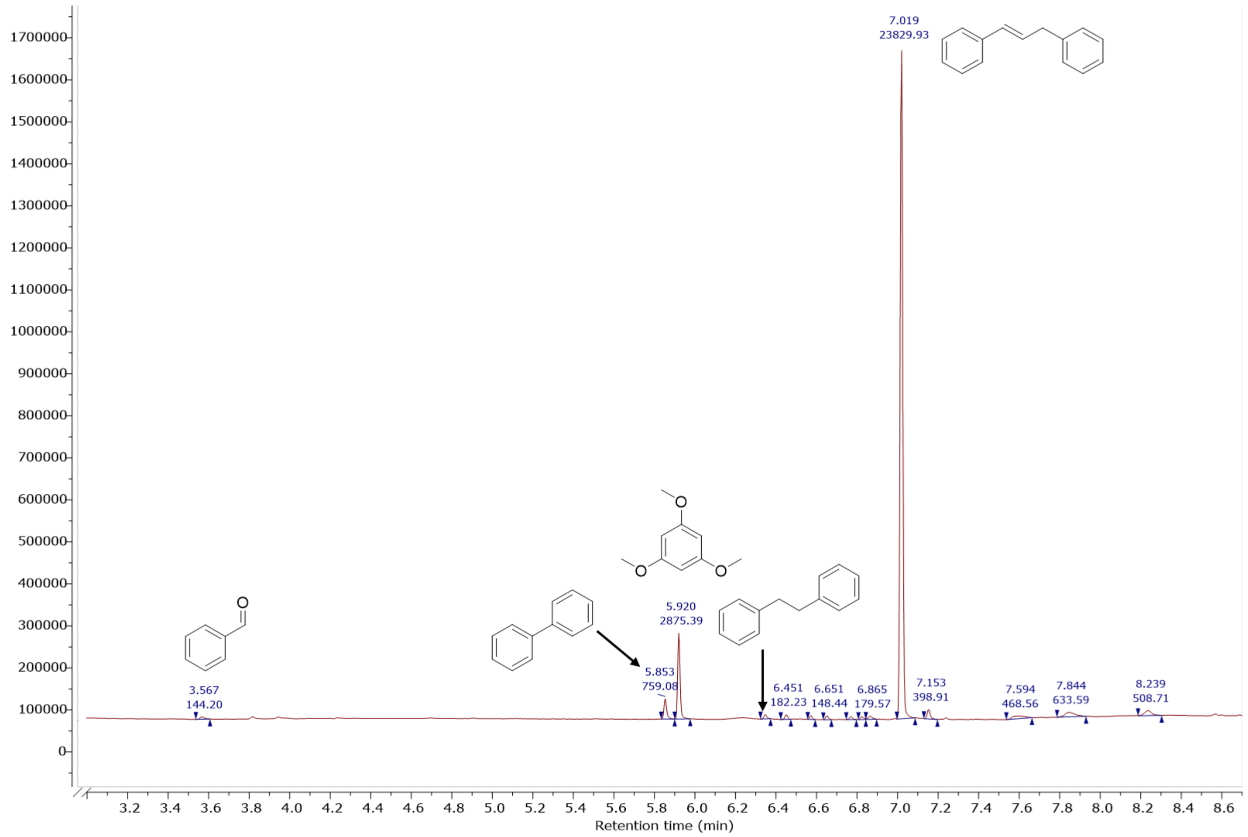

Figure S61. GC / FID data for Table 3, entry 5 in the main text. Conditions: 1 mM / 5 mol % **1**, 10 equivalents of styrene, 1 equivalent benzyl chloride, 2 equivalents Zn, 24 h reaction time, 50 °C, THF as solvent. TMB was used as an internal standard.

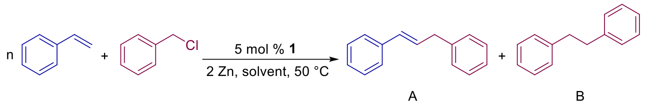

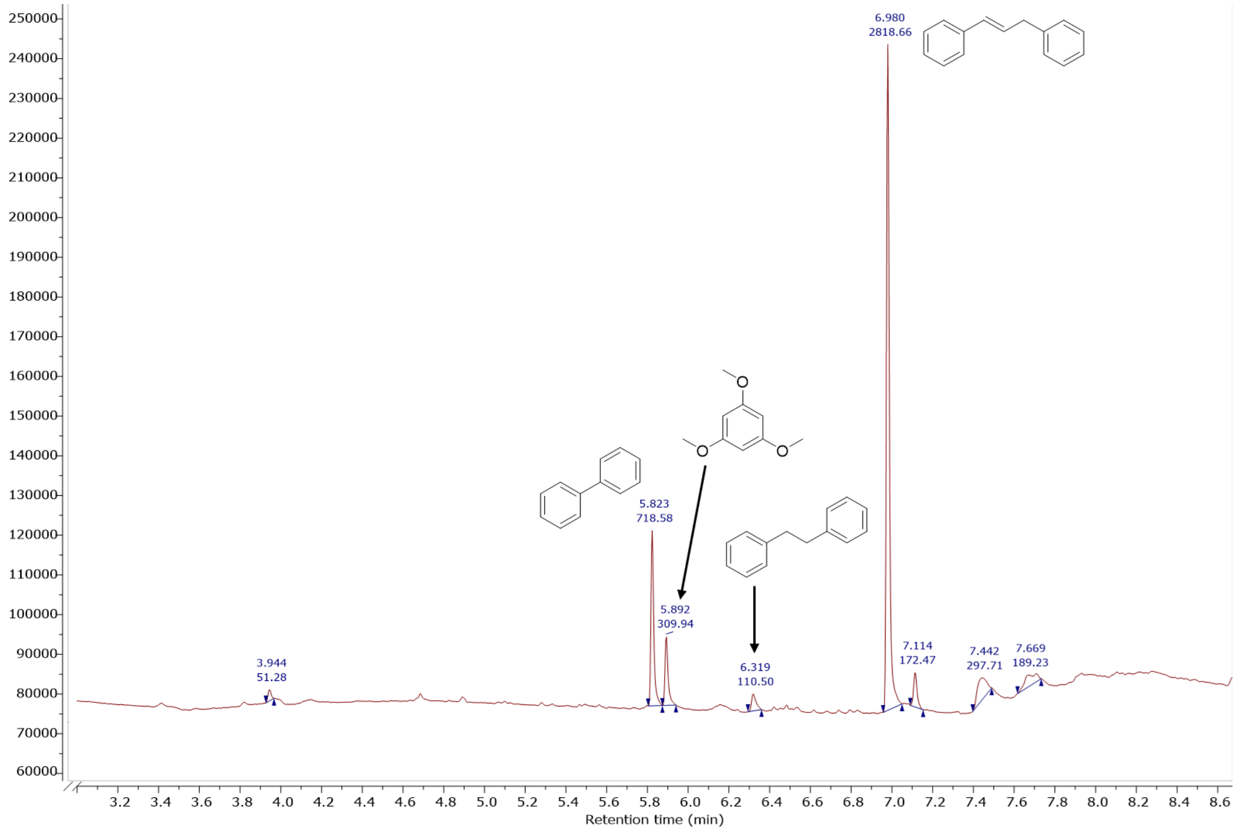

Figure S62. GC / FID data for Table 3, entry 6 in the main text. <u>Conditions:</u> 9 mM / 5 mol % **1**, 10 equivalents of styrene, 1 equivalent benzyl chloride, 2 equivalents Zn, 2 h reaction time, 50 °C, THF as solvent. TMB was used as an internal standard.

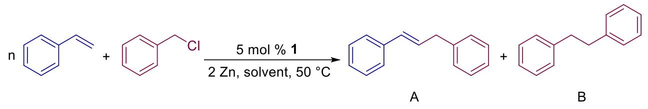

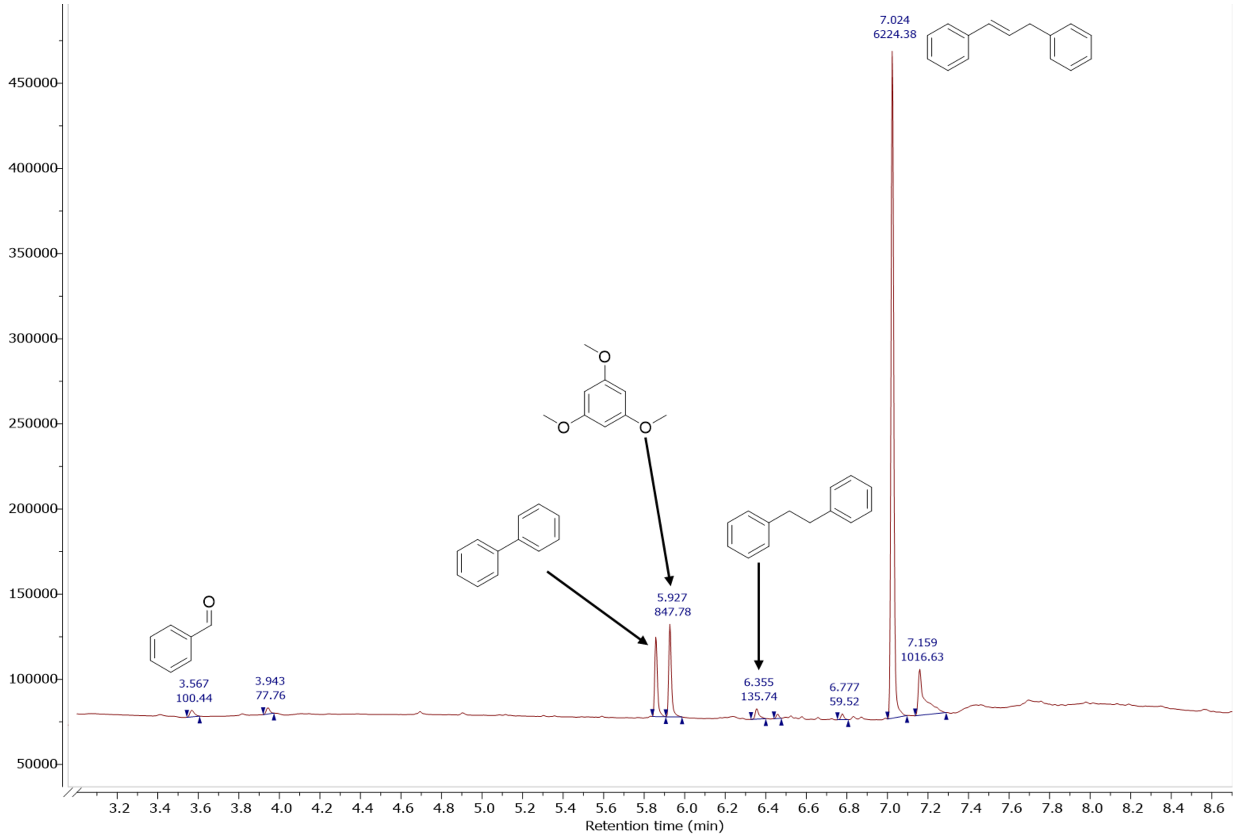

Figure S63. GC / FID data for Table 3, entry 7 in the main text. Conditions: 5 mM / 5 mol % **1**, 10 equivalents of styrene, 1 equivalent benzyl chloride, 2 equivalents Zn, 24 h reaction time, 50 °C, toluene as solvent. TMB was used as an internal standard.

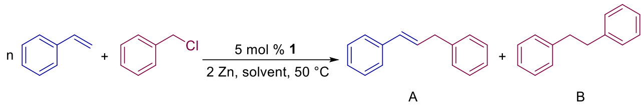

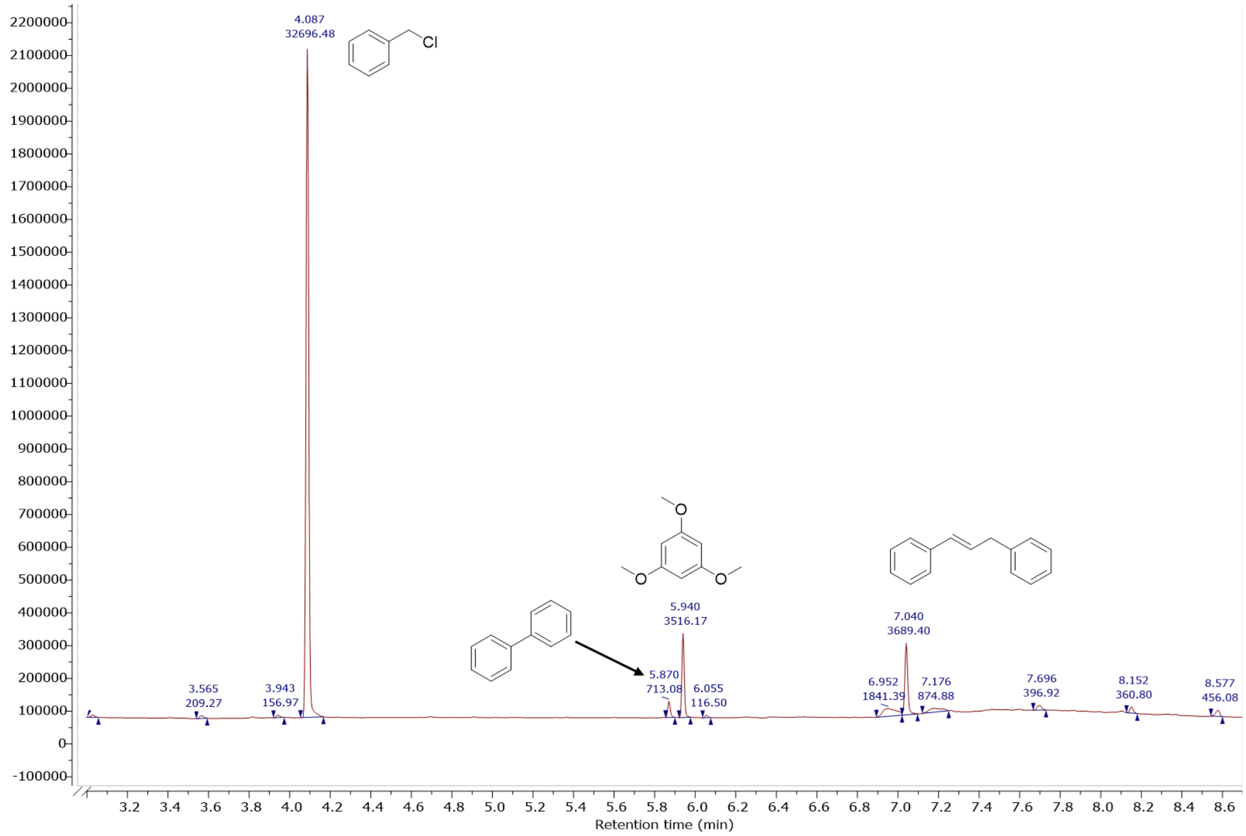

Figure S64. GC / FID data for Table 3, entry 8 in the main text. Conditions: 5 mM / 5 mol % **1**, 10 equivalents of styrene, 1 equivalent benzyl chloride, 2 equivalents Zn, 24 h reaction time, 50 °C, acetonitrile as solvent. TMB was used as an internal standard.

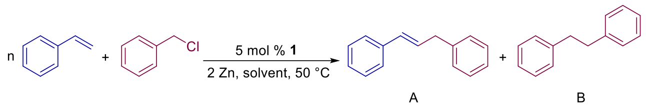

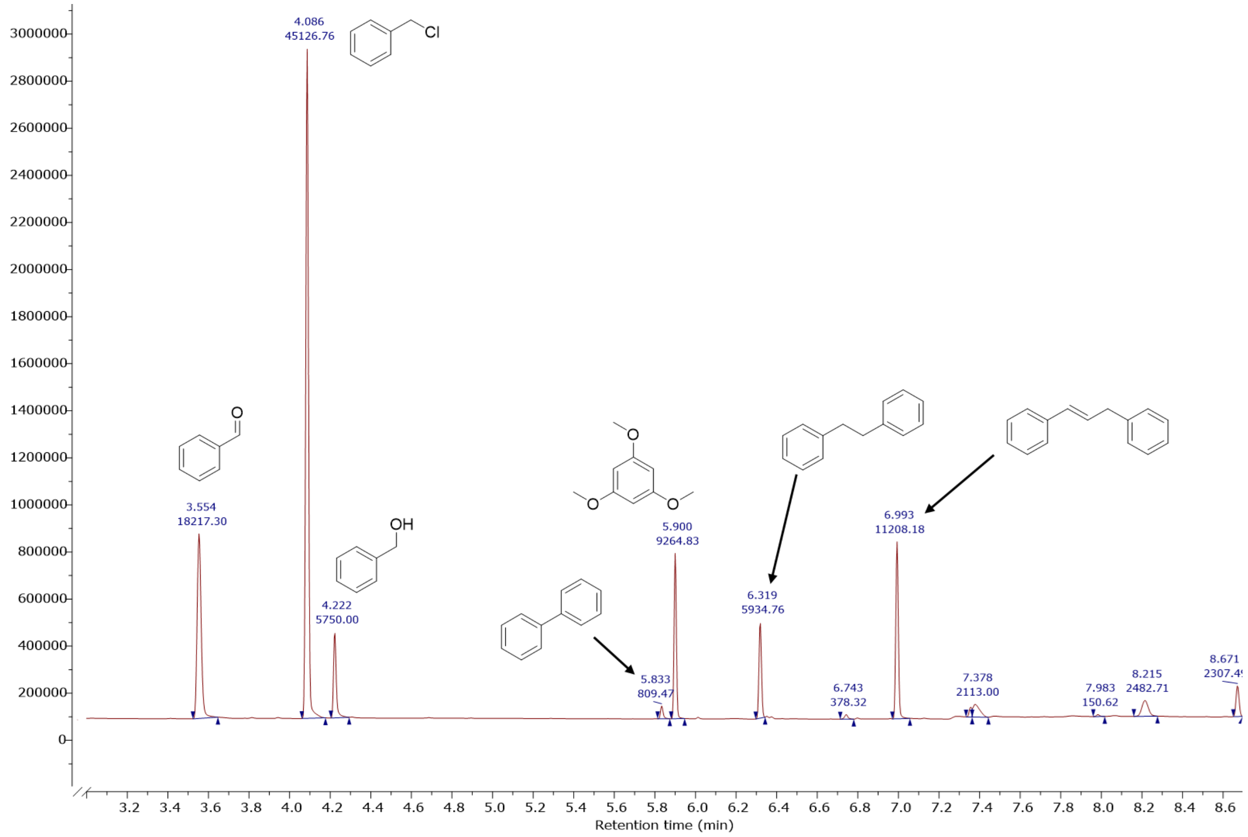

Figure S65. GC / FID data for Table 3, entry 9 in the main text. <u>Conditions:</u> 5 mM / 5 mol % **1**, 10 equivalents of styrene, 1 equivalent benzyl chloride, 2 equivalents Zn, 4 h reaction time, 50 °C, dioxane as solvent. TMB was used as an internal standard.

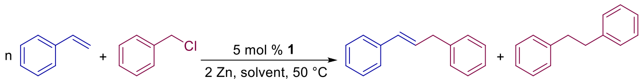

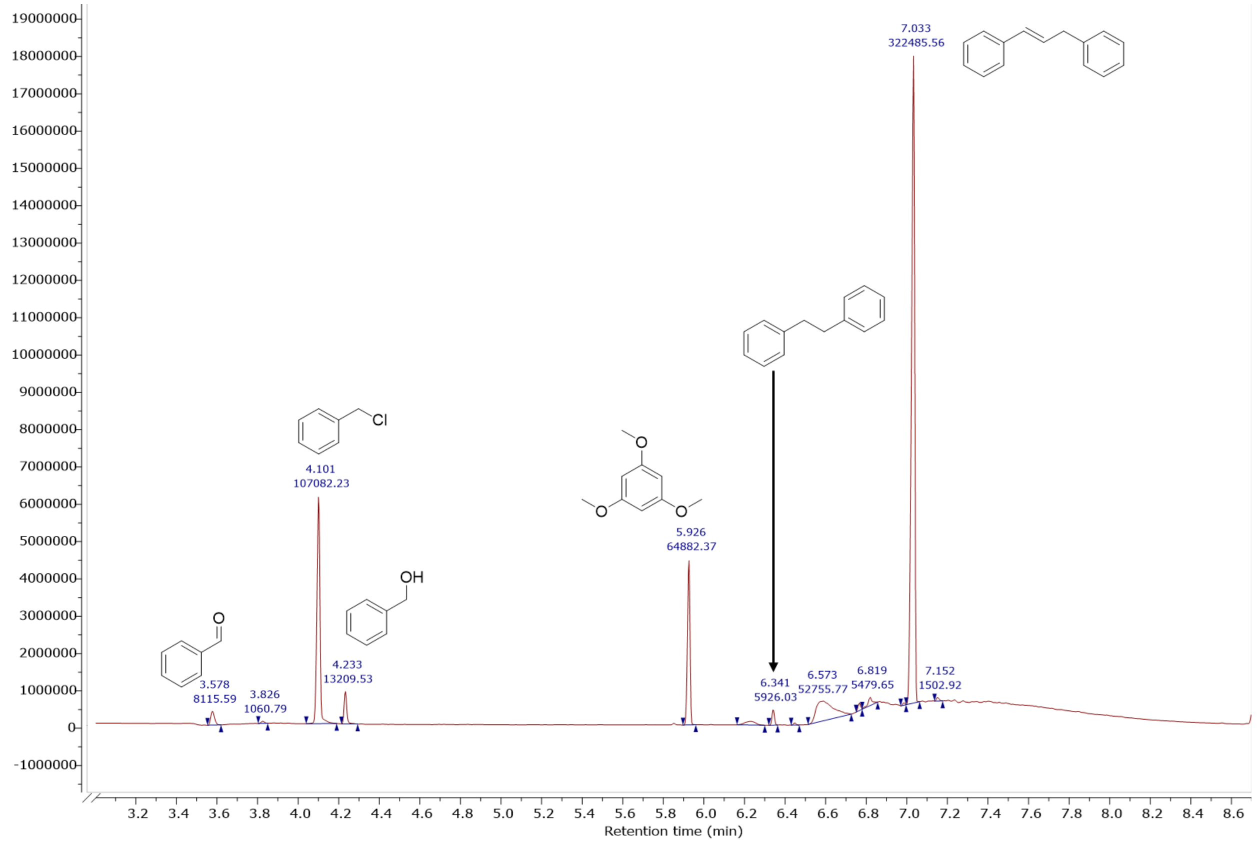

Figure S66. GC / FID data for Table 3, entry 10 in the main text. <u>Conditions:</u> 5 mM / 5 mol % **1**, 10 equivalents of styrene, 1 equivalent benzyl chloride, 2 equivalents Zn, 2 h reaction time, 40 °C, THF as solvent. TMB was used as an internal standard.

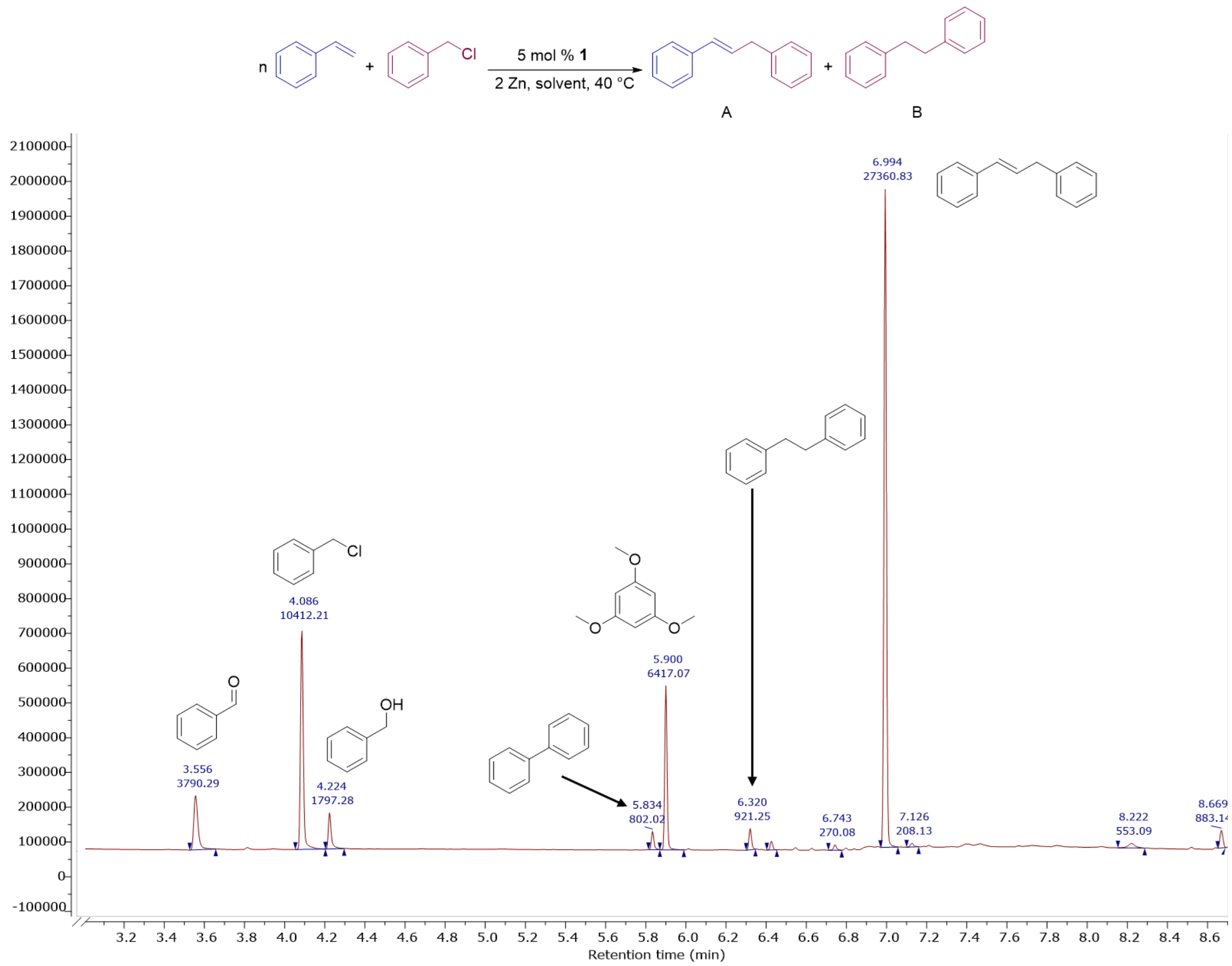

Figure S67. GC / FID data for Table 3, entry 11 in the main text. Conditions: 5 mM / 5 mol % **1**, 10 equivalents of styrene, 1 equivalent benzyl chloride, 2 equivalents Zn, 2 h reaction time, 60 °C, THF as solvent. TMB was used as an internal standard.

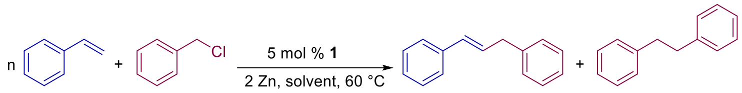

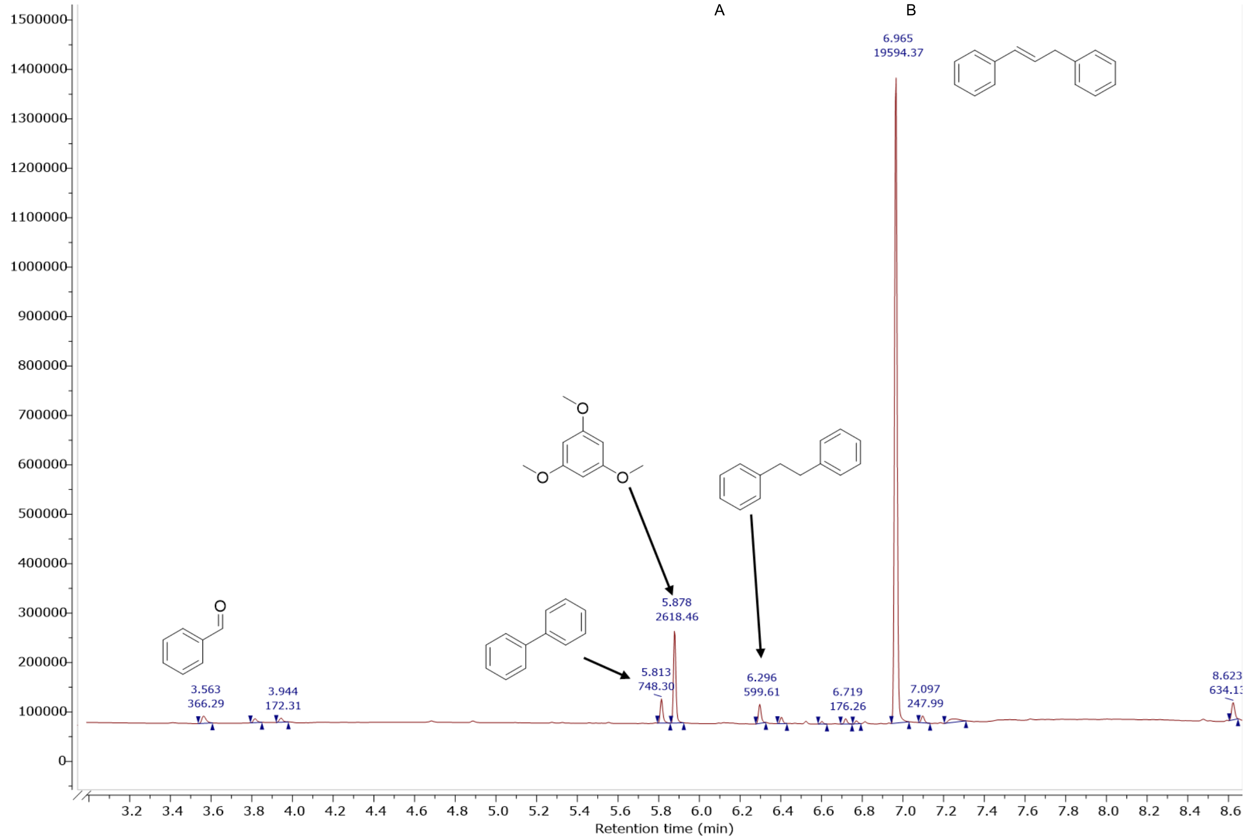

Figure S68. GC / FID data for Table 4, entry 1 in the main text. <u>Conditions:</u> 5 mM / 5 mol % **1**, 10 equivalents of styrene, 1 equivalent benzyl chloride, 2 equivalents Mg, 2 h reaction time, 50 °C, THF as solvent. TMB was used as an internal standard.

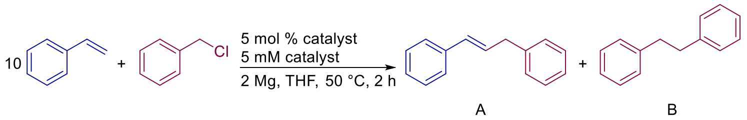

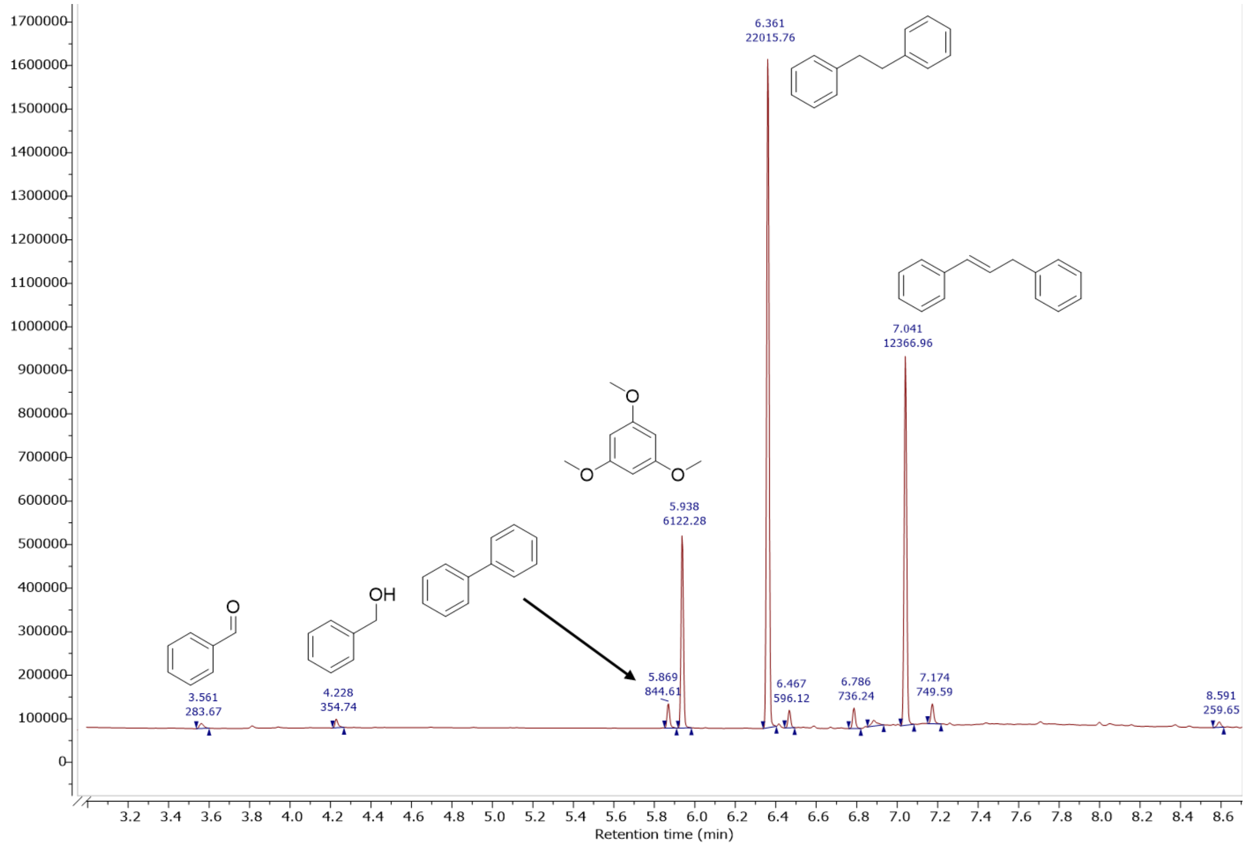

Figure S69. GC / FID data for Table 4, entry 2 in the main text. <u>Conditions:</u> 5 mM / 5 mol % **1**, 10 equivalents of styrene, 1 equivalent benzyl chloride, 5 equivalents Zn, 2 h reaction time, 50 °C, THF as solvent. TMB was used as an internal standard.

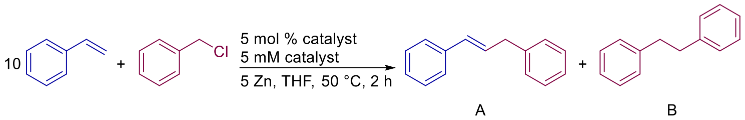

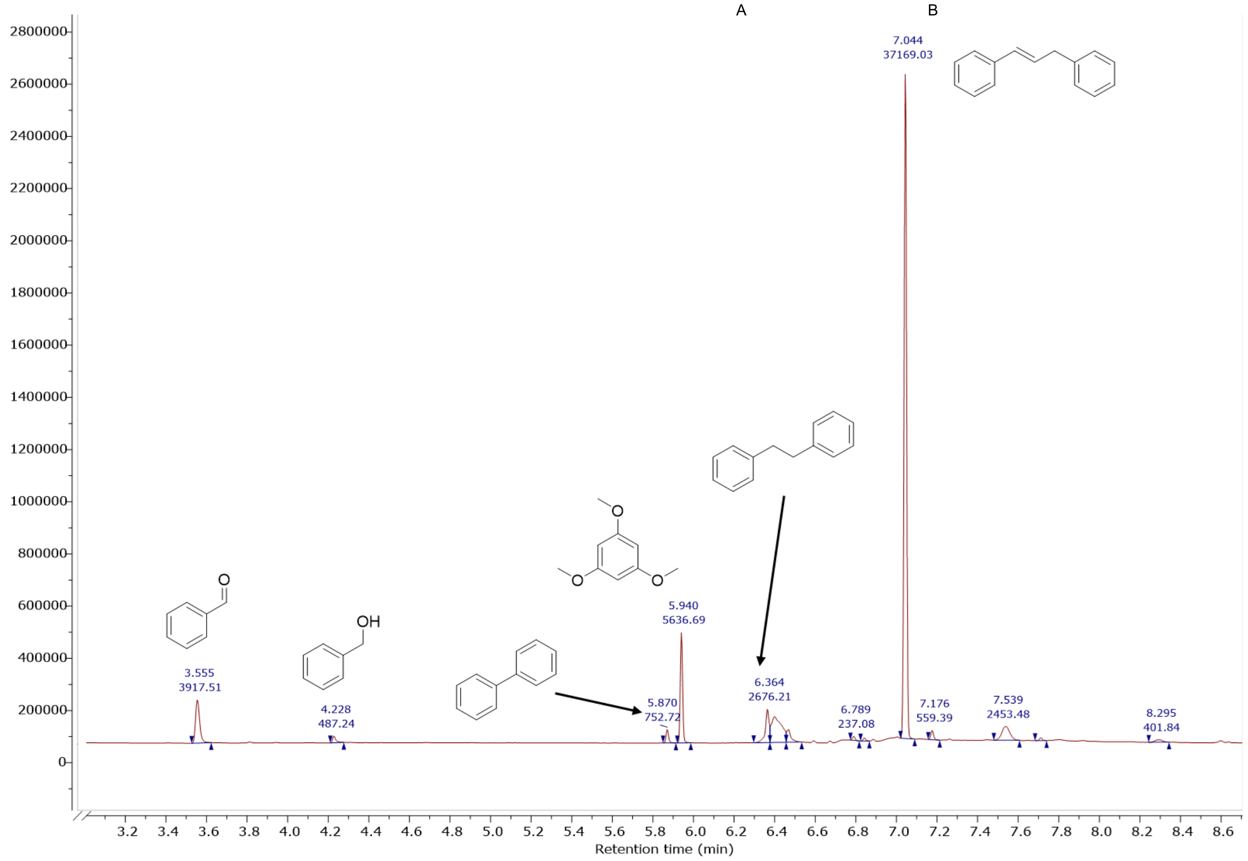

Figure S70. GC / FID data for Table 4, entry 3 in the main text. Conditions: 5 mM / 5 mol % **1**, 10 equivalents of styrene, 1 equivalent benzyl chloride, 2 equivalents Zn, 1 equivalent of NaCl, 2 h reaction time, 50 °C, THF as solvent. TMB was used as an internal standard.

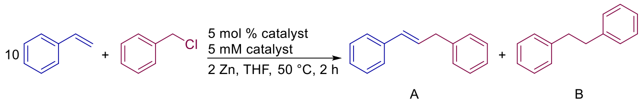

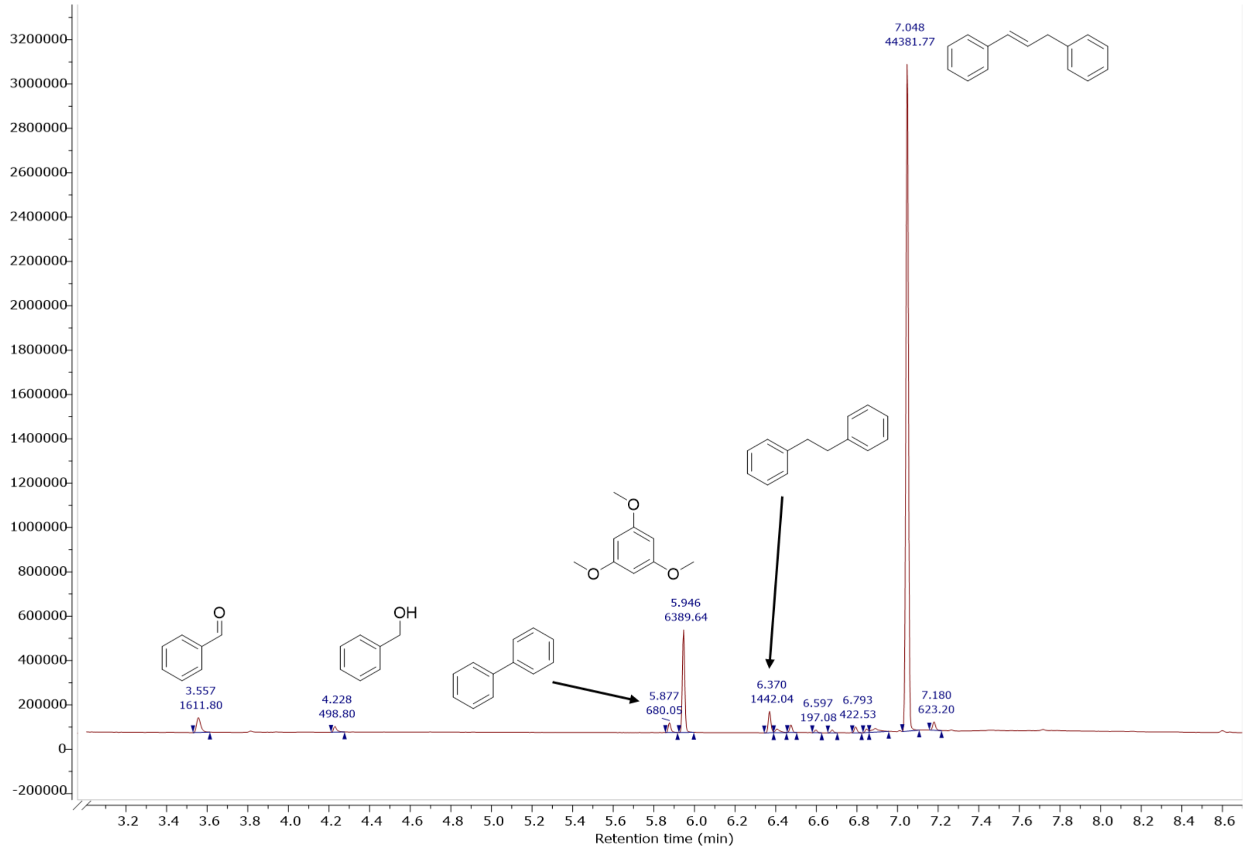

Figure S71. GC / FID data for Table 4, entry 4 in the main text. <u>Conditions:</u> 5 mM / 5 mol % **1**, 10 equivalents of styrene, 1 equivalent benzyl chloride, 2 h reaction time, 50 °C, THF as solvent. TMB was used as an internal standard.

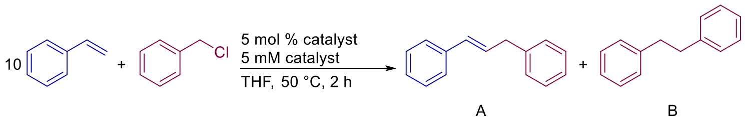

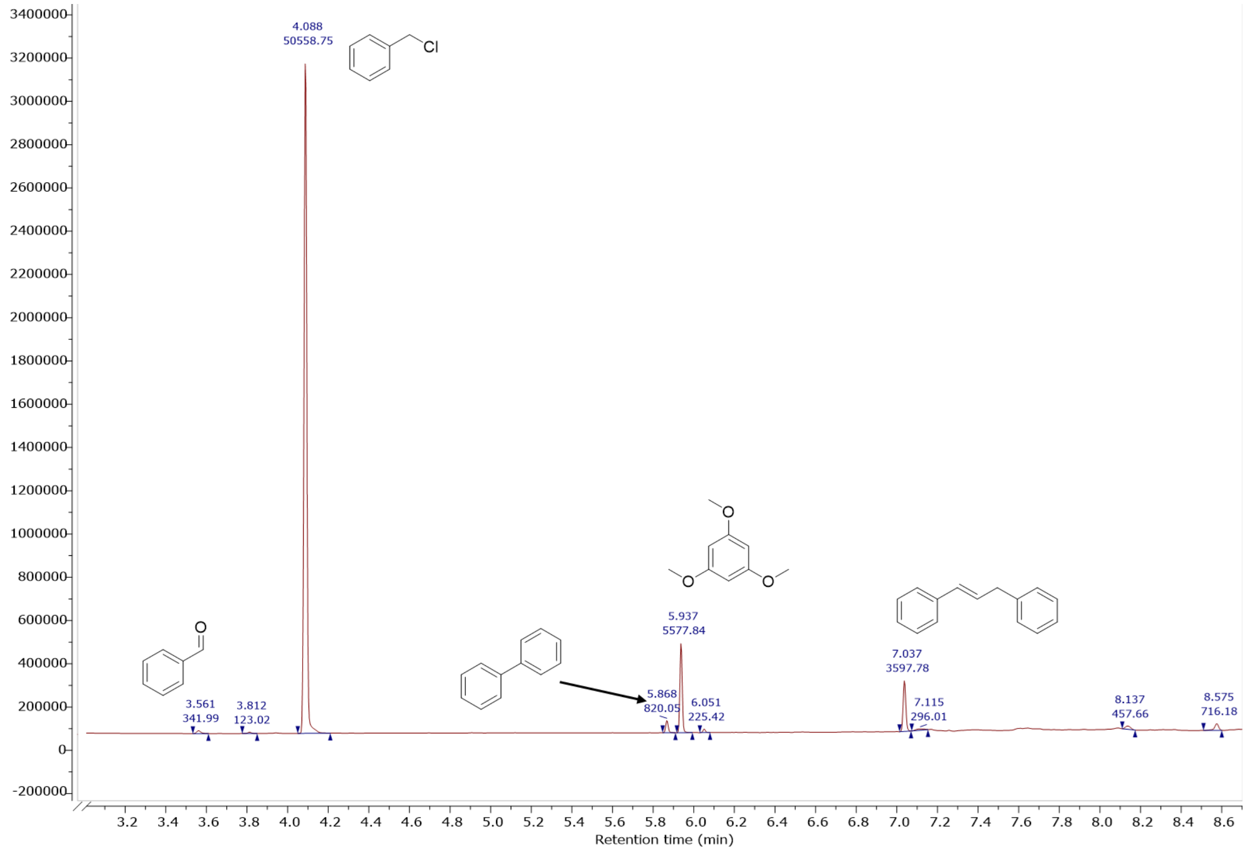

Figure S72. GC / FID data for Table 4, entry 5 in the main text. Conditions: 10 equivalents of styrene, 1 equivalent benzyl chloride, 2 equivalents Zn, 7 d reaction time, 50 °C, THF as solvent. TMB was used as an internal standard.

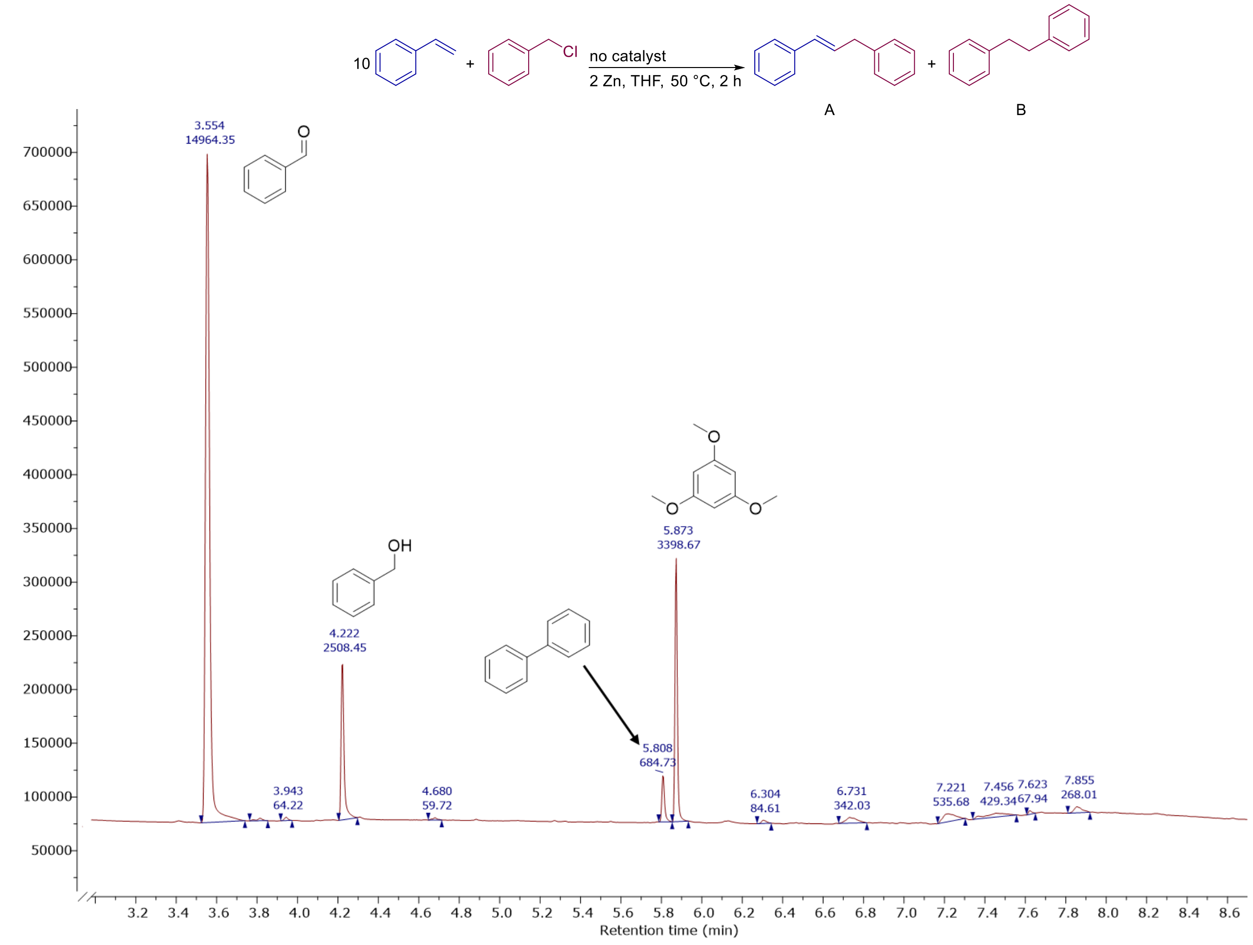

Figure S73. GC / FID data for Table 4, entry 6 in the main text. Conditions: 5 mM / 5 mol % **2**, 10 equivalents of styrene, 1 equivalent benzyl chloride, 2 equivalents Zn, 2 h reaction time, 50 °C, THF as solvent. TMB was used as an internal standard.

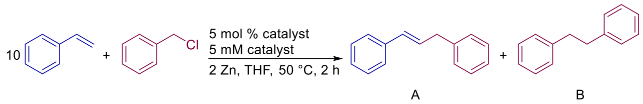

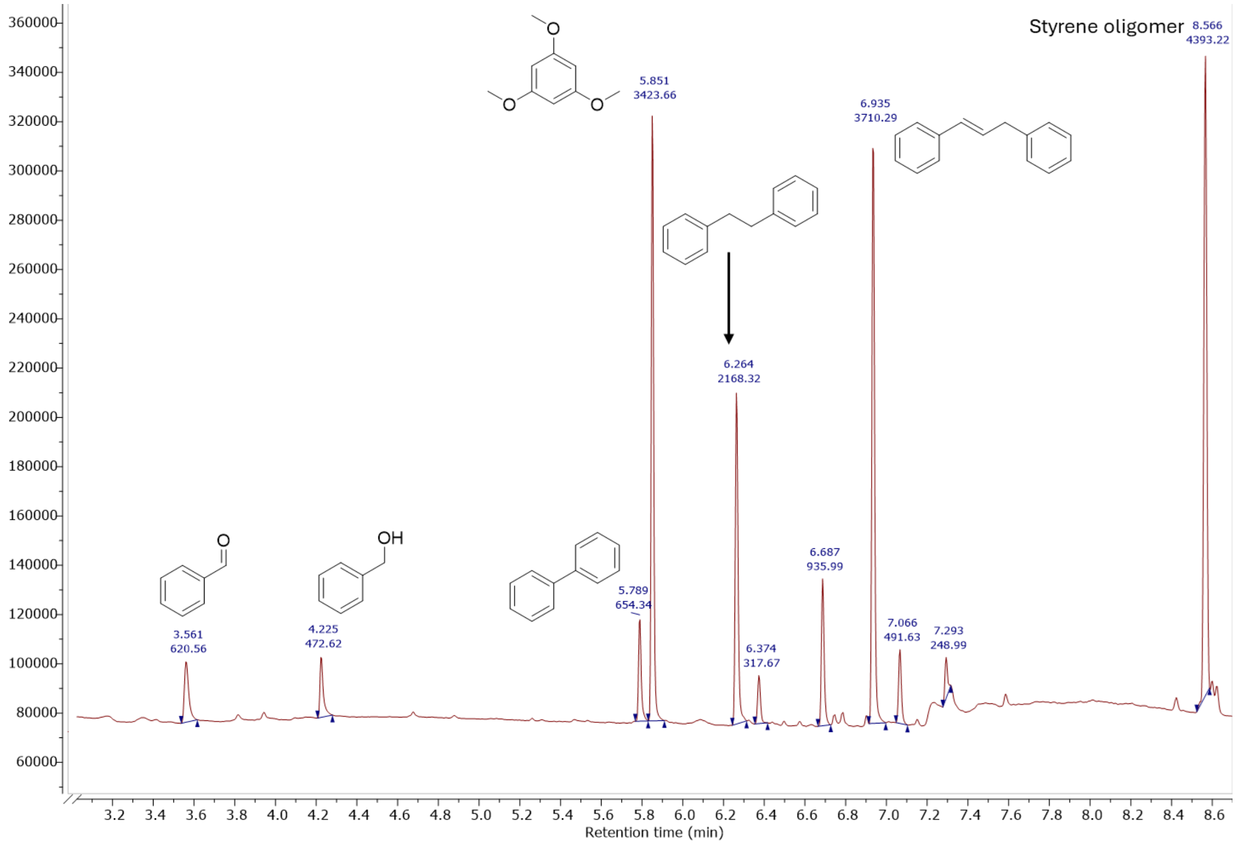

Crystal Structures

Figure S74. Fully labeled ellipsoid representation (50%) of **1-Na**.

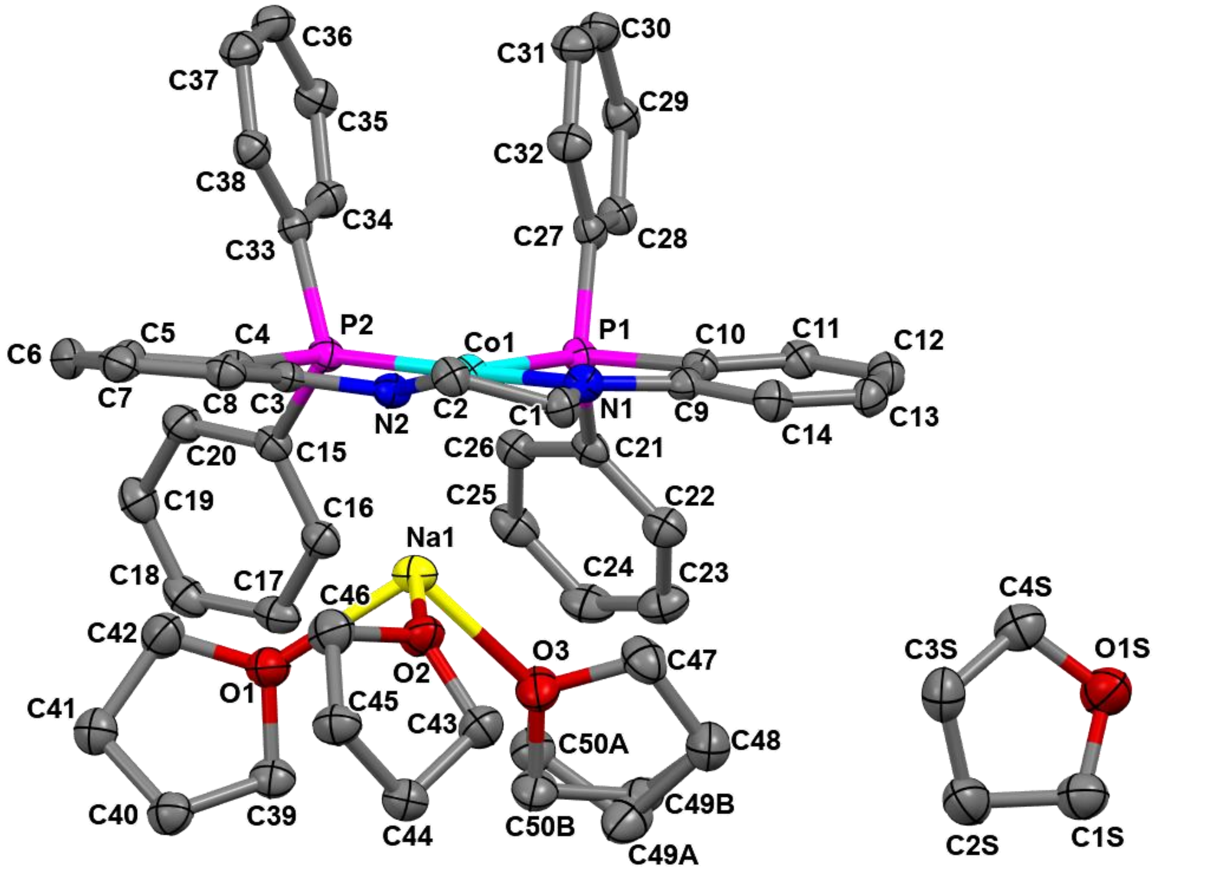

Experimental Summary

The single crystal X-ray diffraction studies were carried out on a Bruker Kappa Photon III CPAD diffractometer equipped with Mo K_α radiation (λ = 0.71073 Å). A 0.017 x 0.023 x 0.161 mm piece of a green needle was mounted on a MiTeGen MicroMount with Paratone 24EX oil. Data were collected in a nitrogen gas stream at 100(2) K using ϕ and ϖ scans. Crystal-to-detector distance was 60 mm using variable exposure time (10s-60s) depending on θ with a scan width of 0.75°. Data collection was 99.9% complete to 25.00° in θ (0.83 Å). A total of 141434 reflections were collected covering the indices, -10 ≤ h ≤ 11, -26 ≤ k ≤ 26, -28 ≤ l ≤ 28. 8795 reflections were found to be symmetry independent, with a R_{int} of 0.0993. Indexing and unit cell refinement indicated a primitive, monoclinic lattice. The space group was found to be $P2_1/n$. The data were integrated using the Bruker SAINT software program and scaled using the SADABS software program. Solution by dual-space method (SHELXT) produced a complete phasing model for refinement.

All nonhydrogen atoms were refined anisotropically by full-matrix least-squares (SHELXL-2014). All hydrogen atoms were placed using a riding model. Their positions

were constrained relative to their parent atom using the appropriate HFIX command in SHELXL-2014. Crystallographic data are summarized in Table S1.

Figure S75. Fully labeled ellipsoid representation (50%) of **2-Na**.

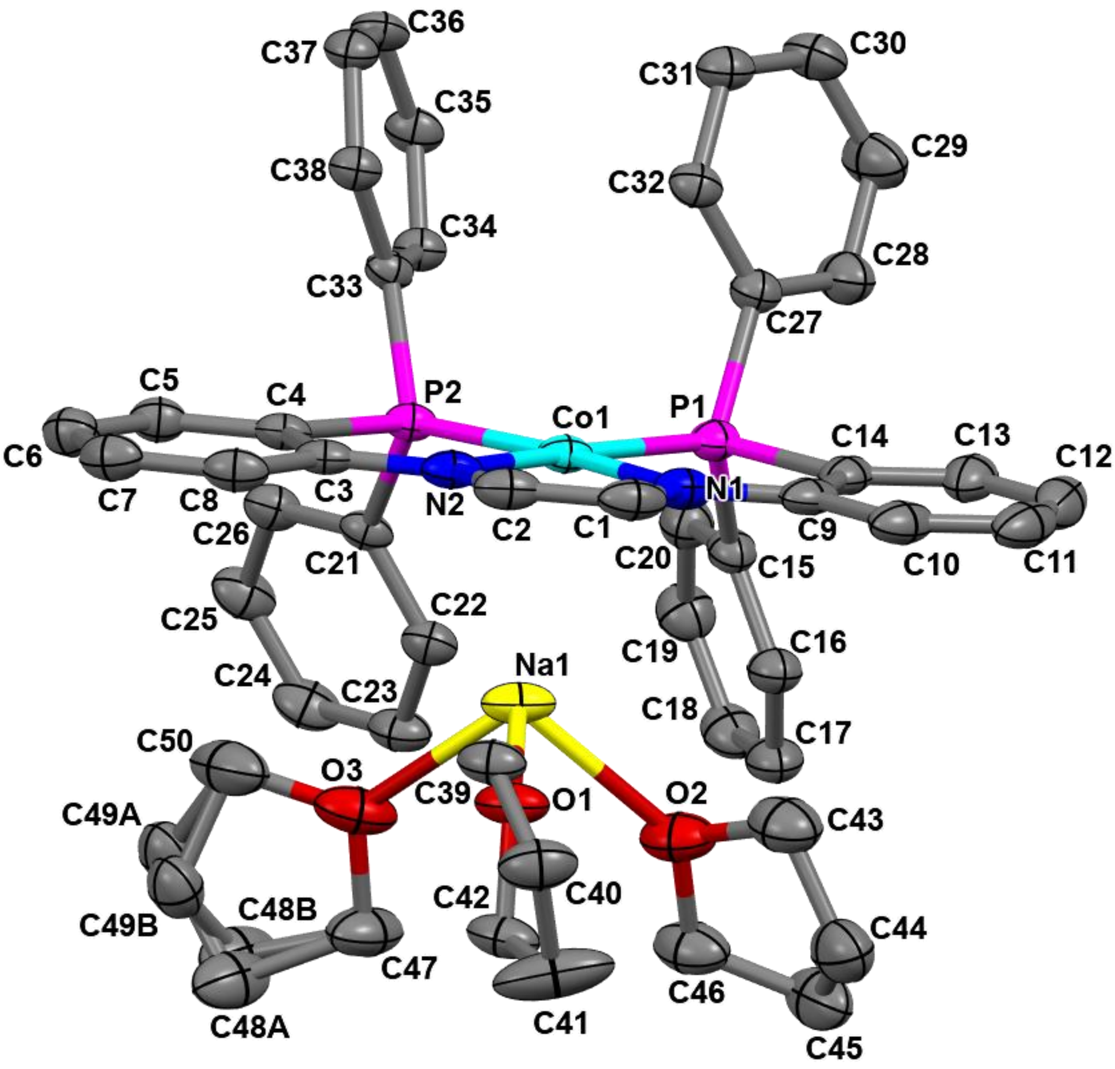

Experimental summary:

The single crystal X-ray diffraction studies were carried out on a Bruker Kappa Photon III CPAD diffractometer equipped with Mo K_α radiation (λ = 0.71073 Å). A 0.065 x 0.067 x 0.121 mm piece of a black block was mounted on a MiTeGen MicroMount with Paratone 24EX oil. Data were collected in a nitrogen gas stream at 100(2) K using ϕ and ϖ scans. Crystal-to-detector distance was 60 mm using variable exposure time (2s-10s) depending on θ with a scan width of 1.0°. Data collection was 99.8% complete to 25.00° in θ (0.83 Å). A total of 170080 reflections were collected covering the indices, $-22 \leq h \leq 23$, $-22 \leq k \leq 22$, $-21 \leq l \leq 20$. 10530 reflections were found to be symmetry independent, with a R_{int} of 0.0568. Indexing and unit cell refinement indicated a primitive, monoclinic lattice. The space group was found to be $P2_1/c$. The data were integrated using the Bruker

SAINT software program and scaled using the SADABS software program. Solution by dual-space method (SHELXT) produced a complete phasing model for refinement.

All nonhydrogen atoms were refined anisotropically by full-matrix least-squares (SHELXL-2014). All hydrogen atoms were placed using a riding model. Their positions were constrained relative to their parent atom using the appropriate HFIX command in SHELXL-2014. Due to unmodelable solvent disorder, OLEX2 solvent mask was used to remove the electron density from the lattice due to the disordered solvent contribution. Solvent appeared to be THF. One void was found to contain approximately 158 electrons, which is consistent with the presence of 1 [C_4H_8O] per formula unit and accounts for 160 electrons per unit cell. Crystallographic data are summarized in Table S1.

Figure S76. Fully labeled ellipsoid representation (50%) of **1-CH_3**.

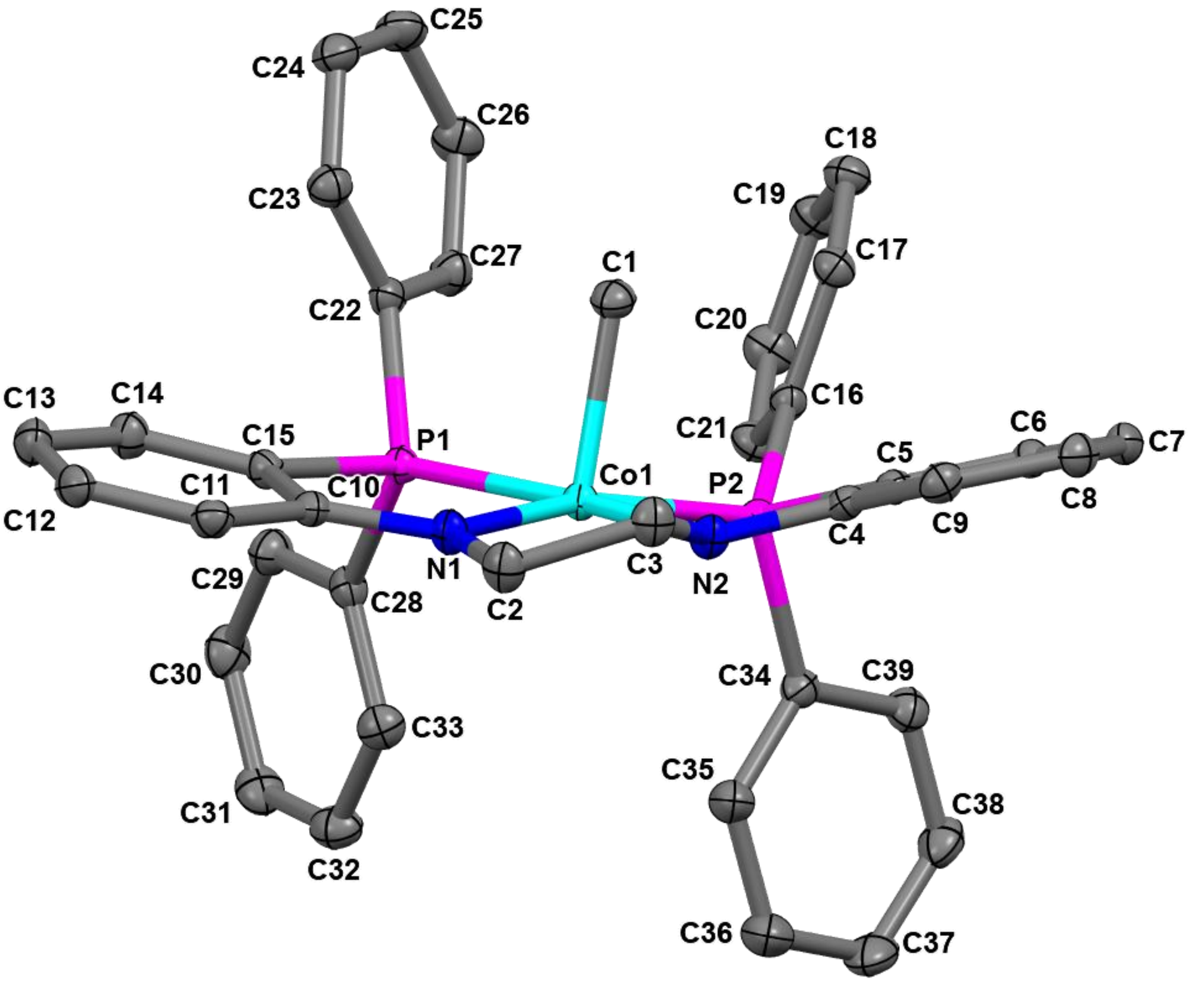

Experimental Summary:

The single crystal X-ray diffraction studies were carried out on a Bruker Kappa Photon III CPAD diffractometer equipped with Mo K_α radiation (λ = 0.71073 Å). A 0.041 x 0.125 x 0.308 mm piece of a orange prism was mounted on a MiTeGen MicroMount with Paratone 24EX oil. Data were collected in a nitrogen gas stream at 100(2) K using ϕ and ϖ scans. Crystal-to-detector distance was 60 mm using variable exposure time (1s-2s) depending on θ with a scan width of 1.0°. Data collection was 99.9% complete to 25.00° in θ (0.83 Å). A total of 114561 reflections were collected covering the indices, -15 ≤ h ≤ 15, -18 ≤ k ≤ 19, -24 ≤ l ≤ 24. 7708 reflections were found to be symmetry independent, with a R_{int} of 0.0531. Indexing and unit cell refinement indicated a primitive, monoclinic lattice. The space group was found to be *$P2_1/n$*. The data were integrated using the Bruker SAINT software program and scaled using the SADABS software program. Solution by dual-space method (SHELXT) produced a complete phasing model for refinement.

All nonhydrogen atoms were refined anisotropically by full-matrix least-squares (SHELXL-2014). All hydrogen atoms were placed using a riding model. Their positions were constrained relative to their parent atom using the appropriate HFIX command in SHELXL-2014. Crystallographic data are summarized in Table S2.

Figure S77. Fully labeled ellipsoid representation (50%) of **1-Bn**.

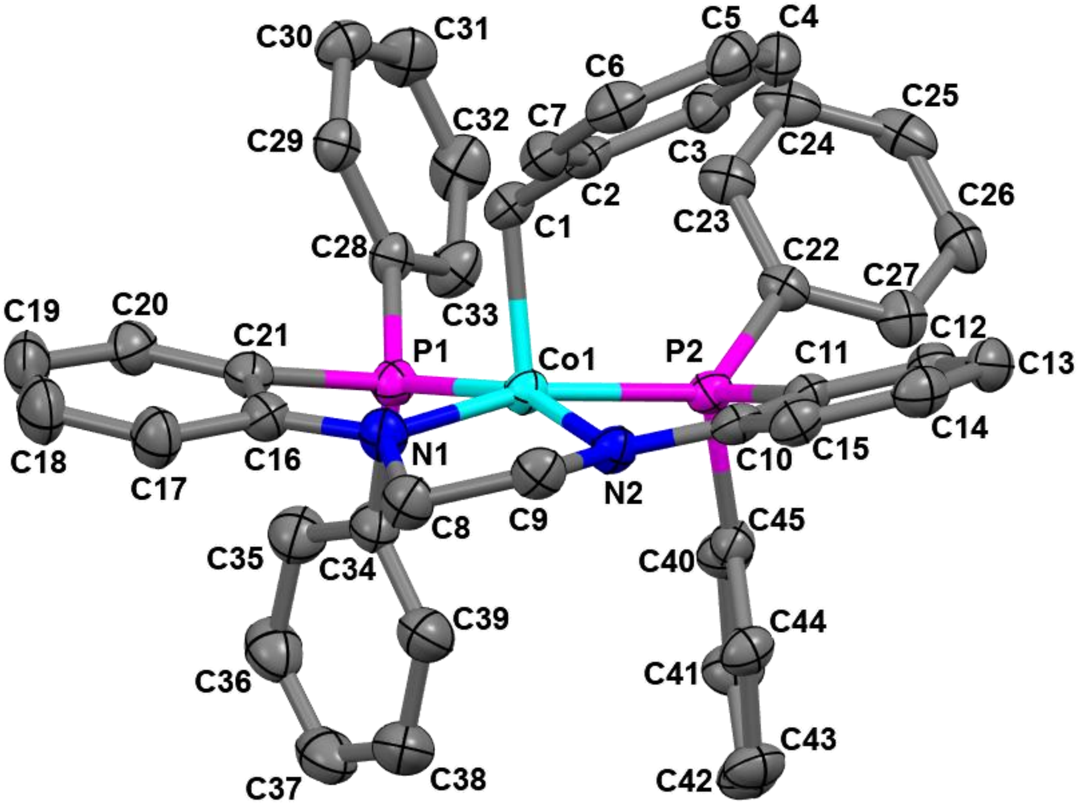

Experimental Summary:

The single crystal X-ray diffraction studies were carried out on a Bruker Kappa Photon III CPAD diffractometer equipped with Mo K_α radiation (λ = 0.71073 Å). A 0.064 x 0.067 x 0.129 mm piece of a red block was mounted on a MiTeGen MicroMount with Paratone 24EX oil. Data were collected in a nitrogen gas stream at 100(2) K using ϕ and ϖ scans. Crystal-to-detector distance was 60 mm using variable exposure time (1s-5s) depending on θ with a scan width of 1.0°. Data collection was 99.9% complete to 25.00° in θ (0.83 Å). A total of 107163 reflections were collected covering the indices, $-14 \leq h \leq 14$, $-16 \leq k \leq 16$, $-28 \leq l \leq 28$. 7416 reflections were found to be symmetry independent, with a R_{int} of 0.0744. Indexing and unit cell refinement indicated a primitive, monoclinic lattice. The space group was found to be *P2₁/c*. The data were integrated using the Bruker SAINT software program and scaled using the SADABS software program. Solution by dual-space method (SHELXT) produced a complete phasing model for refinement.

All nonhydrogen atoms were refined anisotropically by full-matrix least-squares (SHELXL-2014). All hydrogen atoms were placed using a riding model. Their positions were constrained relative to their parent atom using the appropriate HFIX command in SHELXL-2014. Due to unmodelable solvent disorder, OLEX2 solvent mask was used to remove the electron density from the lattice due to the disordered solvent contribution.

Solvent appeared to be toluene. One void was found to contain approximately 188 electrons, which is consistent with the presence of 1 [C_7H_8] per formula unit and accounts for 200 electrons per unit cell. Crystallographic data are summarized in Table S2.

Figure S78. Fully labeled ellipsoid representation (50%) of **2-CH$_3$**.

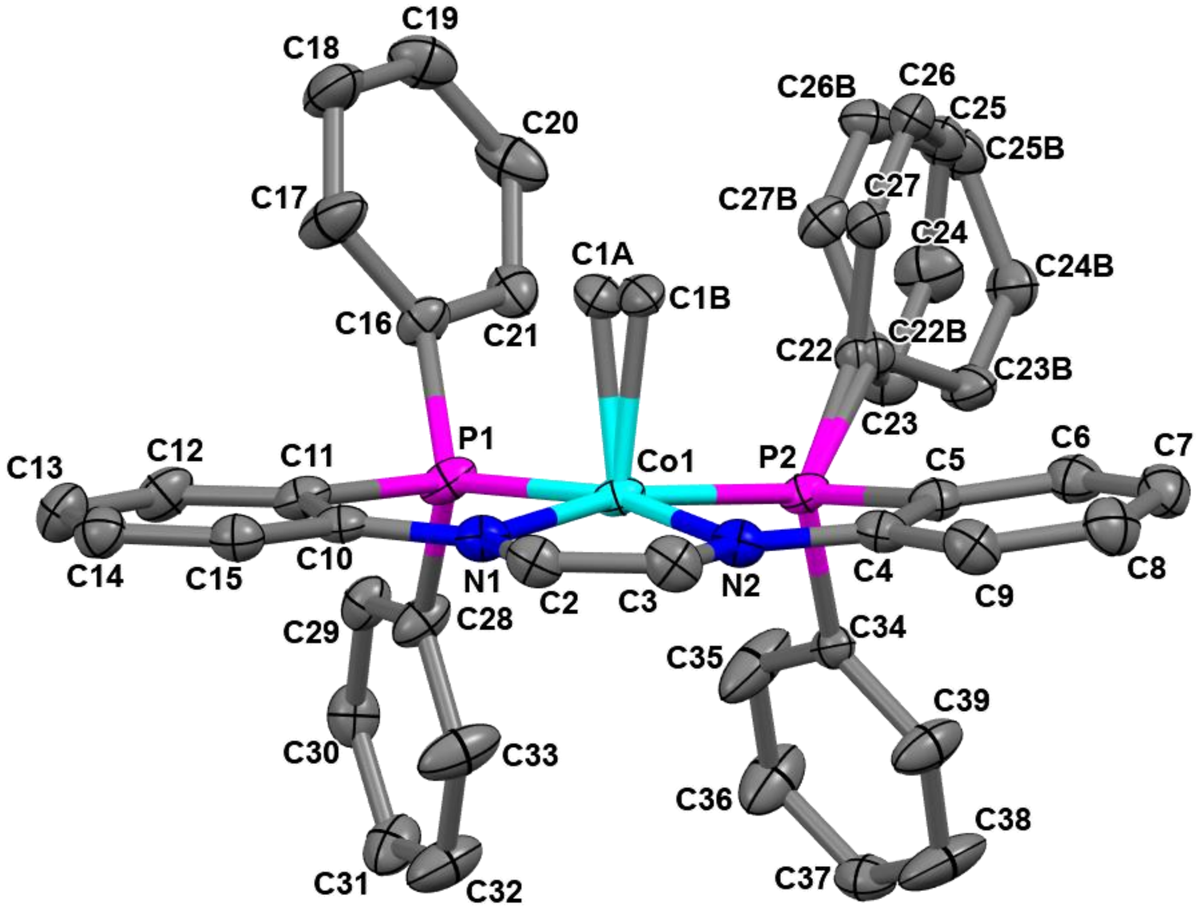

Experimental Summary:

The single crystal X-ray diffraction studies were carried out on a Bruker Kappa Photon III CPAD diffractometer equipped with Mo K_α radiation (λ = 0.71073 Å). A 0.042 x 0.088 x 0.215 mm piece of a red block was mounted on a MiTeGen MicroMount with Paratone 24EX oil. Data were collected in a nitrogen gas stream at 100(2) K using ϕ and ϖ scans. Crystal-to-detector distance was 60 mm using variable exposure time (1s-2s) depending on θ with a scan width of 1.0°. Data collection was 99.9% complete to 25.00° in θ (0.83 Å). A total of 71327 reflections were collected covering the indices, $-13 \le h \le 13$, $-14 \le k \le 14$, $-17 \le l \le 17$. 6351 reflections were found to be symmetry independent, with a R_{int} of 0.0601. Indexing and unit cell refinement indicated a primitive, triclinic lattice. The space group was found to be *P*-1. The data were integrated using the Bruker SAINT software program and scaled using the SADABS software program. Solution by dual-space method (SHELXT) produced a complete phasing model for refinement.

All nonhydrogen atoms were refined anisotropically by full-matrix least-squares (SHELXL-2014). All hydrogen atoms were placed using a riding model. Their positions

were constrained relative to their parent atom using the appropriate HFIX command in SHELXL-2014. Crystallographic data are summarized in Table S3.

Figure S79. Fully labeled ellipsoid representation (50%) of **2-Bu**.

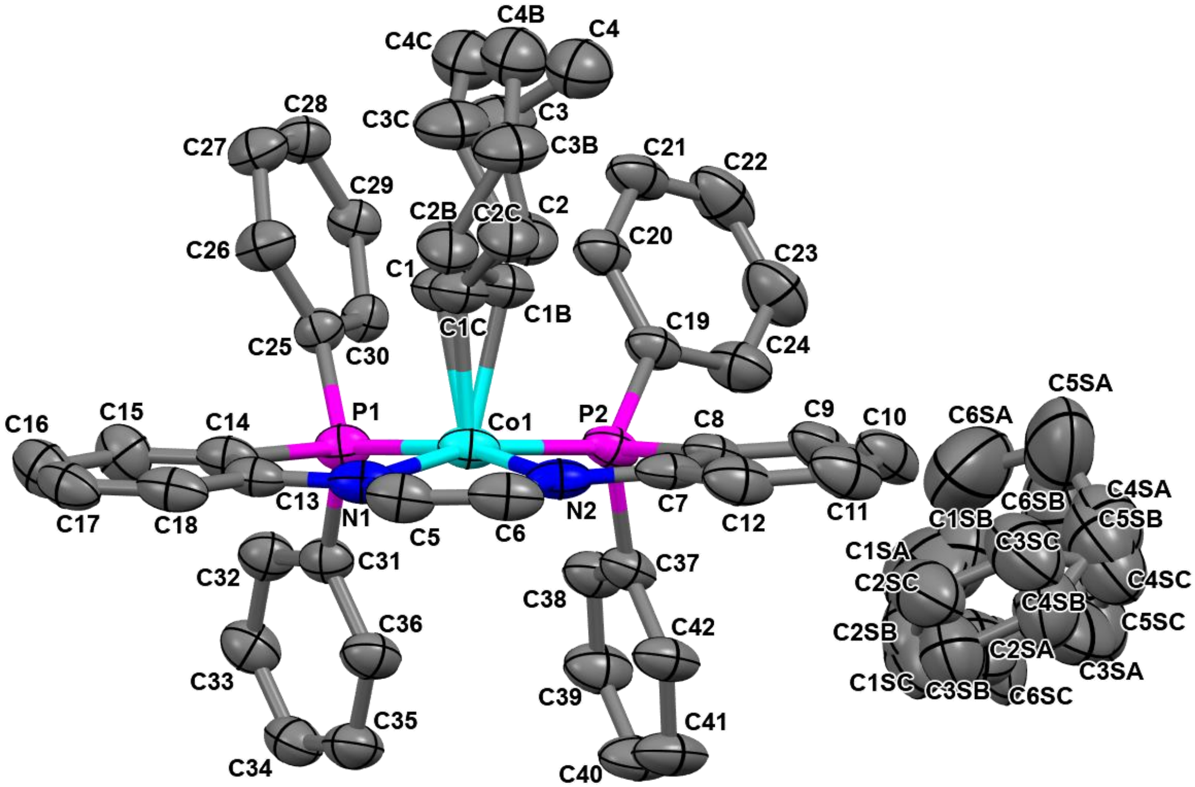

Experimental Details:

The single crystal X-ray diffraction studies were carried out on a Bruker Kappa Photon III CPAD diffractometer equipped with Mo K_{α} radiation (λ = 0.71073 Å). A 0.035 x 0.146 x 0.233 mm piece of a red plate was mounted on a MiTeGen MicroMount with Paratone 24EX oil. Data were collected in a nitrogen gas stream at 100(2) K using ϕ and ϖ scans. Crystal-to-detector distance was 60 mm using variable exposure time (2s-20s) depending on θ with a scan width of 1.0°. Data collection was 99.9% complete to 25.00° in θ (0.83 Å). A total of 96499 reflections were collected covering the indices, $-14 \le h \le 14$, $-17 \le k \le 17$, $-31 \le l \le 31$. 7948 reflections were found to be symmetry independent, with a R_{int} of 0.0532. Indexing and unit cell refinement indicated a primitive, monoclinic lattice. The space group was found to be $P2_1/n$. The data were integrated using the Bruker SAINT software program and scaled using the SADABS software program. Solution by dual-space method (SHELXT) produced a complete phasing model for refinement.

All nonhydrogen atoms were refined anisotropically by full-matrix least-squares (SHELXL-2014). All hydrogen atoms were placed using a riding model. Their positions were constrained relative to their parent atom using the appropriate HFIX command in SHELXL-2014. Crystallographic data are summarized in Table S3.

Figure S80. Fully labeled ellipsoid representation (50%) of **2-Bn**.

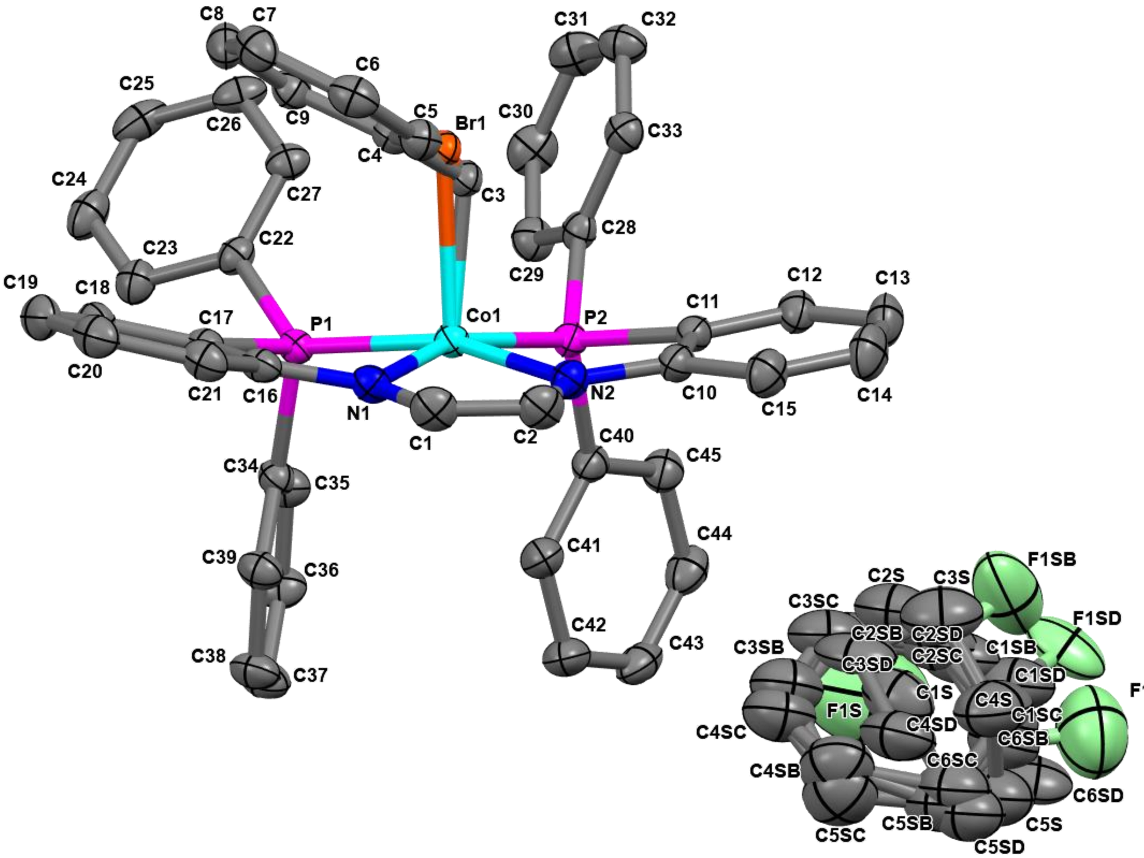

<u>Experimental Details:</u>

The single crystal X-ray diffraction studies were carried out on a Bruker Kappa Photon III CPAD diffractometer equipped with Mo K_α radiation (λ = 0.71073 Å). A 0.064 x 0.067 x 0.129 mm piece of a red block was mounted on a MiTeGen MicroMount with Paratone 24EX oil. Data were collected in a nitrogen gas stream at 100(2) K using ϕ and ϖ scans. Crystal-to-detector distance was 60 mm using variable exposure time (1s-5s) depending on θ with a scan width of 1.0°. Data collection was 99.9% complete to 25.00° in θ (0.83 Å). A total of 107163 reflections were collected covering the indices, $-14 \leq h \leq 14$, $-16 \leq k \leq 16$, $-28 \leq l \leq 28$. 7416 reflections were found to be symmetry independent, with a R_{int} of 0.0744. Indexing and unit cell refinement indicated a primitive, monoclinic lattice. The space group was found to be $P2_1/c$. The data were integrated using the Bruker SAINT software program and scaled using the SADABS software program. Solution by dual-space method (SHELXT) produced a complete phasing model for refinement.

All nonhydrogen atoms were refined anisotropically by full-matrix least-squares (SHELXL-2014). All hydrogen atoms were placed using a riding model. Their positions were constrained relative to their parent atom using the appropriate HFIX command in SHELXL-2014. Due to unmodelable solvent disorder, OLEX2 solvent mask was used to remove the electron density from the lattice due to the disordered solvent contribution. Solvent appeared to be toluene. One void was found to contain approximately 188

electrons, which is consistent with the presence of 1 [C_7H_8] per formula unit and accounts for 200 electrons per unit cell. Crystallographic data are summarized in Table S4.

Figure S81. Fully labeled ellipsoid representation (50%) of **3**.

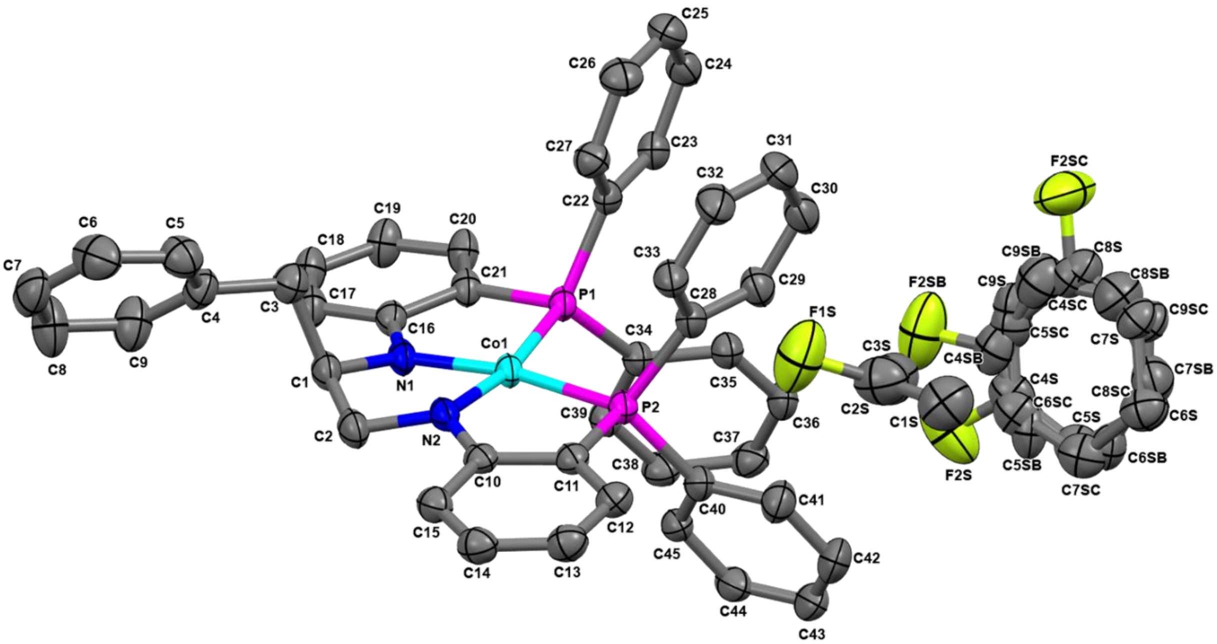

Experimental Details:

The single crystal X-ray diffraction studies were carried out on a Bruker Kappa Photon III CPAD diffractometer equipped with Mo K_α radiation (λ = 0.71073 Å). A 0.035 x 0.07 x 0.086 mm piece of a brown block was mounted on a MiTeGen MicroMount with Paratone 24EX oil. Data were collected in a nitrogen gas stream at 100(2) K using ϕ and ϖ scans. Crystal-to-detector distance was 60 mm using variable exposure time (5s-30s) depending on θ with a scan width of 1.0°. Data collection was 99.8% complete to 25.00° in θ (0.83 Å). A total of 54763 reflections were collected covering the indices, -14 ≤ h ≤ 14, -16 ≤ k ≤ 16, -19 ≤ l ≤ 19. 8706 reflections were found to be symmetry independent, with a R_{int} of 0.0481. Indexing and unit cell refinement indicated a primitive, triclinic lattice. The space group was found to be *P*-1. The data were integrated using the Bruker SAINT software program and scaled using the SADABS software program. Solution by dual-space method (SHELXT) produced a complete phasing model for refinement.

All nonhydrogen atoms were refined anisotropically by full-matrix least-squares (SHELXL-2014). All hydrogen atoms were placed using a riding model. Their positions were constrained relative to their parent atom using the appropriate HFIX command in SHELXL-2014. Crystallographic data are summarized in Table S4.

Table S1. Crystal data and structure refinement for **1-Na** and **2-Na**.

	**1-Na**	**2-Na**
CCDC number	2521559	2521560
Empirical Formula	$C_{54}H_{64}CoN_2P_2O_4P_2$	$C_{54}H_{62}CoN_2P_2O_4P_2$
Molecular Formula	$C_{50}H_{56}CoN_2O_3P_2$, C_4H_8O	$C_{50}H_{54}CoN_2P_2O_3P_2$, C_4H_8O
Formula Weight (g/mol)	948.93	946.91
Temperature (K)	100.0	100.0
Wavelength (Å)	0.71073	0.71073
Crystal System	Monoclinic	Monoclinic
Space Group	$P2_1/n$	$P2_1/c$
a (Å)<br>b (Å)<br>c (Å)<br>α (°)<br>β (°)<br>γ (°)	9.2441(5)<br>22.0319(13)<br>23.6054(14)<br>90<br>94.651(2)<br>90	18.9051(6)<br>18.0094(5)<br>16.8155(4)<br>90<br>116.2900(10)<br>90
Volume ($Å^3$)	4791.8(5)	5133.0(3)
Z	4	4
Density (calculated) (mg/m^3)	1.315	1.225
Absorption Coefficient (mm^{-1})	0.482	0.450
F(000)	2008	2000
Crystal Size (mm^3)	0.161 x 0.023 x 0.017	0.121 x 0.067 x 0.065
Crystal color, habit	green needle	black block
Final R indices [I>2sigma(I)]	R1 = 0.0382, wR2 = 0.0790	R1 = 0.0413, wR2 = 0.1114
R indices (all data)	R1 = 0.0662, wR2 = 0.0904	R1 = 0.0541, wR2 = 0.1213

Table S2. Crystal data and structure refinement for **1-CH_3** and **1-Bn**.

	**1-CH_3**	**1-Bn**
CCDC number	2521415	2521558
Empirical Formula	$C_{39}H_{35}CoN_2P_2$	$C_{52}H_{47}CoN_2P_2$
Molecular Formula	$C_{39}H_{35}CoN_2P_2$	$C_{45}H_{39}CoN_2P_2$, C_7H_8
Formula Weight (g/mol)	652.56	820.78
Temperature (K)	100.0	100.0
Wavelength (Å)	0.71073	0.71073
Crystal System	Monoclinic	Monoclinic
Space Group	$P2_1/n$	$P2_1/c$
a (Å)	11.7822(5)	12.2836(4)
b (Å)	14.3756(6)	13.9647(4)
c (Å)	18.7073(7)	23.6495(7)
α (°)	90	90
β (°)	102.6900(10)	95.5410(10)
γ (°)	90	90
Volume (Å^3)	3091.2(2)	4037.8(2)
Z	4	4
Density (calculated) (mg/m^3)	1.402	1.350
Absorption Coefficient (mm^{-1})	0.691	0.545
F(000)	1360	1720
Crystal Size (mm^3)	0.308 x 0.125 x 0.041	0.129 x 0.067 x 0.064
Crystal color, habit	orange prism	red block
Final R indices [I>2sigma(I)]	R1 = 0.0306, wR2 = 0.0725	R1 = 0.0336, wR2 = 0.0829
R indices (all data)	R1 = 0.0372, wR2 = 0.0763	R1 = 0.0445, wR2 = 0.0892

Table S3. Crystal data and structure refinement for **2-CH_3** and **2-Bu**.

	**2-CH_3**	**2-Bu**
CCDC number	2521555	2521556
Empirical Formula	$C_{39}H_{33}CoN_2P_2$	C48H45CoN2P2
Molecular Formula	$C_{39}H_{33}CoN_2P_2$	$C_{42}H_{39}CoN_2P_2$, C_6H_6
Formula Weight (g/mol)	650.54	770.73
Temperature (K)	100.0	100.0
Wavelength (Å)	0.71073	0.71073
Crystal System	Triclinic	Monoclinic
Space Group	P-1	$P2_1/n$
a (Å)	11.1807(3)	11.4489(10)
b (Å)	11.2951(4)	13.8821(11)
c (Å)	13.9365(4)	24.989(2)
α (°)	67.4060(10)	90
β (°)	72.4140(10)	103.071(3)
γ (°)	83.4460(10)	90
Volume (Å^3)	1548.98(8)	3868.7(6)
Z	2	4
Density (calculated) (mg/m^3)	1.395	1.323
Absorption Coefficient (mm^{-1})	0.689	0.563
F(000)	676	1616
Crystal Size (mm^3)	0.215 x 0.088 x 0.042	0.233 x 0.146 x 0.035
Crystal color, habit	red block	red plate
Final R indices [I>2sigma(I)]	R1 = 0.0337, wR2 = 0.0765	R1 = 0.0556, wR2 = 0.1289
R indices (all data)	R1 = 0.0432, wR2 = 0.0827	R1 = 0.0736, wR2 = 0.1404

Table S4. Crystal data and structure refinement for **2-Bn** and **3**.

	**2-Bn**	**3**
CCDC number	2521557	2521554
Empirical Formula	$C_{51}H_{42}Br_{0.01}CoFN_2P_2$	$C_{54}H_{45.5}CoF_{1.5}N_2P_2$
Molecular Formula	$C_{45}H_{37}Br_{0.012}CoN_2P_2$, C_6H_5F	$C_{45}H_{38}CoN_2P_2$, 1.5(C_6H_5F)
Formula Weight (g/mol)	823.73	871.79
Temperature (K)	100.0	100.0
Wavelength (Å)	0.71073	0.71073
Crystal System	Monoclinic	Triclinic
Space Group	$P2_1/c$	P-1
a (Å)	12.2668(4)	11.9753(8)
b (Å)	13.9713(4)	13.1828(8)
c (Å)	23.7370(6)	15.2408(9)
α (°)	90	101.327(2)
β (°)	96.0030(10)	110.176(2)
γ (°)	90	101.051(2)
Volume ($Å^3$)	4045.8(2)	2124.6(2)
Z	4	2
Density (calculated) (mg/m^3)	1.352	1.363
Absorption Coefficient (mm^{-1})	0.559	0.527
F(000)	1714	908
Crystal Size (mm^3)	0.259 x 0.199 x 0.108	0.086 x 0.07 x 0.035
Crystal color, habit	black block	brown block
Final R indices [I>2sigma(I)]	R1 = 0.0360, wR2 = 0.1000	R1 = 0.0429, wR2 = 0.1057
R indices (all data)	R1 = 0.0414, wR2 = 0.1051	R1 = 0.0630, wR2 = 0.1175

Table S5. Selected bond distances for **2**, **2-CH₃**, **2-Bu**, **2-Bn**, and **2-PF₆**.

**Compound**	**C-N$_{avg}$** (Å)	**C-C** (Å)	**Co-N$_{avg}$** (Å)
**2-CH_3**	1.379(3)	1.347(3)	1.8797(18)
**2-Bu**	1.386(6)	1.343(5)	1.872(4)
**2-Bn**	1.376(3)	1.351(3)	1.8836(19)
**2**	1.403(3)	1.396(5)	1.878(2)
**2-PF_6**	1.337(7)	1.391(6)	1.908(4)

Cyclic Voltammetry

Figure S82. (a) Scan-rate dependence studies for **1-CH_3** at 100 mV/s (i_{pc} = *7.6* μA , i_{pa} = -7.5 μA) and 500 mV/s (i_{pc} = 14.4 μA , i_{pa} = -13.6 μA). (b) Scan-rate dependence studies for **2-CH_3** at 100 mV/s (i_{pc} = *15.1* μA , i_{pa} = -14.1 μA) and 500 mV/s (i_{pc} = *28.6* μA , i_{pa} = -27.3 μA)

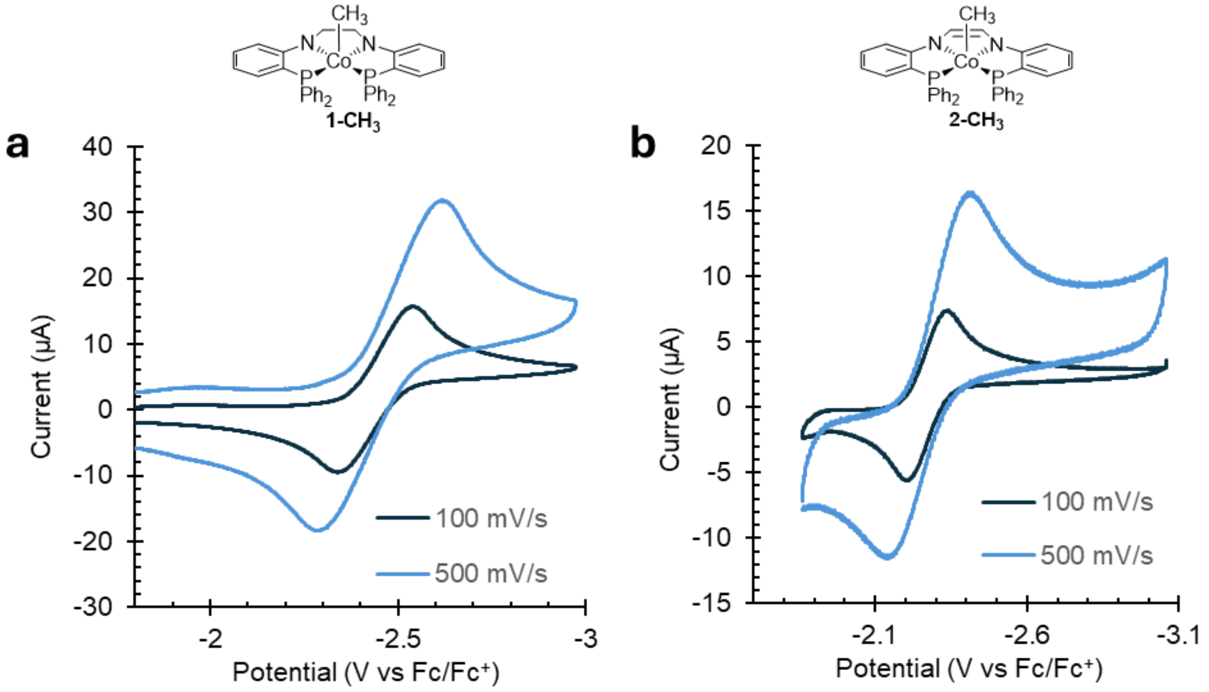

Computational Studies

Scheme S2. General reaction used to calculate cobalt-carbon bond dissociation enthalpy (BDE) values.

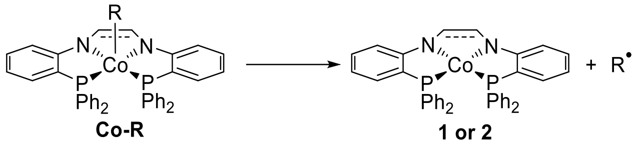

Table S6. Computed energies (converted to kcal/mol) of molecules used for calculating cobalt-carbon BDE values. The calculated BDE values are shown in the far-right column.

Compound	Enthalpy Co-R (kcal/mol)	Enthalpy Co^{II} (kcal/mol)	Enthalpy $R^{\cdot}$ (kcal/mol)	$E_{Co\text{-}R} - (E_{Co(II)} + E_{R\cdot})$ (kcal/mol)
**1-CH_3**	-2311026.912	-2285992.06	-24995.55096	39.3
**1-Bu**	-2385028.378	-2285992.06	-98996.3527	40.0
**1-Bn**	-2456059.204	-2285992.06	-170032.3939	34.7
**2-CH_3**	-2310288.713	-2285251.63	-24995.55096	41.5
**2-Bu**	-2384290.708	-2285251.63	-98996.3527	42.7
**2-Bn**	-2455323.431	-2285251.63	-170032.3939	39.4
**(salen)Co-Bu**	-1518025.966	-1418995.239	-98996.3527	34.4
